# Large-scale profiling of noncoding RNA function in yeast

**DOI:** 10.1371/journal.pgen.1007253

**Published:** 2018-03-12

**Authors:** Steven Parker, Marcin G. Fraczek, Jian Wu, Sara Shamsah, Alkisti Manousaki, Kobchai Dungrattanalert, Rogerio Alves de Almeida, Edith Invernizzi, Tim Burgis, Walid Omara, Sam Griffiths-Jones, Daniela Delneri, Raymond T. O’Keefe

**Affiliations:** 1 Manchester Institute of Biotechnology, The University of Manchester, Manchester, United Kingdom; 2 Faculty of Biology, Medicine and Health, The University of Manchester, Manchester, United Kingdom; Stanford University School of Medicine, UNITED STATES

## Abstract

Noncoding RNAs (ncRNAs) are emerging as key regulators of cellular function. We have exploited the recently developed barcoded ncRNA gene deletion strain collections in the yeast *Saccharomyces cerevisiae* to investigate the numerous ncRNAs in yeast with no known function. The ncRNA deletion collection contains deletions of tRNAs, snoRNAs, snRNAs, stable unannotated transcripts (SUTs), cryptic unstable transcripts (CUTs) and other annotated ncRNAs encompassing 532 different individual ncRNA deletions. We have profiled the fitness of the diploid heterozygous ncRNA deletion strain collection in six conditions using batch and continuous liquid culture, as well as the haploid ncRNA deletion strain collections arrayed individually onto solid rich media. These analyses revealed many novel environmental-specific haplo-insufficient and haplo-proficient phenotypes providing key information on the importance of each specific ncRNA in every condition. Co-fitness analysis using fitness data from the heterozygous ncRNA deletion strain collection identified two ncRNA groups required for growth during heat stress and nutrient deprivation. The extensive fitness data for each ncRNA deletion strain has been compiled into an easy to navigate database called Yeast ncRNA Analysis (YNCA). By expanding the original ncRNA deletion strain collection we identified four novel essential ncRNAs; SUT527, SUT075, SUT367 and SUT259/691. We defined the effects of each new essential ncRNA on adjacent gene expression in the heterozygote background identifying both repression and induction of nearby genes. Additionally, we discovered a function for SUT527 in the expression, 3’ end formation and localization of *SEC4*, an essential protein coding mRNA. Finally, using plasmid complementation we rescued the SUT075 lethal phenotype revealing that this ncRNA acts in *trans*. Overall, our findings provide important new insights into the function of ncRNAs.

## Author summary

Genomes from different organisms produce noncoding RNAs that are not translated to make proteins and whose functions are largely unknown. There are approximately 2,000 noncoding RNAs that make up almost 25% of the yeast genome compared to the approximately 6,000 protein coding genes that make up 70% of the yeast genome. With this large number of ncRNAs there is a need for large-scale studies to determine the functional roles of ncRNAs. We take advantage of a recently developed resource of 532 yeast strains in which individual noncoding RNA genes have been deleted. We grow these yeast noncoding RNA deletion strains in different conditions and catalogue how each noncoding RNA contributes to cell growth. Improvement or inhibition of cell growth under particular conditions implicates the deleted RNA in cellular responses to those conditions. We have also investigated in more detail the function of specific noncoding RNAs, revealing examples of how a deletion influences nearby genes, and other examples of noncoding RNAs that regulate genes at distant genomic locations. We have made our extensive data on the fitness of noncoding RNA deletion mutants publically available for searching and bulk download in a new online resource, called Yeast ncRNA Analysis (YNCA). This large-scale analysis of noncoding RNA deletion mutants reveals the importance of many noncoding RNAs in cellular function.

## Introduction

Eukaryotic cells express a wide variety of RNAs that do not code for proteins but contribute to the many essential functions within cells. The process of protein synthesis by translation requires ribosomal RNAs (rRNAs) to form the ribosomal subunits and transfer RNAs (tRNAs) to bring the amino acids to the ribosome [1,2]. Another class of RNAs called small nucleolar RNA (snoRNAs) predominantly catalyze the modification or processing of other RNAs, but additional novel functions for snoRNAs are emerging [3]. The small nuclear RNAs (snRNAs) of the spliceosome are required for the recognition and removal of introns from pre-messenger RNA [4]. The functions of most of these so called classical noncoding RNAs (ncRNAs) have been known for some time.

More recently, expression analysis of eukaryotic genomes has established that pervasive transcription produces an abundance of ncRNAs whose functions are largely unknown [5–9]. In human cells, where some ncRNA functions are known, there tends to be three mechanistic themes for ncRNA function where ncRNAs act as either decoys to titrate proteins away from their binding sites, scaffolds to bring proteins together or guides to recruit proteins to DNA [10]. A number of methods to probe the functional significance of the numerous ncRNAs in humans have been utilized. For example, ncRNA gene deletion, targeting ncRNAs with RNAi and repression of ncRNA transcription with CRISPR based methods are just a few techniques used to investigate the functions of expressed human ncRNAs [11–15]. Mutations in ncRNAs are also increasingly being associated with human diseases [16–18].

In the yeast *Saccharomyces cerevisiae*, tiling arrays and strand-specific RNA sequencing analyses have identified novel classes of ncRNAs that are distinct from the classical ncRNAs. Two classes of ncRNAs were initially identified according to their half-life in the cell, the stable unannotated transcripts (SUTs) had a relatively long half-life whereas the cryptic unstable transcripts (CUTs) were RNAs with a short half-life and were revealed only after deletion of the exosome complex exoribonuclease Rrp6 [9,19]. Deletion of the cytoplasmic exonuclease Xrn1, followed by RNA sequencing, revealed another class of ncRNAs termed Xrn1-sensitive unstable transcripts (XUTs) [20,21], some of which overlap with either a SUT or CUT. Subsequently, depletion of the RNA binding factor Nrd1 revealed a fourth class of ncRNA termed Nrd1-unterminated transcripts (NUTs) [22] and deletion of the histone methyltransferase Set2 has identified yet another class of ncRNA called the Set2-repressed antisense transcripts (SRATs) [23]. With the numbers of these yeast ncRNAs in the thousands only a very small proportion have been ascribed a function to date.

Where there are examples of ncRNA function in yeast one emerging theme is that ncRNA transcription can either induce or repress the expression of an adjacent gene [24–27]. One mechanism whereby ncRNA expression can induce or repress nearby gene expression is through chromatin modification [28]. An investigation into the influence of transcription by 180 anti-sense SUTs on the overlapping yeast genes found no direct relationship between antisense SUT transcription and protein abundance from the overlapping reading frame, indicating that the presence of an antisense SUT does not necessarily mean it regulates protein abundance from the sense protein coding gene [29]. Analysis of six intergenic SUTs using the synthetic genetic array (SGA) technology, to identify genetic interactions between deletions of these six SUTs and non-essential protein deletion strains, linked two SUTs to specific cellular functions and provided evidence that they may function in *trans* [30]. Many of the SUTs and CUTs are associated with specific RNA binding proteins within the yeast cell that are distinct from those bound by mRNAs to presumably allow them to carry out their specific function [31]. There is also evidence from ribosome profiling techniques that some yeast unannotated ncRNAs associate with ribosomes and can be translated into protein, so may not necessarily be noncoding [32–34]. As many of these studies have only investigated the function of a small subset of ncRNAs, a large scale analysis of ncRNA function in yeast would be useful for defining the role in the cell of the remaining ncRNAs.

We have utilized the recently developed collection of ncRNA deletion strains [35], which we have now expanded further, to carry out large-scale functional analysis of ncRNAs in yeast. In total 532 different ncRNA deletions were investigated encompassing tRNAs, snRNAs, snoRNAs, SUTs, CUTs and other annotated ncRNAs that do not overlap protein coding genes. Using both the heterozygous and haploid ncRNA deletion strain collections we have analyzed quantitatively, in a variety of growth conditions and phases, the influence that deletion of each ncRNA has on cellular fitness. This fitness analysis identified novel environmental-dependent haplo-proficient and haplo-insufficient growth phenotypes which provided key information on ncRNA function. Additionally, we have analyzed four essential ncRNAs of unknown function and have determined how deletion of these ncRNAs influenced surrounding gene expression. Moreover, we identified one ncRNA that works in *trans* and characterized a more detailed function for one of these ncRNAs in regulating the expression, 3’ end formation and localization of an essential protein coding mRNA. Overall, these data significantly expand the information available on the function of ncRNAs in yeast. Finally, the extensive catalog of functional data has been compiled into an easy to use website called YNCA providing an important resource for future ncRNA research.

## Results

### Expanding the ncRNA deletion collections

The ncRNA deletion strain collections, as previously reported, contained 428 heterozygous diploid deletion strains in the reference strain BY4743, 373 haploid (*MATa*), 370 haploid (*MATα*) and 331 homozygous diploid ncRNA deletion strains giving a total of 1502 strains for functional analysis of ncRNAs [35]. Each ncRNA, that did not overlap with a protein coding gene, was deleted with the *KanMX* cassette while simultaneously introducing two unique molecular barcodes to allow identification of each deletion strain. We have now expanded this collection by the addition of 81 heterozygous diploid, 66 haploid (*MATa*), 67 haploid (*MATα*) strains and 63 homozygous diploid ncRNA deletion strains to give a total of 1779 strains (**S1 and S2 Tables**). Within these collections 532 different individual ncRNAs have been deleted in at least one strain background. These new combined collections of strains were utilized for fitness profiling to determine how ncRNA deletion affected the growth of cells under a variety of conditions.

### Fitness profiling of the heterozygous ncRNA deletion collection

**Fig 1:**
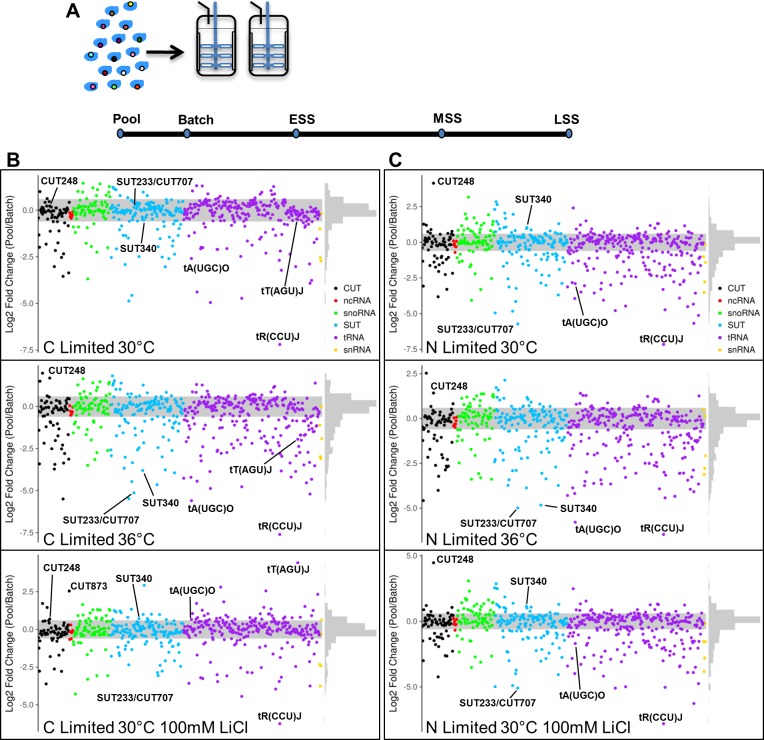
Diagram of competition experiment and analysis of the pool to batch fitness changes. (**A**) The pool of heterozygous deletion strains was grown in batch culture before the switch to continuous culture. Samples were taken at the initial pool stage, the batch stage, early steady state (ESS), mid steady state (MSS) and late steady state (LSS). (**B**) Comparison of fitness between the pool and batch stages under the three indicated carbon-limited conditions. Haplo-proficient deletion strains have positive Log2 fold change and haplo-insufficient deletion strains have negative Log2 fold change. (**C**) Comparison of fitness between the pool and batch stages under the three indicated nitrogen-limited conditions. Haplo-proficient deletion strains have positive Log2 fold change and haplo-insufficient deletion strains have negative Log2 fold change. Any strains falling outside the grey shaded area have a significant fitness difference (p < 0.05). Graphs where individual points can be identified are found in **S4–S9 Tables**.

To quantify the impact of ncRNAs on cellular fitness, competition experiments were carried out using the heterozygous deletion collection, with the deletion strains pooled and grown in six different liquid media. Two biological repeats were carried out for each condition. After an initial batch phase, the strains were propagated in continuous culture (steady state), an open system in which the amount of nutrients and pH are kept constant, allowing small fitness differences to be detected [36]. Specifically, cells were grown under carbon-limited and nitrogen-limited conditions at both 30°C and 36°C. Cells were also grown under carbon-limited and nitrogen-limited conditions at 30°C in the presence of 100mM LiCl which is known to inhibit the exoribonuclease Xrn1 and stabilize RNA [37]. Culture samples were removed for analysis at the beginning (initial pool, P) and end of the batch growth (B), at early steady state (ESS), mid steady state (MSS) and late steady state (LSS) time points (**Fig 1A**) to compare the composition of these populations with each other. Genomic DNA from each sample was isolated and the unique molecular barcodes identifying each deletion strain were amplified for next generation sequencing (Bar-Seq) [38–40] to determine the abundance of each ncRNA deletion strain in the population. As there were two biological repeats a total of four independent barcodes were sequenced for each ncRNA deletion strain. Under-representation of specific deletion strains highlights haplo-insufficient phenotypes, namely ncRNAs that are quantitatively important for phenotypic maintenance. Over-representation of deletion strains (haplo-proficient phenotypes) suggest that lowering the copy number of specific ncRNAs is beneficial in that particular environmental context.

We first compared the population fitness profile between the initial pool and batch stage to identify strains that displayed either haplo-insufficiency or haplo-proficiency in the six different conditions tested (**Fig 1B and 1C; S3–S10 Tables**). The tRNA, tR(CCU)J, also known as *HSX1*, displayed an extreme haplo-insufficient phenotype between the pool and batch stages in all six conditions we tested. The tRNA tR(CCU)J is a single copy rare tRNA gene [41] and is clearly required for batch growth of yeast. The reduced fitness of the tR(CCU)J deletion strain from pool to batch indicates that the function of tR(CCU)J is critical when nutrients become limiting. Reduced fitness of the tR(CCU)J deletion strain was also validated in monoculture under nutrient rich (YPD), carbon-limited and nitrogen-limited conditions at 30°C (**S1 Fig**). Another tRNA, tA(UGC)O, displayed haplo-insufficiency between the pool and the batch stage in both carbon-limited and nitrogen-limited conditions, but only at 36°C (**Fig 1B and 1C; S10 Table**), suggesting that this tRNA is required for fitness under conditions of heat stress.

**Fig 2:**
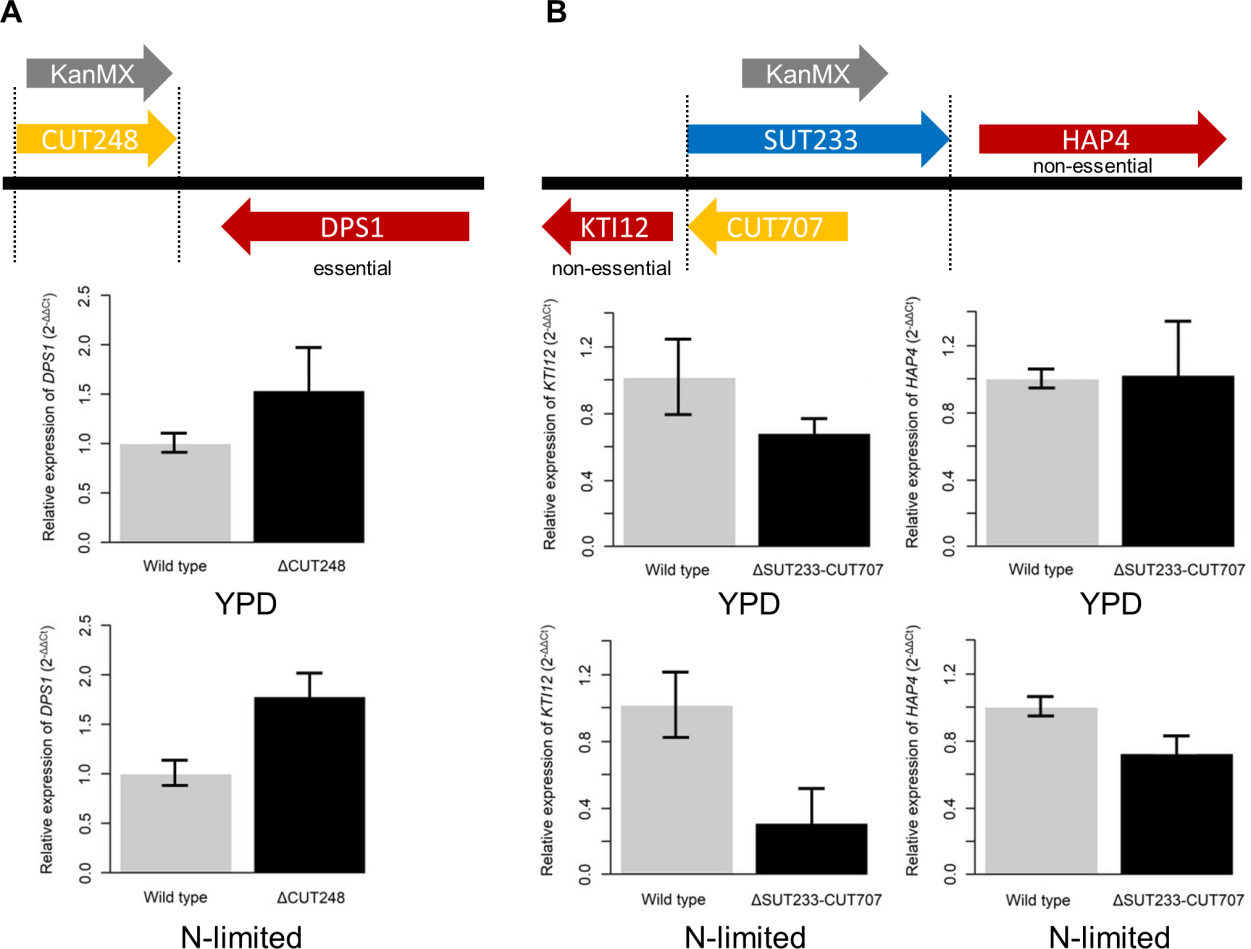
Genome location of ncRNA deletions and RT-PCR quantitation of mRNA levels. (**A**) Genome location of CUT248 compared to *DPS1*. All of CUT248 was deleted. Relative expression of *DPS1* in the wild type diploid background is represented by grey bars and *DPS1* expression in the CUT248 diploid heterozygous deletion background is represented by black bars. Cells were grown in either rich media (YPD) or N-limited conditions. *DPS1*: YPD *p* = 0.006, N-limited p = <0.01. (**B**) Genome location of SUT233/CUT707 between the *HAP4* and *KTI12* genes. Relative expression of *HAP4* or *KTI12* in the wild type diploid background is represented by grey bars and *HAP4* or *KTI12* expression in the SUT233/CUT707 diploid heterozygous deletion background is represented by black bars. Cells were grown in either rich media (YPD) or N-limited conditions. *HAP4*: YPD *p* = 0.97, N-limited p = <0.01. *KTI12*: YPD *p* = 0.05, N-limited p = <0.01. The fold change (2^) in expression, relative to the wild-type was calculated using the ΔΔCт method and *ACT1* as a reference gene. Error bars were calculated using three independent biological samples. P values were calculated using the Welch two sample t-test.

A number of the heterozygote deletion strains displayed better growth, haplo-proficiency, between the pool and batch stages. Interestingly, CUT248 deletion was haplo-proficient in all the conditions where nitrogen was limited (**Fig 1C; S10 Table**) which was confirmed in monoculture (**S1 Fig**). CUT248 is located near *DPS1* (**Fig 2A**) which is known to be up-regulated during yeast fermentation in the presence of diammonium phosphate [42]. Analysis of *DPS1* expression by quantitative real-time PCR (qRT-PCR) confirms that deletion of CUT248 induces an increase in *DPS1* expression in rich media and nitrogen-limiting conditions (**Fig 2A**). CUT248 therefore appears to repress *DPS1* transcription. Lowering the amount of CUT248 in a diploid background, allows increased *DPS1* expression, which could be beneficial for growth in nitrogen-limited conditions. In contrast to the deletion of CUT248, overexpression of the CUT248 RNA sequence from a plasmid in a wild-type haploid strain BY4741 results in a slow growth phenotype (**S2 Fig**) suggesting further that the levels of CUT248 are important for cellular fitness. A noticeable influence of temperature on fitness can be observed in the strain carrying the SUT340 deletion during the pool to batch transition (**Fig 1B and 1C**). SUT340 displays strong haplo-insufficiency at 36°C in both carbon-limited and nitrogen-limited conditions but not in any of the conditions at 30°C, revealing that this ncRNA with no known function is required for growth at high temperature. Additionally, RNA stabilization through inhibition of Xrn1 with LiCl triggers haplo-proficiency of CUT873 and tT(AGU)J specifically in carbon-limited conditions between the pool and batch stages (**Fig 1B; S10 Table**). We have also tested the deletion mutants tA(UGC)O, SUT340, CUT873 and tT(AGU)J in monoculture using the same conditions in which haplo-insufficient and haplo-proficient phenotypes were observed, and reconfirmed their phenotypes. The tA(UGC)O and SUT340 deletion mutant strains which were haplo-insufficient are both significantly less fit than the WT strain when grown in monoculture (**S3A Fig**). Similarly, the CUT873 and tT(AGU)J deletion mutant strains which were identified as being haplo-proficient are both significantly fitter than the WT strain when grown in monoculture (**S3B Fig**).

Deletion of the overlapping ncRNAs SUT233/CUT707 results in haplo-insufficiency in four of the six conditions in the pool to batch transition (**Fig 1B and 1C; S10 Table**) which was confirmed in monoculture (**S1 Fig**). SUT233 lies upstream of the gene *HAP4* which codes for a transcription factor involved in the diauxic shift in yeast [43–45]. CUT707 lies upstream of *KTI12* which codes for a protein that in yeast associates with the elongator complex required for tRNA modification [46,47]. *HAP4* expression is increased during the diauxic shift to allow the upregulation of the glyoxylate cycle with *HAP4* inducing the expression of approximately 88% of the proteins made during the diauxic shift [43,45]. Analysis of *HAP4* and *KTI12* expression by qRT-PCR confirms that in nitrogen-limiting conditions deletion of SUT233/CUT707 reduces expression of both *HAP4* and *KTI12* (**Fig 2B**). To show the number of haplo-insufficient and haplo-proficient ncRNA deletion strains in common between conditions in the pool to batch experiments UpSet diagrams of intersecting sets have been provided (**S4 Fig**).

**Fig 3:**
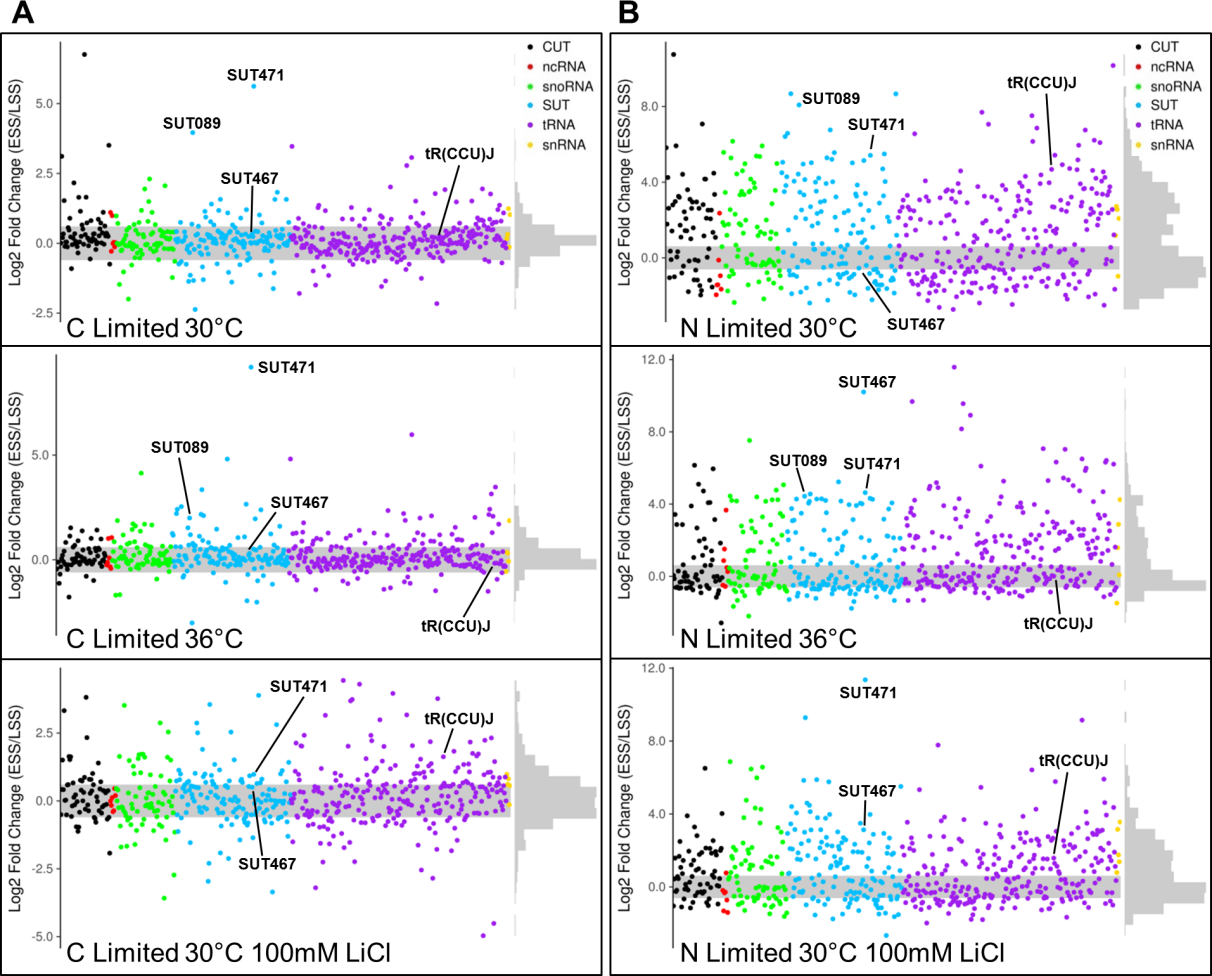
Analysis of early steady state to late steady state fitness changes. (**A**) Comparison of fitness between the early steady state (ESS) and late steady state (LSS) stages under the three indicated carbon-limited conditions. Haplo-proficient deletion strains have positive Log2 fold change and haplo-insufficient deletion strains have negative Log2 fold change. (**B**) Comparison of fitness between the early steady state (ESS) and late steady state (LSS) stages under the three indicated nitrogen-limited conditions. Haplo-proficient deletion strains have positive Log2 fold change and haplo-insufficient deletion strains have negative Log2 fold change. Any strains falling outside the grey shaded area have a significant fitness difference (p < 0.05). Graphs where individual points can be identified are found in **S4–S9 Tables**.

We next compared the fitness of the heterozygote ncRNA deletion strains between the early LSS and ESS stages (**Fig 3; S3–S9 and S11 Tables**). By keeping nutrients, pH and growth rate constant we were able to quantify smaller differences in fitness in response to changes in temperature. For example, deletion of SUT089 displayed haplo-proficiency in both carbon-limited and nitrogen-limited conditions at 30°C. However, this haplo-proficiency of SUT089 was significantly buffered in both carbon-limited and nitrogen-limited conditions at 36°C. Another striking example of temperature affecting the fitness of a heterozygous diploid ncRNA deletion strain is the large increase in fitness of SUT467 in nitrogen-limited conditions when temperature is increased from 30°C to 36°C. We have also found that SUT471 is haplo-proficient in all six conditions, therefore its presence clearly limits growth in continuous culture conditions. Under continuous culture conditions tR(CCU)J, which displayed severe haplo-insufficiency in the pool to batch growth phase, did not display any significant growth defect (**Fig 3A and 3B; S11 Table**). Therefore, analysis of deletion strains under continuous culture conditions clearly reveals additional phenotypes not seen in traditional batch culture where nutrients become limiting. To show the number of haplo-insufficient and haplo-proficient ncRNA deletion strains in common between conditions in the ESS to LSS experiments UpSet diagrams of intersecting sets have been provided (**S5 Fig**).

### Co-fitness analysis

**Fig 4:**
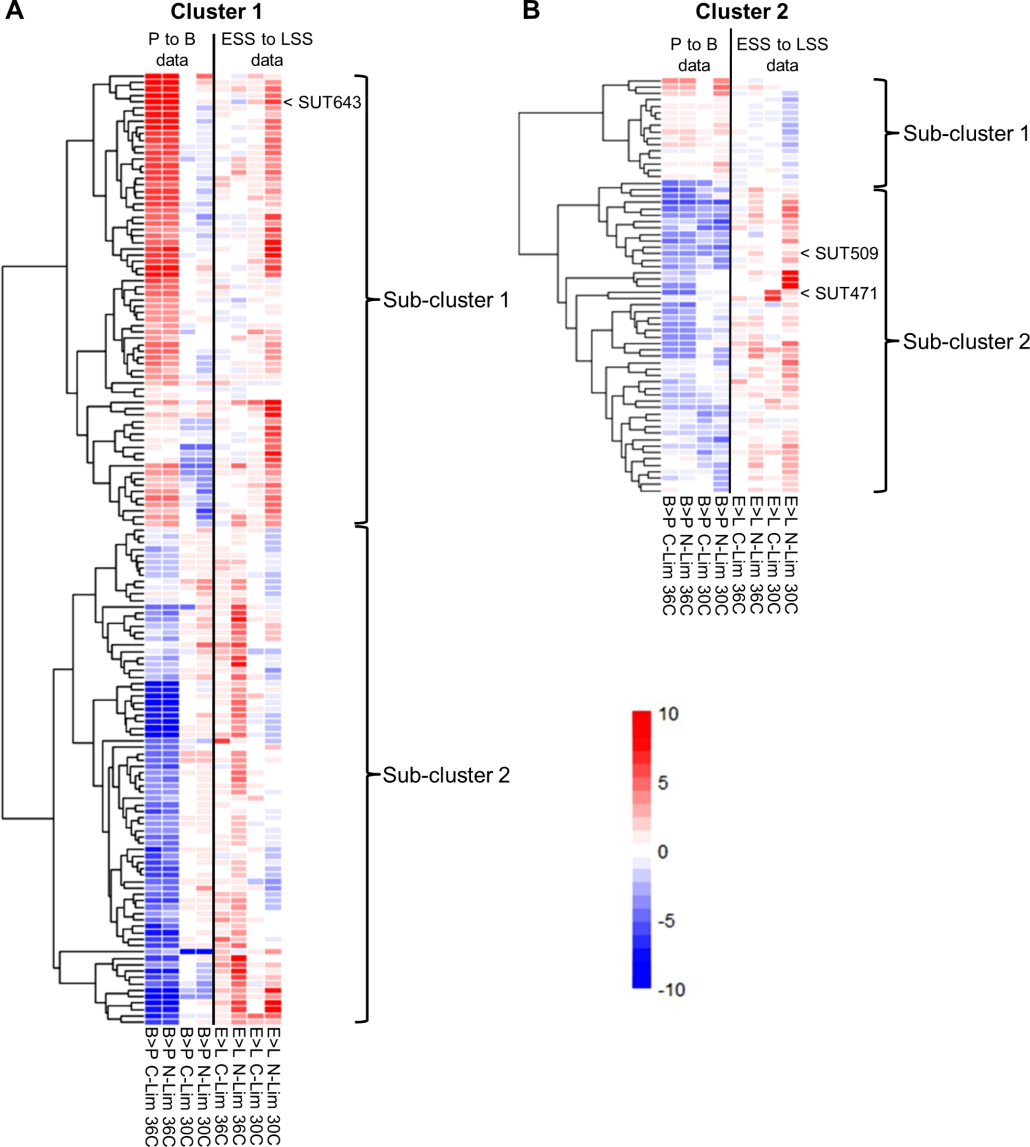
Co-fitness analysis. Variation of fitness profiles in Cluster 1 (**A**) and Cluster 2 (**B**). Rows represent individual ncRNA deletion strains. Columns represent the eight growth conditions analysed (B>P: comparison between batch and pool; L>E: comparison between late and early steady state; C-Lim: carbon-limited medium; N-Lim: nitrogen-limited medium). Colour bar represents Log2 fold change between batch and pool or late and early steady state. Haplo-insufficiency is shown in blue, and haplo-proficiency is shown in bright red. Data can be seen in **S12 and S13 Tables**.

Co-expression analysis has been used widely to infer functional relationships between protein encoding genes [48–51]. Here we apply a similar approach to our fitness data from eight different data sets to look for ncRNA deletion strains with similar fitness profiles and uncover phenotypic networks in the heterozygous ncRNA deletion collection. Four clusters were identified for a total of 226 deletion mutants which accounts for approximately 40% of the original dataset (**Fig 4; S12 and S13 Tables**). Our results indicate that deletion strains within each cluster followed the same fitness pattern throughout the eight testing conditions. Cluster 1 and 2 are the biggest containing 149 and 65 strains, respectively. Within these clusters the ncRNA deletion strains are separated into smaller sub-groups, sub-cluster 1 and sub-cluster 2, based on direction of fitness changes. The other clusters are relatively small (8 and 4 strains) and consist mainly of tRNAs and SUTs (**S13 Table**).

Cluster 1 encompasses strains with primarily specific response to temperature (**Fig 4A**). As shown in the heat map, this response to temperature is particularly evident in the initial pool (P) to batch (B) transition where, in any media considered, a change in fitness can be seen when the temperature is raised from 30°C to 36°C. Lowering the dosage of some ncRNAs either increases (**Fig 4A, sub-cluster 1**) or decreases (**Fig 4A, sub-cluster 2**) cell fitness with increasing temperature. The results suggest that ncRNAs in this cluster are involved in the optimal growth during heat stress and general nutrient deprivation. For example, SUT643 in sub-cluster 1 may have a function in transcriptional regulation of the neighbouring gene *IME1*, which is essential for meiosis, and is required for repression of *HSP82* [52–54]. Our data indicate that lowering the dosage of SUT643 has a positive impact on yeast growth at high temperature, suggesting that the repression on *HSP82* is partially lifted (the quantitative fitness profile for SUT643 is shown in **S6A Fig**).

Cluster 2 encompasses strains with specific response to growth phases, such as transition from P to B (batch phase with nutrient depletion) and from ESS and LSS stage (continuous culture phase with constant nutrients and pH), suggesting that ncRNA deletion strains in this cluster become important when nutrient levels are not constant (**Fig 4B**). In this case, lowering the dosage of some ncRNAs either increases (**Fig 4B**, **sub-cluster 1**) or decreases (**Fig 4B**, **sub-cluster 2**) cell fitness with nutrient depletions. When cells are about to reach stationary phase, there is a decline in overall transcriptional activities and several changes in cellular metabolism occur to store complex carbohydrates such as glycogen and trehalose [55–57]. Based on our data, sub-cluster 2 (**Fig 4B**) encompasses ncRNAs which are crucial for survival during the pool to batch stage. We found that some ncRNAs in this sub-cluster 2 are located next to genes that are highly correlated with transition to stationary phase. For example, SUT471 is located downstream of *SNF11* and upstream of *TPS2*. *TPS2* encodes for a phosphatase in the last step of the trehalose pathway, important for carbon storage and is activated sequentially after diauxic shift and is suppressed fully before entering stationary phase [58,59]. *SNF11* encodes for a subunit of the SWI/SNF chromatin remodelling complex, which is involved in transcriptional regulation of several genes at the onset of stationary phase [60,61]. Another example is SUT509 which is located downstream of the medium chain fatty acyl-CoA synthetase gene *FAA2* which has a transcriptional profile similar to that of the gene *TPS2*. The effect of SUT471 and SUT509 deletion on the neighbouring genes may, therefore, be responsible for their haplo-insufficiency in the pool to batch transition (the quantitative fitness profiles for SUT471 and SUT509 are shown in **S6B and S6C Fig**). We further analyzed all four clusters for representation of different ncRNA classes and found no bias in the distribution of SUTs or CUTs (FDR > 0.05). In addition, we could not identify any common biological functions using the gene ontology terms of neighbouring genes for the ncRNAs that comprise the different clusters.

### Phenotypic screening of the haploid ncRNA deletion collection

**Fig 5:**
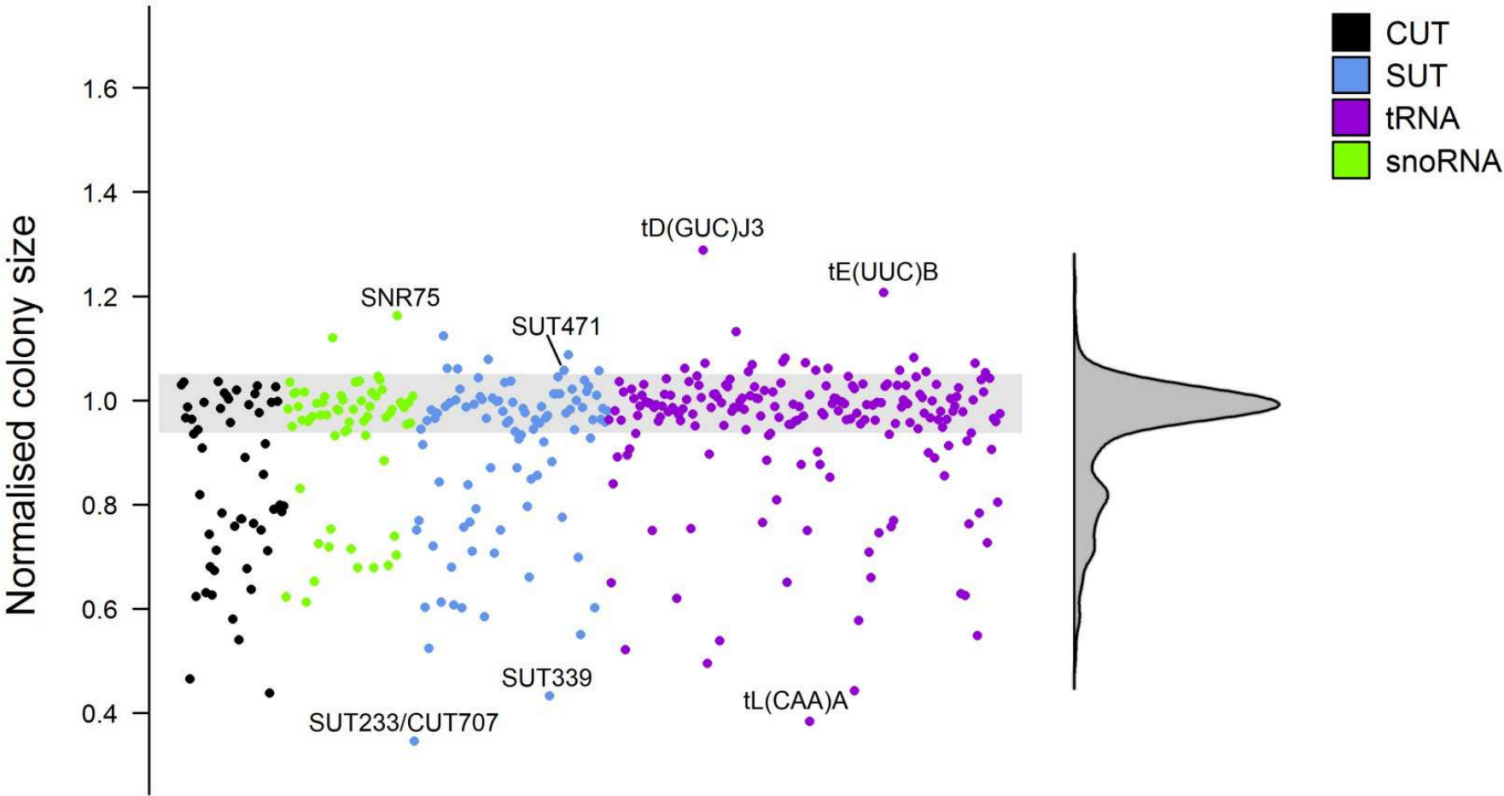
Haploid deletion strain phenotypic screen. Scatter plot of the normalized colony size values for each of the haploid ncRNA deletion strains growing on YPD plates at 30°C. Any strains falling outside the grey shaded area are significantly different than the wild-type strain (p < 0.05). Data can be seen in **S14 and S15 Tables**.

To determine the influence of complete removal of a ncRNA on cell fitness we individually arrayed each strain of the haploid deletion collections on rich media (YPD) plates at 30°C and assessed colony size compared to the wild-type strain. The haploid deletion collections exhibited significant variation in fitness on YPD and this variation was detected across all types of ncRNA (**Fig 5; S14 and S15 Tables**). The deletion overlapping both SUT233 and CUT707 (**Fig 2B**), which displayed significant haplo-insufficiency as a diploid heterozygotic deletion in most conditions in the pool to batch growth (**Fig 1**), is the least fit in the haploid deletion collection (**Fig 5**). Deletion mutants of tL(CAA)A and SUT339 are respectively, the second and third least fit strains in the haploid collection on YPD media. The tL(CAA)A tRNA is part of a family of tRNAs for the leucine CAA codon and deletion of tL(CAA)A has previously been shown to significantly impair growth on YPD, whereas other members of this tRNA family do not display a severe fitness defect [62] (**S14 Table**). Our data support the idea that there are major and minor copies in tRNA gene families and loss of different members of a tRNA family affect cellular fitness differently [62]. From the genomic location of SUT339 there is no immediately obvious reason how its deletion is affecting fitness. The SNR75, tD(GUC)J3 and tE(UUC)B deletion strains are the top three fittest strains in this plate assay showing increased growth. SUT471 deletion also had a significantly positive effect on fitness in the haploid background (**Fig 5**). This positive effect on fitness is consistent with SUT471 being haplo-proficient in all of the continuous culture conditions (**Fig 3**), supporting the theory that SUT471 expression limits cell growth. ncRNA deletions showing little fitness change in the heterozygote background, but significant effects in the haploid background, have also been identified here (**S15 Table**). These data demonstrate that useful fitness data can be obtained from the plate array method of phenotyping on solid media. Moreover, some of the most dramatic phenotypes which were scored via colony size (**Fig 5**) are also seen in our continuous culture experiments (**Figs 1 and 3**). For example, the SUT004, SUT107, CUT356, SNR10 and tQ(UUG)L deletion mutants which were haplo-insufficient in at least one of the continuous culture conditions, also displayed significantly impaired fitness in the haploid fitness screen. Expanding this array method to a variety of other growth conditions should further our understanding of ncRNA function.

### Identification and analysis of essential ncRNAs

**Fig 6:**
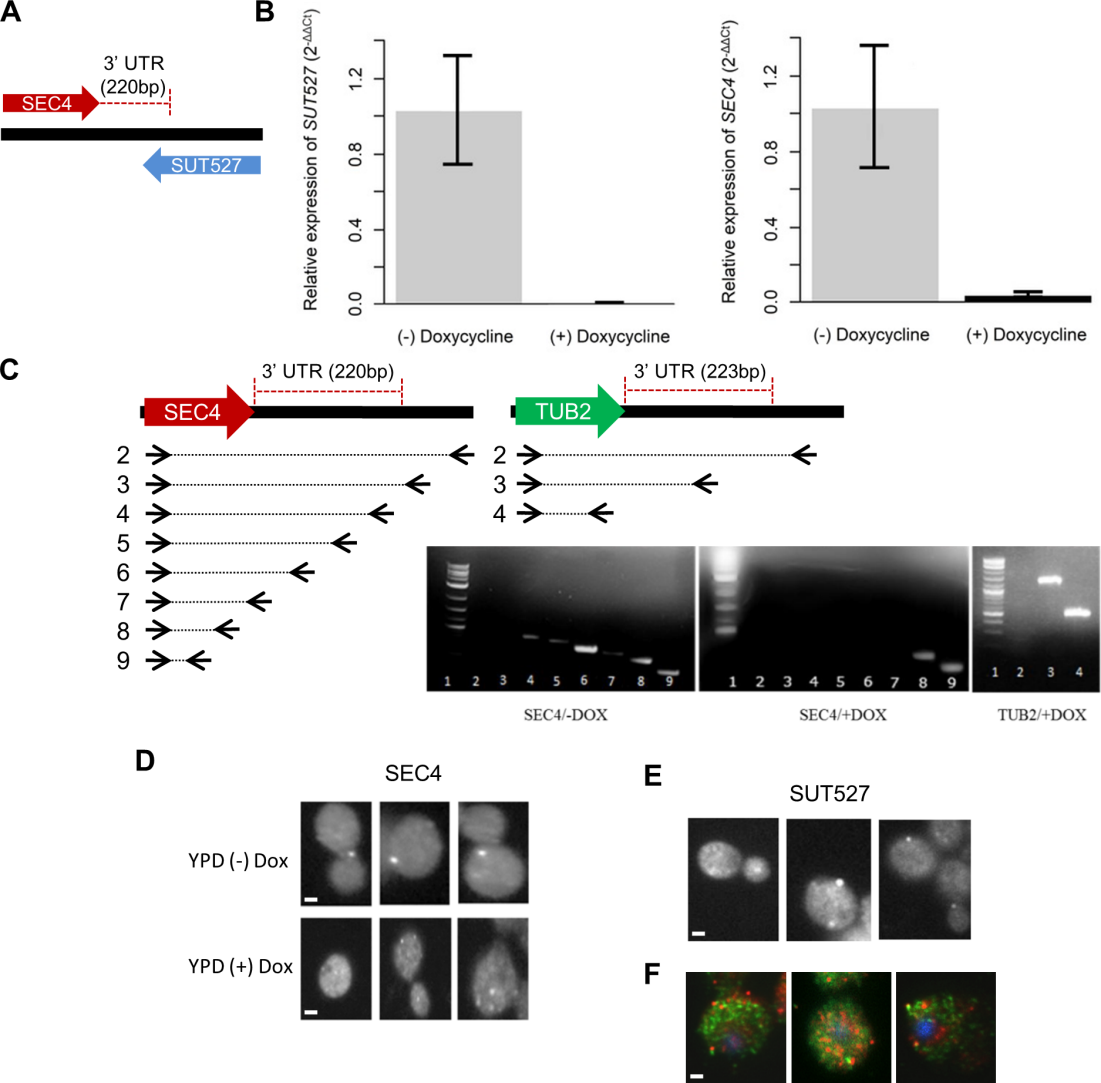
Analysis of an essential ncRNA. (**A**) Overlap of the ncRNA SUT527 with the 3’ UTR of *SEC4*. (**B**) qRT-PCR analysis of SUT527 and *SEC4* RNA levels in a strain with SUT527 under control of the tetO7 element. Grey bars represent the relative expression of SUT527 and *SEC4* in YPD media (-) Doxycycline. Black bars represent the relative expression of *SUT527* and *SEC4* in YPD (+) Doxycycline. Error bars (SD) are from three technical replicates from three independent biological replicates. Relative normalized expression was calculated using *ACT1*. P values were calculated using the Welch two sample t-test. SUT527: *p* = 0.02, *SEC4*: *p* = 0.03. (**C**) Primer walking of cDNA isolated from cells expressing (-DOX) or not expressing (+DOX) SUT527 to assess 3’ UTR formation. Top panels depict the locations and number of the different back primers used with a common forward primer for the *SEC4* and *TUB2* RNAs. (**D**) *SEC4* mRNA was localized by FISH in the presence and absence of SUT527 expression. When SUT527 was expressed in YPD (-) DOX, 32% of the *SEC4* mRNA was localized to the cell membrane. The absence of SUT527 expression in YPD (+) DOX decreased localisation of *SEC4* mRNA to 13%. (**E**) SUT527 localization was determined by FISH. Under normal growth with YPD 33% of SUT527 was observed in foci at the cell surface similar to the localization of *SEC4*. (**F**) Three representative images of SEC4 (red) and SUT527 (green) localized together in the same cells. Nuclei are stained with DAPI (blue). Scale bars, 1μm.

The heterozygote ncRNA deletion strains were induced to sporulate and the haploid spores dissected to reveal whether individual ncRNA gene deletion was essential for growth. Three percent of the ncRNA gene deletions (17 of 532) were found to be essential in nutrient rich conditions (YPD), with thirteen of these (*i*.*e*. snRNAs, snoRNAs, tRNAs) already known to be essential (**S2 Table**). Four novel essential ncRNAs were identified, SUT075, SUT367, SUT527 and SUT259/691, and were found to be essential in separate biological replicates of the deletion strains (**S7 Fig**). One of these essential ncRNAs, SUT527 (also annotated as *RUF20*), overlaps by 140 base pairs with the 3’ untranslated region (UTR) of the essential gene *SEC4*, a GTPase required for vesicle-mediated exocytic secretion and autophagy [63,64] (**Fig 6A**). To determine whether SUT527 essentiality was derived from its overlap with the 3’ UTR of the essential *SEC4*, two shorter deletions of SUT527 were constructed with 40bp overlap and no overlap with the *SEC4* 3’ UTR. The shorter SUT527 deletion, that still overlapped the *SEC4* 3’ UTR, resulted in a non-viable phenotype, whereas the strain containing a SUT527 deletion with no overlap with the *SEC4* 3’ UTR was viable (**S8 Fig**). This viability indicates that SUT527 essentiality is derived from the overlap with the 3’ UTR of the essential gene *SEC4* and that deletion in this region does not generally cause silencing of *SEC4* transcription. Transformation of the original SUT527 diploid deletion strain with a plasmid containing an approximately 1.4kb DNA fragment containing the *SEC4* sequence known to complement *SEC4* function [64] restored strain viability after sporulation and tetrad dissection. To understand whether the essential phenotype was caused by the deletion of the *SEC4* 3’ UTR in itself or caused by the interaction of the SUT527 RNA with the *SEC4* 3’ UTR, we reduced SUT527 expression in a haploid strain using a regulated Tet promoter [65]. We found that *SEC4* mRNA expression was greatly decreased (**Fig 6B**) and *SEC4* 3’ UTR formation was affected when SUT527 expression was suppressed (**Fig 6C**). The *SEC4* 3’ UTR is required for localization of *SEC4* mRNA [66]. Fluorescent *in situ* hybridization (FISH) revealed that SUT527 displayed a similar punctate localization to *SEC4* mRNA and *SEC4* mRNA was mislocalized when SUT527 expression was switched off (**Fig 6D and 6E**). Analysis of data sets from a global sequence analysis of small RNAs from *S*. *cerevisiae* strains engineered for RNAi to reveal the presence of dsRNAs [21], identified small RNAs produced from SUT527 in the region of overlap with the *SEC4* 3’ UTR (**S9 Fig**). The presence of these small RNAs in the region of overlap between *SEC4* and SUT527 indicates that *in vivo* there is dsRNA formation between SUT527 and the *SEC4* 3’ UTR. In fact, FISH of *SEC4* mRNA and SUT527 in the same cells with different coloured detection probes revealed that in cells approximately 10% of the *SEC4* mRNA (red) and SUT527 RNA (green) puncta were found next to each other (**Fig 6F**). We have therefore defined a molecular function for the ncRNA SUT527 and suggest that the physical interaction between SUT527 and the 3’ UTR of *SEC4* influences *SEC4* 3’ end formation and mRNA localization.

**Fig 7:**
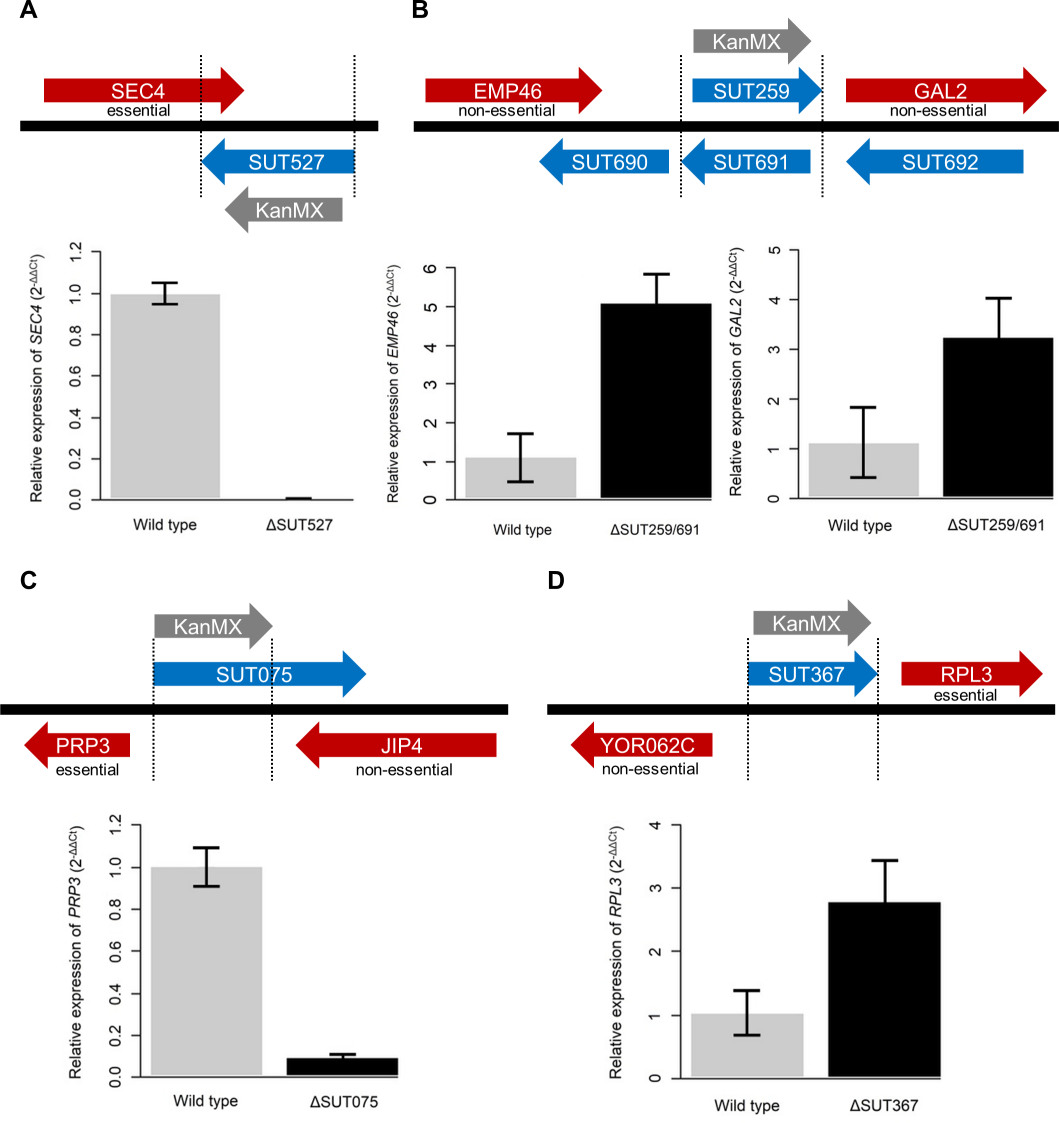
Genome locations of the essential ncRNAs and the expression of nearby genes. The ncRNA deletions are indicated by vertical black dotted lines and the direction of KanMX deletion cassette expression is indicated with a grey arrow. Red arrows are protein coding genes and the arrows encompass the annotated 5’ and 3’ UTR regions. The essentiality of nearby protein coding genes is indicated. (**A**) SUT527 full deletion. Relative expression of *SEC4* in the wild type diploid background is represented by grey bar and *SEC4* expression in the SUT527 diploid deletion background is represented by black bar. *SEC4*: *p* = 0.02. (**B**) Overlapping SUT259/691 deletion. Relative expression of *EMP46* and *GAL2* in the wild type diploid background is represented by grey bars and *EMP46* and *GAL2* expression in the SUT259/691 diploid deletion background is represented by black bars. *EMP46*: p = 0.02, *GAL2*: p = 0.02. (**C**) SUT075 partial deletion. Relative expression of *PRP3* in the wild type diploid background is represented by grey bar and *PRP3* expression in the SUT075 diploid deletion background is represented by black bar. *PRP3*: *p* = 0.04. (**D**) SUT367 full deletion. Relative expression of *RPL3* in the wild type diploid background is represented by grey bar and *RPL3* expression in the SUT367 diploid deletion background is represented by black bar. *RPL3*: *p* = 0.02. The fold change (2^) in expression, relative to the wild-type was calculated using the ΔΔCт method and *ACT1* as a reference gene. Error bars were calculated using three independent biological samples. P values were calculated using the Welch two sample t-test.

**Fig 8:**
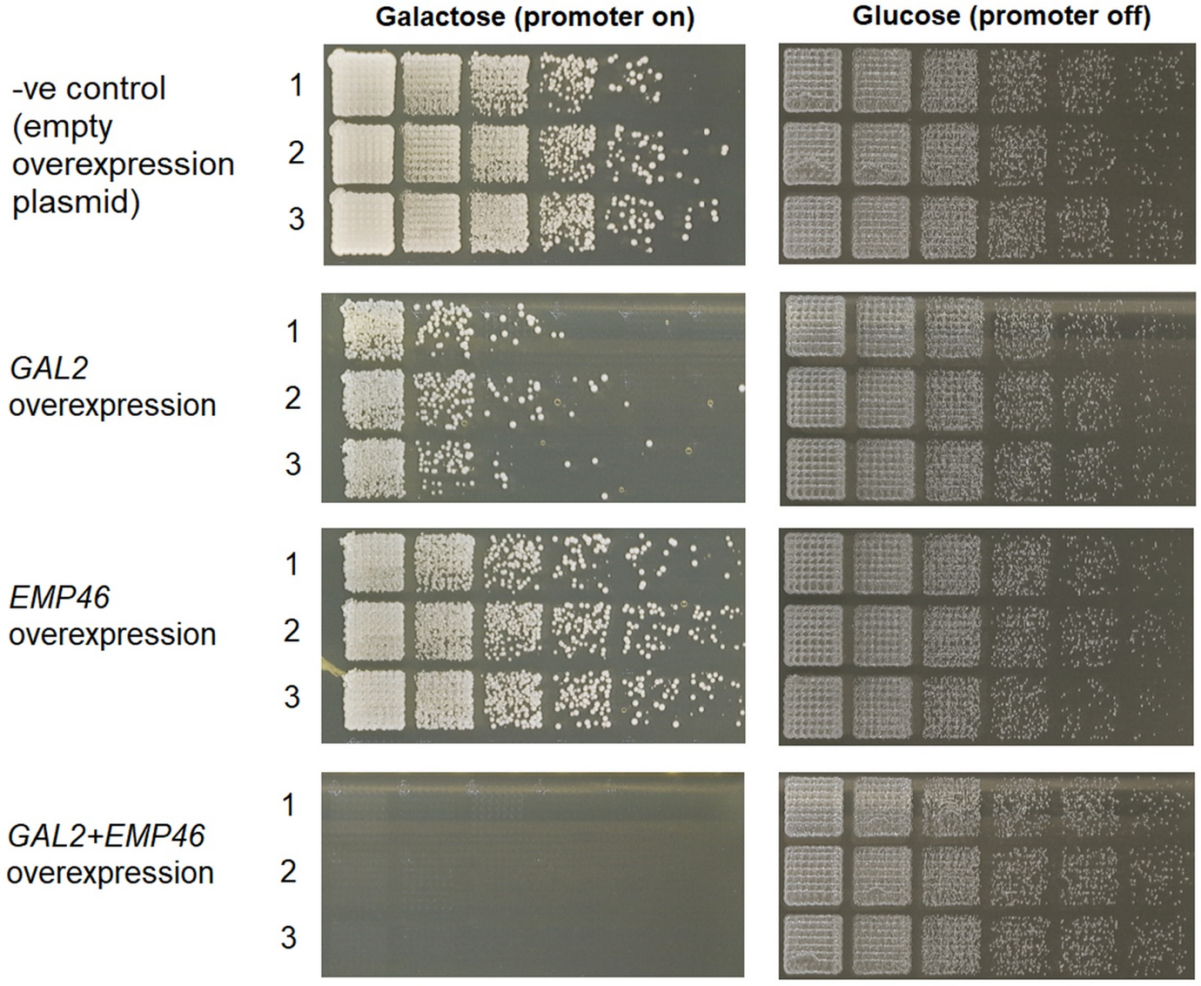
Spot assays for strains containing *EMP46*, *GAL2*, *EMP46/GAL2* or empty pBEVY-GA overexpression plasmids. The comparative fitness of the identified strain when grown on galactose media or glucose media, which activates and inactivates the GAL1/10 promoter, respectively.

Of the four essential ncRNAs identified here (SUT527, SUT075, SUT367 and SUT259/691) SUT527, SUT075 and SUT367 are located adjacent to essential genes. Deletion of the ncRNA with the *KanMX* cassette could potentially remove essential regulatory elements for a nearby essential gene, or the expression from the *KanMX* module may influence the expression of a nearby essential gene. To determine the influence of deleting a single copy of SUT527, SUT075, SUT367 or SUT259/691 on the expression of nearby genes, qRT-PCR was used to analyze expression of nearby genes in the diploid deletion strains compared to the wild-type diploid strain. Analysis of the diploid SUT527 deletion strain revealed greatly reduced expression of *SEC4* as expected (**Fig 7A**). Deletion of the overlapping SUT259/691 ncRNAs increased the expression of the upstream and downstream non-essential genes *EMP46* and *GAL2* which are both transcribed in the same direction as the *KanMX* (**Fig 7B**). SUT690 is located between *EMP46* and SUT259/691. It is plausible that SUT690 might be the target of SUT259/691 regulation. However, analysis of SUT690 expression in the ΔSUT259/691 strain reveals that SUT690 expression levels are unchanged (**S10 Fig**). The deletion of either *EMP46* or *GAL2* alone does not result in a lethal phenotype, but since a double deletion mutant of *EMP46* and *GAL2* shows positive epistasis [67], it is possible that overexpression of both genes gives the opposite effect and hampers fitness (see Discussion). To test this hypothesis, we have cloned both *EMP46* and *GAL2* into the pBEVY-GA plasmid, containing a bi-directional GAL1/10 promoter. Overexpression plasmids with *EMP46*, *GAL2* or both *EMP46* and *GAL2* were created and transformed into the BY4743 background strain. The comparative fitness of these overexpression strains were then examined using spot assays. Solitary overexpression of *GAL2* or *EMP46* was not lethal, however they resulted in impaired fitness, particularly the overexpression of *GAL2* (**Fig 8**). The simultaneous overexpression of *GAL2* and *EMP46* resulted in no cell growth and is therefore lethal (**Fig 8**). This lack of growth supports the idea that the lethality, observed in the SUT259/691 knockout strain, is a result of a combined increase in *EMP46* and *GAL2* expression. The partial deletion of SUT075 caused a large decrease in the expression of the essential gene *PRP3* which is transcribed in the opposite direction to SUT075 and the *KanMX* expression (**Fig 7C**). The decreased expression of the essential *PRP3* may be the explanation for SUT075 lethality. Deletion of SUT367 caused an increase in the expression of the essential gene *RPL3* which is transcribed in the same direction downstream of SUT367 (**Fig 7D**). Interestingly, *RPL3* is one of the few ribosomal protein genes in yeast that is neither duplicated nor contains an intron, both properties that are associated with increased ribosomal protein gene expression [68]. Therefore, increased expression of *RPL3* may be detrimental to cells providing a reason for SUT367 lethality (see Discussion). Overall, we observed that deletion of ncRNAs can both positively and negatively influence the expression of nearby genes and that in some cases can explain the lethality.

**Fig 9:**
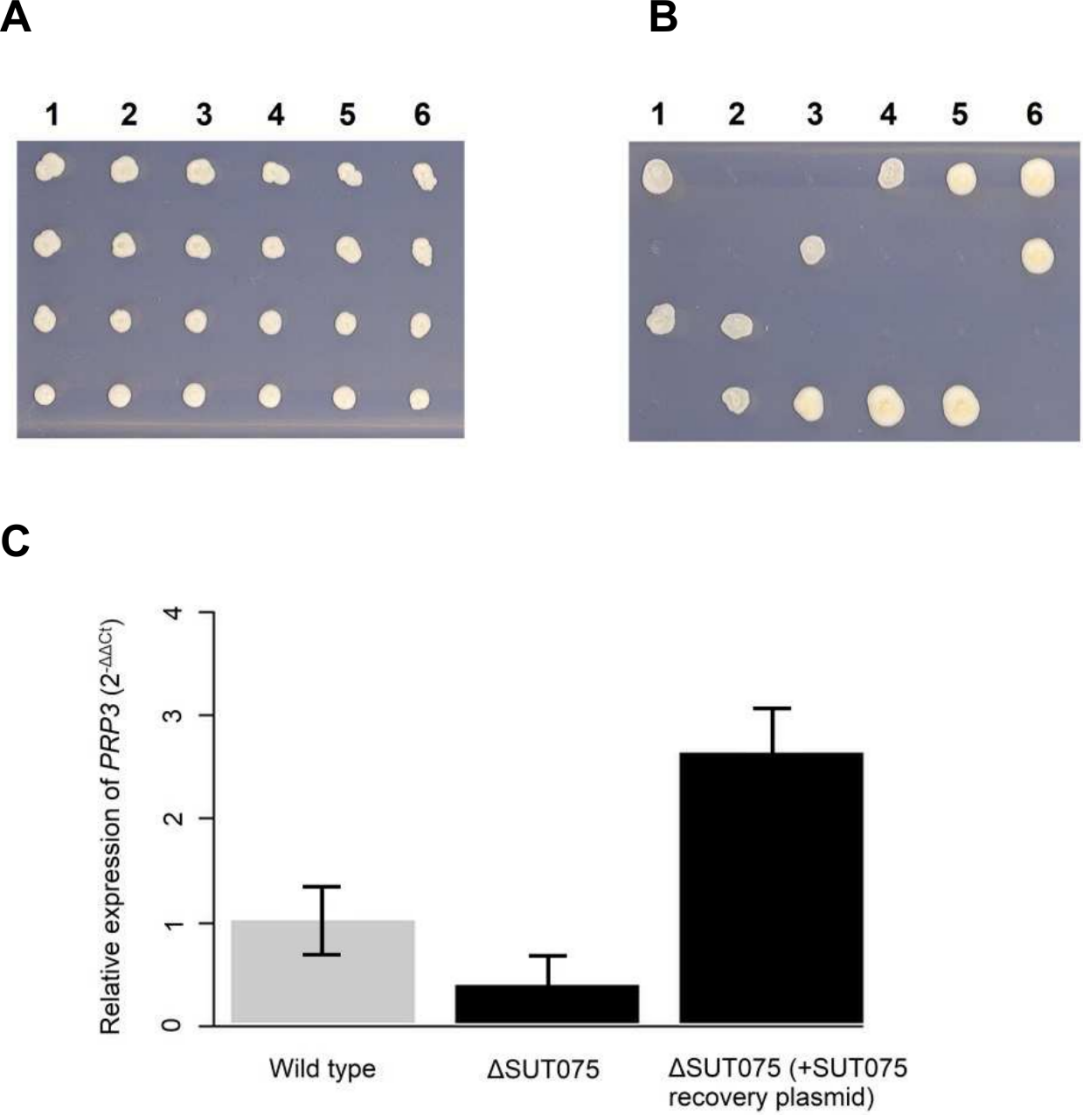
Expression of SUT075 in *trans* rescues the lethal phenotype of a SUT075 deletion and increases *PRP3* expression. (**A**) Haploid spores from dissection of six *MAT*a*/α* SUT075*Δ*/SUT075 diploid tetrads were spotted on SD media lacking uracil and containing 2% galactose. The plasmid expressing the SUT075 ncRNA is selected for using the *URA* auxotrophic marker. Galactose induces expression of SUT075 present in the plasmid. (**B**) Haploid spores from dissection of six *MAT*a*/α* SUT075*Δ*/SUT075 diploid tetrads were spotted on SD media lacking uracil containing 2% galactose and 300mg/L G418 disulphate. G418 resistance selects for haploids deleted for SUT075. Spores growing in both panels A and B are considered to contain the G418 resistance SUT075 deletion cassette and the SUT075 ncRNA expressing plasmid. (**C**) Expression levels of *PRP3* in the ΔSUT075 and the ΔSUT075 (+ sense SUT075 recovery plasmid) heterozygote diploid strains measured by qRT-PCR. The relative expression of *PRP3* in the wild-type background is represented by a grey bar and a black bar in the ncRNA deletion strain backgrounds. Using the ΔΔCт method and *ACT1* as a reference gene, the fold change (2^) in expression, relative to the wild-type was calculated. Error bars are calculated using each of the three independent biological samples. P values calculated using the Welch two sample t-test determine there to be a significant difference (*p* = 0.02) between the ΔSUT075 and the ΔSUT075 (+ sense SUT075 recovery plasmid) strains.

To investigate further the essentiality of ncRNAs SUT527, SUT075, SUT367 and SUT259/691, we overexpressed these SUTs from plasmids to discover any that could recover the lethal phenotype of the corresponding deletion strain and identify *trans* ncRNA effects. We constructed centromeric plasmids with each SUT expressed in either the sense or antisense orientation from the *GAL1* promoter. These plasmids were then transformed into the corresponding heterozygote diploid deletion strains. These strains were then sporulated and tetrads dissected. Successful generation of viable haploid strains, containing the deleted essential SUT, would indicate the ability of the overexpressed SUT to function in *trans*. The SUT367, SUT527 and SUT259/691 sense or antisense plasmids were unable to reverse lethality of the corresponding ncRNA deletion in the haploid background following sporulation and tetrad dissection. However, deletion of SUT075 was no longer lethal when the sense orientation SUT075 plasmid was present (**Fig 9A and 9B**) but was still lethal with the antisense SUT075. This complementation of the SUT075 deletion strain lethal phenotype by the ectopic expression of SUT075 RNA indicates that SUT075 functions in *trans*. It is, therefore, plausible that SUT075 regulates expression of distal genes in the genome, not just the neighbouring *PRP3* gene. The deletion of one copy of SUT075 in the diploid background (**Fig 7C**) significantly reduced expression of the adjacent *PRP3* gene. Expression of *PRP3* was measured in the heterozygote diploid SUT075 deletion strain with the sense SUT075 plasmid expressing the SUT075 ncRNA, to determine if the rescue of the lethal phenotype in the haploid progeny (**Fig 9A and 9B**) was the result of *PRP3* expression levels being returned to normal. *PRP3* expression was found to be 8.3 fold greater in the heterozygote diploid SUT075 deletion strain, when the SUT075 expression plasmid was present (**Fig 9C**). Recovery of *PRP3* expression, to levels greater than in wild type cells, suggests that the *GAL1* promoter is stronger than the native SUT075 promoter. Overall, these data confirm that expression of SUT075 from a plasmid is able to up-regulate *PRP3* expression in *trans* and reverse lethality in strains deleted for SUT075.

### The yeast ncRNA analysis database

To ease the use and access of our extensive functional fitness data for future research, we have built a publicly accessible online resource called the Yeast ncRNA Analysis (YNCA) (http://sgjlab.org/ynca/) to host the heterozygote and haploid deletion fitness profiles for each of the deleted ncRNAs. Data are searchable by neighbouring genomic features, ncRNA type, essentiality, chromosomal position and growth phenotype for each growth medium used, as well as searchable by ncRNA name as classified in Xu *et al*. (2009) [9]. The types of searchable neighbouring genomic features are known open reading frames, tRNA genes, snoRNA genes, centromeric and telomeric regions, autonomous replicating sequences (ARS), long terminal repeats (LTR), pseudogenes, LTR retrotransposons and transposon internal genes. The user can download both raw experimental values and statistical significance values from a results table specific to the search performed. The list of barcode TAGs associated with each strain is also available on the website. We plan to progressively expand the YNCA database to include homozygote deletion strain fitness data under a variety of conditions and results from future analyses.

## Discussion

By utilizing the newly developed ncRNA deletion strain collections in the yeast *Saccharomyces cerevisiae* we have carried out large scale profiling of ncRNA function under a variety of growth conditions and phases. The extensive functional fitness data can be accessed via the database YNCA (http://sgjlab.org/ynca/) where the influence of individual ncRNA deletion strains on cellular fitness has been catalogued in an easy to navigate and searchable website. This large scale functional profiling has now provided valuable functional information on the deletion of 532 different ncRNAs which includes tRNAs, snoRNAs, snRNAs, SUTs, CUTs and various other annotated ncRNAs. We have also investigated in more detail four novel essential ncRNAs and determined the mechanisms by which they result in a lethal phenotype when deleted.

Yeast strains deleted for individual tRNA genes have been previously constructed with these deletion strains tested for both growth rate and growth yield under a number of conditions [62]. While there is significant overlap between our collection and that of Bloom-Ackermann et. al., there are strains that are unique to each collection (**S16 Table**). Where there is overlap between collections we have observed similar growth phenotypes of tRNA deletion strains. For example, our observation that the tR(CCU)J deletion strain displays decreased fitness in all the conditions we tested during the pool to batch growth phase was also observed for the growth rate of the tR(CCU)J deletion strain in four of the six growth conditions used by Bloom-Ackermann et al [62]. Within tRNA families major and minor tRNAs have been identified where deletion of the major tRNA influences the ability of a deletion strain to grow under different conditions more than one of the minor tRNAs in the same family [62]. For instance, the tRNAs tR(UCU)E and tR(UCU)M2 were identified as being major tRNAs that are influenced the most by different growth conditions in their family [62]. In the six conditions tested here we have also found that the tR(UCU)E deletion displays decreased fitness in all six conditions in the pool to batch transition. However, we have identified tR(UCU)B in the same family, a deletion novel to our collection, that also displays decreased fitness in all six conditions in the pool to batch transition and tR(UCU)G1 as less fit in four of the six conditions in the pool to batch transition (**S4–S10 Tables**). In contrast, we did not observe a significant decrease in fitness with deletion of tR(UCU)M2 in any of the six conditions in the pool to batch transition. Analysis of the tRNA deletion strains in the tR(UCU) family under continuous growth conditions did not identify any tRNAs in the tR(UCU) family that displayed a consistently significant decrease in fitness, when deleted, in any of the conditions we tested. These results suggest that tRNA levels are more important in conditions where nutrients become limited.

By using continuous growth conditions, we have uncovered additional phenotypes for ncRNA deletion strains that are not observed under growth conditions where nutrients become limiting. Specifically, we have observed changes in fitness associated with temperature changes that were not observed in the pool to batch growth phase. Additionally, phenotypes observed in the pool to batch stage where nutrients are limited were not observed in continuous culture. By observing the fitness of the deletions strains by two distinct methods of cell culture we have produced an extensive catalog of fitness data for each of the heterozygous diploid deletion strains. Combined with our analysis of the haploid deletion collections arrayed on solid media, overall we present the most extensive analysis of ncRNA requirements for cellular fitness to date. These data have been compiled into a database called YNCA. YNCA uses the more sensitive ESS-LSS fitness change for search based on the heterozygote fitness profile, but displays both pool-batch and ESS-LSS fold change values in the detailed ncRNA-specific page. Both sets of data are downloadable. For each growth medium, the user can retrieve strains which display any or one selected fitness phenotype (after statistical analysis; gain/loss of fitness or haplo-proficient/insufficient) or obtain a list of all strains with available experimental data, for raw data download. Given that only a few examples of CUTs and SUTs have a known function and that the analysis of ncRNA function sometimes focuses on the regulation of neighbouring genes, the YNCA website offers the option to search by nearby genomic feature, hence facilitating the selection of candidate ncRNAs as gene-specific or feature-specific regulators.

In the construction and analysis of the ncRNA deletion strains we have identified ncRNAs that are essential for cell growth. Many of these essential ncRNAs have been previously identified and annotated as essential ncRNAs. The five snRNAs (U1, U2, U4, U5 and U6) required for pre-mRNA splicing, the snoRNAs snR128 (U14) and snR30 (U17), the RNA component of RNase MRP (NME1), the RNA component of nuclear RNase P (RPR1) and tRNAs tR(CCG)L, tR(CCU)J, tS(CGA)C and tT(CGU)K have all been previously shown to be essential. Besides the known essential ncRNAs we have identified four novel ncRNAs that are essential when deleted. These four novel essential ncRNAs are SUT075, SUT367, SUT527 and SUT259/691. The essentiality of SUT527 is caused by its overlap with the 3’ UTR of the essential protein coding gene *SEC4*, as making smaller deletions that did not overlap the annotated 3’ UTR of *SEC4* did not result in a lethal phenotype. It appears that the overlap of SUT527 with the 3’ UTR of *SEC4* is required for both the stability of the *SEC4* mRNA and for the localization of SEC4 mRNA. *SEC4* mRNA localization is determined by its 3’ UTR [66]. There is evidence that *SEC4* 3’ UTR/SUT527 RNA duplexes are formed within cells [21] and we have observed that the *SEC4* mRNA and SUT527 RNA localize in close proximity. A cytoplasmic function for other SUTs is very likely as a proportion of SUTs are transported to the cytoplasm where they have been proposed to exert their function [31].

The ncRNA SUT367 was found to be essential when deleted, but analysis of the nearby essential gene *RPL3* in the diploid heterozygous deletion strain revealed that *RPL3* expression is increased (**Fig 7D**). Large scale screens have previously identified that overexpression of *RPL3* causes growth impairment, disrupts the cell cycle [69] and induces chromosome instability [70]. The mechanisms by which deletion of SUT367 leads to *RPL3* overexpression or how overexpression of a ribosomal protein gene leads to chromosome instability/cell cycle disruption and lethality is not clear. However, other ribosomal protein genes have also been identified to cause chromosome instability/cell cycle disruption leading to cell lethality when overexpressed [69,70]. We show that deletion of SUT367 prevents spores from germinating after meiosis, and it is plausible that the resulting overexpression of *RPL3* is responsible for the inability of the spores to grow.

A deletion of SUT259/691 is lethal and this deletion results in the overexpression of two adjacent nonessential protein coding genes *EMP46* and *GAL2*, but not the adjacent SUT690, in the diploid heterozygote (**Fig 7B, S10 Fig**). Individual overexpression of either *EMP46* or *GAL2* displays a slow growth phenotype on their own ([71] and **Fig 8**of this a manuscript). When *EMP46* and *GAL2* are overexpressed simultaneously the cells are unable to grow (**Fig 8**). Therefore, SUT259/691 are essential for the regulation of *EMP46* and *GAL2*, and when deleted cause an overexpression of these genes which causes lethality.

The SUT075 is expressed in the opposite direction to the essential gene *PRP3* with the deletion we made of SUT075 being 230 nucleotides away from the start of *PRP3* and 143 nucleotides away from the stop codon of the non-essential gene *JIP4* (**Fig 7C**). We successfully used complementation to determine that expressing the full length SUT075 RNA from a plasmid in *trans* could rescue the essential phenotype of SUT075 deletion. Therefore, we have identified another example of a ncRNA that works in *trans*. The action of SUT075 is in part locally as the *trans* expression of SUT075 increases the expression of the adjacent essential gene *PRP3*, but there is also the possibility that SUT075 acts elsewhere in the cell. As transcription of SUT075 produces an RNA that works in *trans* we investigated yeast ribosome profiling data and found that SUT075 does not associate with ribosomes so is unlikely to be translated into protein [34]. To date only a few examples of ncRNAs working in *trans* have been identified. The Ty1 RTL CUT ncRNA has been found to regulate Ty1 expression in *trans* [72] and the ncRNA PHO84 can work in *trans* to silence genes [73]. Recent work has found two new *trans* acting SUTs suggesting that *trans* acting ncRNAs may be more prevalent than previously thought [30]. Our identification of SUT075 as another *trans* acting ncRNA supports the view that there may be more *trans* acting ncRNAs identified in the future. Overall, analysis of these ncRNAs, initially identified as being essential, has revealed that the transcription of ncRNA can both positively and negatively influence the expression of adjacent genes or produce an RNA that can function on its own, indicating that regions of the genome identified as producing SUTs and CUTs are functional and do not represent just transcriptional noise.

By exploiting the yeast ncRNA deletion collections we produced a large array of phenotypic data which is a useful resource for providing a snapshot of ncRNA function in the cell. By expanding the number of conditions investigated it is hoped that a picture can be built of how ncRNAs contribute to the fitness of cells in different environments. Here by exploring individual examples of ncRNA we have determined the molecular function of SUT527 and also showed that SUT075 works in *trans*, expanding the repertoire of cellular functions that require ncRNAs. As the characterization of the numerous ncRNAs continues, the use of ncRNA deletion collections in large-scale functional and interaction studies will ultimately provide information on how ncRNAs fit into the functional framework of the cell.

## Methods

### Strains and primers

All *S*. *cerevisiae* strains and primers used are listed in **S17 Table and S18 Table**. Individual deletion strains or the collection of deletions strains are available on request.

### Deletion strain and heterozygous deletion strain pool construction

Methods for the construction of the deletion strain collections have been previously described [35]. In preparation for chemostat continuous culturing of the heterozygote collection, a pool of the deletion strains was prepared. A -80°C stock of heterozygous ncRNA deletion strains were grown overnight at 30°C in liquid YPD and the OD_600_ of each strain in the microtitre plate was read using a FLUOstar OPTIMA plate reader (BMG Labtech). Subsequently each strain was normalised to an OD_600_ of 0.1 and pooled.

### Competition experiments in continuous (chemostat) culture

The diploid heterozygote ncRNA deletion collection pool was grown in chemically defined F1 medium limited for glucose (carbon limitation) or nitrogen at 30°C and 36°C. The pooled heterozygous deletion strains were also grown in F1 medium limited for glucose or nitrogen at 30°C in the presence of 100mM LiCl. The diploid heterozygote ncRNA deletion collection pool was grown in batch culture for 24hrs and then switched to continuous culture where it took about 42 hrs to reach steady state. Steady state growth conditions were maintained for 30 generations. Samples were taken at the Pool (P), Batch (B), Early Steady State (ESS, 48 hours after switching to continuous culture), Mid Steady State (MSS, after 20 generations) and Late Steady State (LSS, after 30 generations) stages then processed for Illumina sequencing of the barcodes to determine the abundance of each strain. Two biological repeats were carried out for each condition. Details of growth medium and continuous culture in chemostats are as previously described [36].

### Genomic DNA extraction, amplification of UPTAG and DOWNTAG barcodes and Illumina sequencing (Bar-Seq)

Genomic DNA was isolated from samples using the Wizard Genomic DNA Purification Kit (Promega) according to the manufacturer’s protocol. UPTAGs and DOWNTAGs were amplified with primers compatible with multiplexed Illumina sequencing. For the UPTAGs the forward primer was 5’AATGATACGGCGACCACCGAGATCTACACTCTTTCCCTACACGCTCTTCCGATCTGATGTCCACGAGGTCTCT and the reverse primer was 5’CAAGCAGAAGACGGCATACGAGATNNNNNNGTGACTGGAGTTCAGACGTGTGCTCTTCCGATCTGTCGACCTGCAGCGTACG. For the DOWNTAGS the forward primer was 5’-AATGATACGGCGACCACCGAGATCTACACTCTTTCCCTACACGACGCTCTTCCGATCTCGAGCTCGAATTCATCGAT and the reverse primer was 5’CAAGCAGAAGACGGCATACGAGATNNNNNNGTGACTGGAGTTCAGACGTGTGCTCTTCCGATCTCGGTGTCGGTCTCGTAG. The NNNNNN represents the 6-mer indexing tag used for multiplexing the different samples. Amplified TAGs were quantified with the KAPA library quantification kit (KAPA Biosysytems) and 10nM of the TAG libraries was used for Illumina sequencing.

### Illumina sequencing data analysis

Sequenced reads were trimmed to contain just the TAG sequence using Trimmomatic [74]. Trimmed reads were mapped to a database of the TAG sequences using Bowtie2 [75]. A TAG was deemed to be identified if the trimmed sequenced read aligned to the full length of that TAG with a maximum of 1 mismatch. Summed counts for each of the two TAGS for a deletion strain were used as input for DESeq [76]. The Log2 fold change was determined between different growth stages and the changes with a p value of < 0.05 and 1.5 fold change were identified.

### Construction of *RUF20* (SUT527) *KanMX-TetO*_*7*_ strain

Two primers, RUF20_P1 and RUF20_P2 (**S18 Table**) were designed to amplify the *KanMX-TetO*_*7*_ cassette from plasmid pCM325 [65]. The resulting *RUF20*-*KanMX-TetO*_*7*_ cassette was transformed into the strain CML476 [65]. The two ends of the cassette were homologous to the start and 500bp upstream of the SUT527 gene to replace 500bp of the SUT527 promoter. The successful transformants were selected on 200μg/ml YPD-G418 plates, confirmed by PCR and the resulting strain was named CML/RUF20/tetO_7._

### Reverse transcription polymerase chain reaction (RT-PCR)

For RT-PCR, mRNA was isolated from 200μg yeast total RNA prepared by the hot phenol method [77] with the SIGMA GenElute mRNA Miniprep kit according to the manufacturers protocol and eluted in 100μl of elution buffer. The polyadenylated mRNA sample was treated with 10 units of RQ1 DNase (Promega) in 1X DNase Buffer (Promega) and 200 units RNasin (Promega) at 30°C for 30 minutes. The reaction was then stopped by adding 2mM of EDTA and incubation at 65°C for 10 minutes. An equal amount of citrate buffered phenol (pH 5.3) was added followed by vortexing for 1 minute and centrifugation at 15600g for 2 minutes. The polyadenylated mRNA was then precipitated from the aqueous phase by adding 0.1 volume of 3M sodium acetate (pH 5.3), 2.5 volumes of 100% ethanol and 10μg of glycogen. The sample was precipitated at -20°C for 30 minutes. The RNA was collected by centrifugation for 5 minutes and washed with 96% ethanol, pelleted again at 15600g and air dried. The pelleted sample was resuspended in 16.25μl of water to be used for the first strand cDNA synthesis using the OneTaq RT-PCR Kit (New England Biolabs). The procedure for cDNA synthesis and PCR amplification was based on the manufacturer’s instructions. Primers for primer walking of the *SEC4* and *TUB2* coding sequence and 3’ UTR are listed in **S18 Table**.

### Quantitative Real Time-PCR (qRT-PCR)

To determine the expression of genes, cells were grown to an OD_600_ of 0.5 prior to RNA extraction using the Qiagen RNeasy Mini Kit. RNA concentrations were determined with a NanoDrop Lite Spectrophotometer. The GoScript Reverse Transcriptase was used for cDNA synthesis with 200ng of RNA. Quantitative RT-PCR was performed on the cDNA using iTaq universal SYBR green Supermix (BioRad) in a CFX Connect Real-time PCR Detection System (BioRad). qPCR cycling conditions were as follows: initial denaturation 95°C for 3 mins; 35 cycles of 95°C for 45 secs, 58°C for 45 secs and 72°C for 3 mins; final extension of 72°C for 5 mins. *ACT1* was used as a reference gene. The Ct values were used to measure the expression of each gene according to the 2^-ΔCt^ method [78]. Sequences for the oligonucleotides used can be found in the **S18 Table**. Using the ΔΔCт method and *ACT1* as a reference gene, the fold change (2^) in expression, relative to the wild-type was calculated. Error bars are calculated using each of the three independent biological samples. P values were calculated using the Welch two sample t-test.

### Cloning of *SEC4* and SUT527 (*RUF20)* genes

The open reading frame plus 500bp upstream and 250bp downstream of *SEC4* (129943–131331), which contains the approximately 1.4kb BamHI/EcoRI fragment that complements *SEC4* function [64] was amplified with Phusion DNA polymerase (New England Biolabs) and primers SEC4F-Bam and SEC4B-Eco (**S18 Table**). The open reading frame plus 500bp upstream and 500bp downstream of SUT527 (13146–14586) was amplified with Phusion DNA polymerase and primers RUF20F-Bam and RUF20B-Xba (**S18 Table**). PCR products were then cloned into pRS413 to produce plasmids pRS413-SEC4 and pRS413-RUF20. The correct *SEC4* and SUT527 (*RUF20)* sequences were confirmed by sequencing.

### Digoxigenin (DIG) and Dinitrophenol (DNP) labelling probes for fluorescent *in situ* hybridization

Plasmids pRS413-SEC4 and pRS413-RUF20 were used as templates for production of transcription templates for *SEC4* and SUT527 (*RUF20*) probes by PCR for digoxigenin or dinitrophenol labelling using primer pairs SEC4T7/SEC4B_prob and RUF20FT7/RUF20B-prob (**S18 Table**). Digoxigenin and dinitrophenol labelled probes were made using 1μg of purified *SEC4* or SUT527 (*RUF20*) PCR template with 1X DIG RNA labelling mix (Roche) or an identical RNA labelling mix containing DNP-11-UTP in place of DIG-11-UTP in a transcription reaction at 37°C for 2 hours using T7 RNA polymerase (Promega) according to the manufacturer’s instructions. Two units of RQ1 DNase (Promega) were then added and the mixture incubated at 37°C for 15 minutes. The sample was then purified using the Qiagen RNA Easy kit following the manufacturer’s instructions. The RNA concentration was measured and 10μg of RNA probe was used for the hybridization step.

### Fluorescence *in situ* hybridization slide preparation

Coverslips No.1 glass 22mm X 22mm (Fisher) were boiled for 30min in 250ml water with 0.1N HCl. Cover slips were then rinsed 10X with deionised water and stored in 70% ethanol. Flamed coverslips were coated with 200μl of 1X poly-L-lysine solution (Sigma) for 2min then excess poly-L-lysine removed and the coverslips air-dried. Coverslips were washed three times with 250μl of water for 10 minutes and air dried. Slides were stored in single wells of a six-well tissue culture dish at room temperature after air drying.

### Cell growth and fixation of yeast

For cell fixation, cells were grown at 30°C in 50ml YPD with our without doxycycline (600μg/mL) to OD_600nm_ = 0.5 and fixed in 4% formaldehyde (Sigma) for 45min at room temperature. Cells were then centrifuged at 2,400g for 5min at 4°C then resuspended in 1ml buffer B (16mM KH_2_PO_4_, 83mM K_2_HPO_4_, 5.4% Sorbitol). Cells were then washed three times with buffer B. Washed cells were resuspended in 1ml freshly-prepared spheroblast buffer (Buffer B with 20mM Vanadyl Ribonucleoside Complex (NEB), 250 units lyticase and 0.002% β-mercaptoethanol) and incubated at 30°C for 15 minutes. Cells were washed twice with 1ml ice cold buffer B and spun at low speed 2000g for 1 minute. Cells were resuspended in 1ml buffer B and 150μl of the cells were placed on coated coverslips and incubated at 4°C for 30 minutes to allow adherence of the cells to the coverslips. Cells were then washed with 5ml ice cold Buffer B and 5ml of 70% ethanol was added, cells were then stored at -20°C.

### Hybridization and detection

The stored coverslips were immersed in 1ml of the hybridization mix (50% formamide, 5X SSC, 1mg/ml yeast tRNA, 100μg/ml heparin, 1X Denhardts, 0.1% Tween 20, 0.1% CHAPS, 5mM EDTA) in a six-well tissue culture dish. The dish was then sealed with parafilm and incubated at 50°C for 1 hour. Next, the hybridization mix was removed and another 2ml of the hybridization mix was added with 10μg probe (either DIG-labelled probe alone for *SEC4* or SUT527/RUF20 for single detection or DIG-labelled probe for *SEC4* and DNP-labelled probe for SUT527/RUF20 for colocalization) then incubated overnight at 50°C. Coverslips were washed with 2ml 0.2X SSC three times. Then 2ml of blocking buffer (1X PBS, 0.1% TritonX-100 and 10% horse serum) was added to the coverslips and incubated at room temperature for 1 hour. For single localization of DIG-labelled probes coverslips were incubated for 2 hrs with HRP conjugated anti-digoxigenin monoclonal antibody (Jackson Immuno Research) diluted to 1:500 with 250μl blocking buffer. Coverslips were then washed three times with 1ml blocking buffer and incubated for 2 hours with Alexa Fluor 488-conjugated anti-HRP antibody (Jackson Immuno Research) diluted 1:100 with 250μl blocking buffer. For combined co-localization detection of DIG- and DNP-labelled probes coverslips were incubated for 2 hrs with goat anti-DIG antibodies (Vector Laboratories) and rabbit anti-DNP (Vector Laboratories) diluted to 1:500 with 250μl blocking buffer. Coverslips were then washed three times with 1ml blocking buffer and incubated for 2 hours with mouse anti-rabbit Alexa Fluor 488 antibody (Jackson Immuno Research) and mouse anti-goat Alexa Fluor 647 antibody (Jackson Immuno Research) each diluted 1:100 with 250μl blocking buffer. Coverslips were then washed three times with 1ml blocking buffer and the coverslips were placed on a slide with a drop of ProLong Gold antifade reagent with DAPI (Molecular Probes by Life Technologies) and allowed to set.

### Fluorescent microscopy and quantification of localization

For single localization slides were visualised with a Nikon Eclipse E600 microscope using a 100x/0.5–1.3 NA differential interference contrast oil Iris Apo objective. The images were captured using a Nikon DS-QilMc camera and NIS-Elements BR 3.2 software. To obtain the quantitative data on RNA localisation in each strain, 100 cells were scored and analyzed for the localization on whether RNA signals were localized to the cell membrane or not. Cells were scored from three technical repeats. For colocalization images were collected on a Zeiss Axioimager.D2 upright microscope using an Olympus UPlanFL 100x/1.30 Oil Ph3 0.17 objective and captured using a Coolsnap HQ2 camera (Photometrics) through Micromanager software v1.4.23. Specific band pass filter sets were used to prevent bleed through from one channel to the next. Images were then processed and analyzed using Image J.

### Fitness growth rate assays of deletion strains (monoculture)

In order to investigate the growth effects of the ncRNA deletions, strains were grown under rich (YPD) and minimal (chemically defined F1 with carbon or nitrogen limitations) media conditions at 30°C. F1 medium was prepared in accordance to Delneri [36]. Carbon and Nitrogen limited F1 media were modified to contain 0.25% glucose (w/v) and 0.46 g/liter (NH_4_)_2_SO_4,_ respectively. Growth measurements at OD_595_ were recorded using a BMG FLUOstar OPTIMA Microplate Reader, as previously described by Naseeb and Delneri [79] for up to 70hr incubation time. Cells were grown at 30°C from an OD_600_ 0.1 and readings taken every 5min. Three technical replicates of three independent biological samples were used for each deletion mutant strain and six technical replicates for the wild type strain. Graphs were produced using the *grofit* package of the *R* program. Area under curve (AUC) measurements for the tA(UGC)O, SUT340, CUT873 and tT(AGU)J deletion mutants were calculated as per Norris *et al* [80], using the grofit::gcFitSpline R package.

### Phenotypic analysis of the haploid deletion collection

To account for plate and batch effects, two biological replicates (MATa and MAT*α*) and four technical replicates of each haploid deletion mutant strain were prepared. Three technical replicates of each plate were performed. Strains were removed from -80°C storage and grown to saturation at 30°C in YPD, in 384 well microtitre plates. Using a Singer Rotor HDA, the 384 well cell cultures were stamped onto YPD plates and incubated at 30°C for 2 days. Plates were then imaged using a Bio-Rad Gel Doc XR system and images processed using SGAtools [81]. The average of the normalized colony size values for replicates of each biological were then combined and used for analysis. We assumed normal distribution on the dataset and used the standard EM algorithm to determine means and standard deviations from the mixture of strains with normal growth and others with reduced fitness using Mixtools [82]. The P values were calculated from the parameters that are closer to the wild-type and fitness differences considered significant with p < 0.05.

### Co-fitness analysis

Sequencing data were normalized and converted to Log2 fold change to allow comparison between the pool and batch stage and between the early and late steady state across different media and temperatures using DESeq2 [83]. To classify the deletion strains based on the impact of growing conditions, we applied generalized linear model with normal approximation and selected those with significant response to our testing variables (P value ≤ 0.05). As a result, fitness profiles were simplified and clustered using the *ad hoc* partitioning around medroid method implemented in the R package cluster [84]. Finally, data analysis was conducted to evaluate enrichment of SUT/CUT using exact binomial test. False discovery rate (FDR) was calculated using R. The biological functions of neighboring genes to ncRNAs in each cluster were identified using GO Term Finder in SGD.

### Sense and antisense ncRNA overexpression plasmid construction

The P_GAL1_ promoter was amplified from pAV1901 [85] using Gal1.for and Gal1.rev primers and cloned into the SalI site in pRS416 [86]. Next the *CYC1* terminator was amplified from p426-GPD [87] using Cyc1.for and Cyc1.rev primers and cloned into the BamHI site creating pRS416Gal1Cyc1. The sense and antisense ncRNA expression plasmids were created using the primer pairs in **S18 Table**. Phusion DNA polymerase (New England Biolabs) was used in all amplifications according to the manufacturers protocol using yeast genomic DNA from BY4742 as template. All sense and antisense ncRNA expression plasmids were cloned into pRS416Gal1Cyc1 via the HindIII restriction site using the Gibson cloning technique [88]. All constructs were verified by sequencing.

### Complementation of ncRNA deletions with ncRNA overexpression plasmids

Each ncRNA overexpression plasmid was transformed into the corresponding heterozygote diploid deletion strain and wild-type BY4743. Cells containing the overexpression plasmids were selected for on SD media lacking uracil (0.67% Bacto yeast nitrogen base without amino acids, 2% glucose, 2% agar, 0.192% Yeast synthetic drop-out medium supplement without uracil). Strains were then sporulated in liquid sporulation medium lacking uracil (1% potassium acetate, 0.005% zinc acetate, 0.002% histidine and 0.003% leucine). Cultures were incubated for 5 days at 25°C followed by three days incubation at 30°C. Tetrad dissection was performed on SD media plates containing 2% Galactose and lacking uracil, using a Singer instruments MSM 400 microdissector. After 4 days incubation at 30°C, tetrad dissection plates were replica plated on to SD media containing 2% Galactose, 300mg/L G418 and lacking uracil. Haploids growing on the final plates were considered to contain the original ncRNA deletion cassette and the ncRNA overexpression plasmid.

DNA was extracted (QIAamp DNA Mini Kit) from these haploids for PCR confirmation. The presence of the ncRNA overexpression plasmid was confirmed using universal pRS416 primers (pRS416 F Primer ‘CATGGAGGGCACAGTTAAGC’ and pRS416 R Primer ‘ACCACATCATCCACGGTTCT’). Deletion of SUT075 was confirmed using a primer specific to the kanamycin cassette (kanC3 ‘CCTCGACATCATCTGCCCAGAT’) and a primer flanking the insertion site (SUT075 confD ‘TGCAGGGAACAGATTTTAGATTT’). PCR reaction mix contained: 0.5μM of each primer, 100ng of DNA template, 12.5μl MyTaq Red Mix (Bioline) and water to 25μl. Cycling conditions: initial denaturation at 95°C for 10min followed by 35 cycles of 95°C for 30sec; 57°C for 30sec; 72°C for 90sec and a final elongation of 72°C for 5min. PCR products were run on a 1.5% agarose gel.

### Co-overexpression of *EMP46* and *GAL2*

*EMP46* and *GAL2* overexpression plasmids were constructed following the same methodology as the ncRNA overexpression above, with a few adjustments. The pBEVY-GA plasmid, containing a bi-directional GAL1/10 promoter, was used [89]. *EMP46* was inserted at the upstream site via the BamHI site and *GAL2* was inserted at the downstream site via the XmaI site. Three plasmids were constructed containing: 1) *GAL2*; 2) *EMP46* or 3) *GAL2* and *EMP46*. These plasmids were transformed separately into BY4743 and selected on SD media lacking uracil (as above). Cultures of these overexpression strains were then serially diluted tenfold and stamped (using the spot assay function of Singer Instrument’s ROTOR) onto SD media containing either 2% Galactose (promoter activate) or 2% Glucose (promoter inactivate). Plates were then imaged using a Bio-Rad Gel Doc XR system.

### SUT075 complementation qRT-PCR

Cultures of BY4743 (+empty pRS416), ΔSUT075 (+empty pRS416) and the ΔSUT075 (+ sense SUT075 recovery plasmid) heterozygote diploid strains were grown to an OD_600_ 0.5 in liquid SD media containing 2% galactose and lacking Uracil. Three biological replicates of each strain were cultured. RNA was extracted and qRT-PCR was performed using the *PRP3* forward and reverse primers (**S18 Table**), following the methods previously described (Quantitative Real Time-PCR).

### Website building

YNCA was developed in RStudio [90], version 1.0.143, with the use of the packages shiny [91] and rmarkdown [92]. Local server hosting relies on the open source version of Shiny-server. The underlying server-side data processing is written in R [93], version 3.4.0. Lists and positions of chromosomal features in *S*. *cerevisiae* are taken from the Saccharomyces Genome Database (www.yeastgenome.org). The type of features included are: known opening reading frames, tRNA genes, snoRNA genes, centromeric and telomeric regions, autonomous replicating sequences (ARS), long terminal repeats (LTR), pseudogenes, LTR retrotransposons and transposon internal genes.

## Supporting information

S1 Table Deleted ncRNAs. List of deleted ncRNAs, their genomic coordinates, distances to closest protein start codons, deletion cassette primers, assigned Tag numbers and barcode Tag sequences, deletion confirmation primer sequences and confirmation PCR product sizes.
(XLSX)

S2 Table Deletion collections. Table contains four sheets: first sheet contains the list of the heterozygous diploid collection with identified essential ncRNAs;the second sheet contains the list of the haploid *a* mating type collection;the third sheet contains the list of the haploid α mating type collection; the fourth sheet contains the list of the homozygous diploid collection.
(XLSX)

S3 Table Pool vs Batch and ESS vs LSS comparisons. Log2 Fold changes and P values for Figs 1 and 3.
(XLSX)

S4 Table Carbon-limited 30°C data. Carbon-limited 30°C data with Pool vs Batch plots and ESS vs LSS plots.
(XLSX)

S5 Table Carbon-limited 36°C data. Carbon-limited 36°C data with Pool vs Batch plots and ESS vs LSS plots.
(XLSX)

S6 Table Carbon-limited LiCl 30°C data. Carbon-limited 30°C LiCl data with Pool vs Batch plots and ESS vs LSS plots.
(XLSX)

S7 Table Nitrogen-limited 30°C data. Nitrogen-limited 30°C data with Pool vs Batch plots and ESS vs LSS plots.
(XLSX)

S8 Table Nitrogen-limited 36°C data. Nitrogen-limited 36°C data with Pool vs Batch plots and ESS vs LSS plots.
(XLSX)

S9 Table Nitrogen-limited LiCl 30°C data. Nitrogen-limited 30°C data with LiCl Pool vs Batch plots and ESS vs LSS plots.
(XLSX)

S10 Table Heterozygote collection–pool vs batch Top 50 and Bottom 50 most haplo-proficient or haplo-insufficient. (PDF)

S11 Table Heterozygote collection–ESS vs LSS Top 50 and Bottom 50 most haplo-proficient or haplo-insufficient. (PDF)

S12 Table Summary of heatmap data for Fig 4. (XLSX)

S13 Table Heterozygote collection—genes listed according to co-fitness groupings. (PDF)

S14 Table Haploid collection fitness data. (XLSX)

S15 Table Haploid collection–Top 50 and Bottom 50 most haplo-proficient or haplo-insufficient. (PDF)

S16 Table Comparison of tRNA deletions in this study with tRNA deletions in Bloom-Ackermann et al (2014). (PDF)

S17 Table Strains. (PDF)

S18 Table Primers. (PDF)

S1 Fig Fitness profiles of the tR(CCU)J, CUT248 and SUT233/CUT707 heterozygous ncRNA deletion mutants in rich and minimal media conditions at 30°C. ncRNA deletion strains were tested individually for their fitness in YPD (A), nitrogen-limited (B) and carbon-limited (C) chemically defined F1 media at 30°C. Growth curves shown are expressed as the mean growth from three replicates of three independent biological strains for each deletion and from six replicate cultures for the wild type (BY4743) strain. Limitations in nitrogen or carbon sources are indicated as Nlim (B) and Clim (C), respectively. Error bars are present in all points and are indicated as the standard deviations from the replicates. All strains included in the growth assays are represented by different colors as described in panel D.
(TIF)

S2 Fig Overexpression of the CUT248 RNA sequence from a plasmid in a wild-type haploid strain BY4741 results in a slow growth phenotype. The RNA sequence for CUT248 was cloned into a yeast expression vector under control of the yeast *GAL1* promoter. The vector was transformed into the wild-type BY4741 haploid strain and six independent single colonies (1–6) were spotted by serial dilution on SD-Ura plates with either glucose or galactose containing plates. Plates were incubated at 30°C for 48hrs. The expression vector alone (P), the vector expressing the gene *WWM1* known to cause lethality when overexpressed (N) and the BY4741 strain alone were also spotted by serial dilution on the same plates.
(TIF)

S3 Fig Monoculture validation of selected haplo-proficient and haplo-insufficient phenotypes. Monoculture validation, in a microplate reader, of four strains identified as being haplo-insufficient or haplo-proficient during competition experiments in continuous culture. Box plots are constructed using the area under curve (AUC) as parameter. (A) SUT340 and tA(UGC)O heterozygote diploid deletion strains grown in C-limited F1 media at 36°C. (B) CUT873 and tT(AGU)J heterozygote diploid deletion strains grown in C-limited F1 media at 30°C.
(TIF)

S4 Fig Upset plots to visualize common haplo-insufficient (A) and haplo-proficient (B) fitness profiles between different conditions in Batch to Pool experiments. Horizontal bars for each condition shows the total number of strains with significant fitness differences at Log2 fold change greater than 1.50 and p-value less than 0.05. Connected black circles indicate common profiles across different conditions with vertical bars showing the number of intersections.
(TIF)

S5 Fig Upset plots to visualize common haplo-insufficient (A) and haplo-proficient (B) fitness profiles between different conditions in LSS to ESS experiments. Horizontal bars for each condition shows the total number of strains with significant fitness differences at Log2 fold change greater than 1.50 and p-value less than 0.05. Connected black circles indicate common profiles across different conditions with vertical bars showing the number of intersections.
(TIF)

S6 Fig Selected data from co-fitness analysis. Fitness profiles of selected ncRNA deletion strains (A) SUT643; (B) SUT471; (C) SUT509. Heights represent Log2 fold change between batch and pool or late and early steady state across the eight growth conditions (B_P:comparison between batch and pool; L_E: comparison between late and early steady state; Clim: carbon-limited medium; Nlim: nitrogen-limited medium). Colours represent direction of fitness changes. Haplo-insufficiency is shown in blue, and haplo-proficiency is shown in bright red.
(TIF)

S7 Fig Biological replicates of tetrad dissections for essential ncRNAs showing lethality. Two additional biological replicates of the diploid knockout strains found to be essential with the first replicate were sporulated and tetrads dissected to determine essentiality. All replicates displayed a pattern (2 viable, 2 lethal) consistent with all the ncRNA deletions being essential.
(TIF)

S8 Fig Shorter deletions of SUT527 that do not overlap with *SEC4* are not essential. (A) The different lengths of SUT527/*RUF20* deletions are represented as blue arrows containing the number of nucleotides deleted. Arrows with a blue gradient indicate that the deletion has disrupted the 3’ UTR of *SEC4*. (B) Only two viable spores grew after diploid sporulation and dissection for the 342nt deletion of SUT527/*RUF20*, indicating that this region of SUT527/*RUF20* is still essential (left panel). Four viable spores grew after diploid sporulation and tetrad dissection for the 242nt deletion of SUT527/RUF20, indicating that it is not essential (right panel).
(TIF)

S9 Fig Double stranded RNA is formed in the region of *SEC4*/SUT527 when RNAi is introduced into yeast cells. Screen shot from genome browser for visualization of processed small RNA-seq data for Genome-wide mapping of dsRNA from Wery et al. 2016, Molecular Cell 61:3790–392 (http://vm-gb.curie.fr/mw2). Red lines define the limits of the *SEC4* transcript. Black arrows point to the region of overlap between *SEC4* 3’ UTR and SUT527. dsRNA (red peaks) is detected upon RNAi reconstitution.
(TIF)

S10 Fig No change in SUT690 expression in the SUT259/691 deletion strain. Real time PCR results to measure expression levels of SUT690 in the ΔSUT259/691 heterozygote diploid deletion strain. The relative expression of SUT690 in the wild-type background is represented by a shaded grey bar and as a shaded black bar in ΔSUT259/691 strain background. Using the ΔΔCт method and *ACT1* as a reference gene, the fold change (2^) in expression, relative to the wild-type was calculated. Error bars are calculated using each of the three independent biological samples. P values calculated using the Welch two sample t-test. There is no significant difference (*p* = 0.72) in SUT690 expression between the wild type and ΔSUT259/691 strains.
(TIF)
