# Supplementary material for: Large-scale profiling of noncoding RNA function in yeast
Source: PLoS Genet. 2018 Mar 12;14(3):e1007253. doi: 10.1371/journal.pgen.1007253 (PMC5864082; doi:10.1371/journal.pgen.1007253)
Supplement: S10 Table — (PDF) [file pgen.1007253.s010.pdf]

S10 TABLE

HETEROZYGOTE COLLECTION - Genes listed in order of most haploproficient or haploinsufficient from top left down each column to bottom right.

C-limited 30°C Pool vs Batch top 50 haploproficient

SNR39B	SNR190	SUT367	SUT571
SNR70	CUT007	tG(GCC)G1	tL(UAA)B2
tD(GUC)I2	SNR37	SNR87	tR(UCU)M1
tE(CUC)D	SUT346	SUT142	tR(UCU)J1
SNR86	tN(GUU)L	tL(UAA)D	SNR32
tQ(UUG)D3	SNR85	tH(GUG)G2	tL(CAA)M
SUT014	tE(UUC)K	tG(GCC)D2	tE(UUC)M
SNR56	tQ(UUG)E2	CUT213	SUT193
SUT042	tN(GUU)F	SNR46	tL(CAA)G2 SUP54
SNR65	tQ(UUG)C	tE(UUC)G2	tE(UUC)K SOE1
SUT055	SNR80	tG(CCC)D SUF3	tI(AAU)N2
tQ(CUG)M CDC65	SUT085	tQ(UUG)E1	
tC(GCA)G	SNR63	SUT518	

C-limited 36°C Pool vs Batch top 50 haploproficient

CUT060	tD(GUC)I2	tC(GCA)G	tL(UAA)D
SUT010	SNR190	tR(UCU)M1	tN(GUU)L
CUT248	tE(CUC)D	tQ(CUG)M CDC65	tL(CAA)G2 SUP54
SNR39B	SUT346	tD(GUC)I1	SNR80
SUT042	SUT014	SUT518	SUT571
CUT007	tQ(UUG)E2	SUT367	tI(AAU)P1
SNR70	SNR65	SNR51/70/41	CUT213
SNR86	tQ(UUG)D3	SUT193	SUT289/717
SNR56	SUT055	SNR32	tH(GUG)H
tX(XXX)D	SUT085	SNR87	tL(CAA)D
SUT087	SNR63	SUT142	SUT477
SUT019	tE(UUC)I	tL(CAA)N	
SNR85	SNR40	tE(UUC)K	

C-limited 30°C 100mM LiCl Pool vs Batch top 50 haploproficient

tT(AGU)J	tQ(CUG)M CDC65	SNR51/70/41	tE(UUC)E1
SUT348	SUT193	tC(GCA)G	tV(UAC)D
tG(GCC)G2	SNR86	tE(UUC)K	tQ(UUG)C
tP(UGG)A TRN1	SNR65	tN(GUU)F	tR(UCU)M1
CUT873	SNR56	tP(UGG)O1	tH(GUG)G2
tY(GUA)J2 SUP4	SNR70	tP(UGG)N1	tT(CGU)K TRT2
tG(GCC)P1	tE(CUC)D	SUT414	SUT142
tD(GUC)I2	SNR87	SUT346	tD(GUC)J2
CUT060	tW(CCA)J	SUT469	SNR62
SNR37	SUT042	SNR39B	tL(CAA)G1
tV(AAC)G3	SUT085	tG(UCC)O SUF1	tS(GCU)F
tN(GUU)O2	SUT055	SNR80	
CUT213	SUT087	SNR190	

N-limited 30°C Pool vs Batch top 50 haploproficient

CUT248	SNR37	CUT060	tE(UUC)K
SNR41	tE(CUC)D	SNR65	tC(GCA)G
SUT010	SNR86	tPUGG)F SUF9	SNR63
SUT019	SUT014	SNR190	CUT213
tA(UGC)A	SNR56	tT(UGU)G1	SUT367
SUT001	SNR70	SUT098	SNR32
SUT087	tQ(UUG)D3	SUT346	SNR128
SNR35	tQ(UUG)E2	SUT085	SUT193
SUT170	SUT643	tQ(CUG)M CDC65	tS(GCU)F
SNR78	CUT007	tL(GAG)G	SNR81
SNR51/70/41	SUT042	SUT340	SNR46
SNR39B	SNR85	SNR40	
SUT089	tD(GUC)I2	SUT055	

N-limited 36°C Pool vs Batch top 50 haploproficient

CUT060	SNR85	SNR63	SNR62
SUT087	tN(GUU)O2	tQ(UUG)E2	tE(UUC)K
SUT010	SNR39B	tP(UGG)O1	SUT289/717
SUT042	SNR87	SUT367	SUT142
CUT248	SUT019	tG(GCC)P1	tR(UCU)M1
tD(GUC)I2	SUT346	tD(GUC)I1	SUT518
SNR65	SUT085	SNR45	CUT213
SNR70	SNR190	CUT873	SNR40
SNR86	tQ(CUG)M CDC65	SUT441	SNR46
tD(GUC)K	SNR51/70/41	tC(GCA)G	tE(UUC)I
tE(CUC)D	SUT348	SNR32	SUT285
SNR56	tQ(UUG)D3	tL(UAA)D	
tX(XXX)D	SUT055	SUT354	

N-limited 100mM LiCl 30°C Pool vs Batch top 50 haploproficient

CUT248	SNR86	CUT060	tQ(CUG)M CDC65
SNR41	SNR190	SUT042	SNR63
SUT010	SNR85	SNR51/70/41	tL(GAG)G
SUT001	SUT170	SUT340	tE(UUC)K
SUT019	SNR70	SNR65	CUT007
tA(UGC)A	tD(GUC)I2	SUT367	tC(GCA)G
SNR43	tQ(UUG)D3	SUT055	tH(GUG)G2
SUT087	SNR56	CUT816	SNR81
SNR35	SUT014	SUT085	tD(GUC)I1
SNR39B	SUT643	SUT346	SNR46
SNR78	tQ(UUG)E2	SNR32	SNR128
SNR37	tP(UGG)F SUF9	SUT098	
tE(CUC)D	tT(UGU)G1	SNR40	

C-limited 30°C Pool vs Batch top 50 haploinsufficient

tR(CCU)J HSX1	tT(AGU)D	SUT285	tH(GUG)E2
tF(GAA)B	CUT356	tK(CUU)G1	SNR83
SUT167	SUT125	SUT617	tE(CUC)I
tK(CUU)G3	tV(AAC)O	tS(AGA)J	tD(GUC)N
SUT211	U4 snRNA SNR14	CUT572	CUT005
tD(GUC)J1	tR(CCG)L TRR4	tP(UGG)A TRN1	SUT463
tV(CAC)D	SUT543/115	SNR64	SNR53
tV(UAC)D	tA(UGC)O	tR(UCU)B	SUT591
SNR48	U2 snRNA snR20 LSR1	CUT378	tL(CAA)L
CUT547	SUT004	tG(GCC)D1	CUT150
CUT827	CUT523	tG(GCC)C SUF16	SNR45
tR(UCU)E	SUT480	SUT056	CUT442
SUT509		tD(GUC)M	

C-limited 36°C Pool vs Batch top 50 haploinsufficient

tR(CCU)J HSX1	tA(AGC)D	tV(AAC)G1	U2 snRNA snR20 LSR1
tA(UGC)O	tA(UGC)L	CUT374	tR(UCU)G1
CUT547	tS(AGA)M	CUT005	tT(AGU)H
SUT167	SUT617	CUT572	tD(GUC)M
tV(CAC)D	tE(UUC)P	tS(AGA)J	SUT004
SUT233/CUT707	tT(AGU)D	SUT218/655	tR(UCU)B
tF(GAA)B	tR(UCU)E	tV(AAC)O	CUT634
tK(CUU)G3	SUT340	tD(GUC)N	tQ(UUG)D1
SUT456/029	SUT509	SNR61	tG(GCC)J1
tV(AAC)K1	CUT356	CUT827	SNR64
SUT211	SUT119	U4 snRNA SNR14	tT(AGU)O2
tD(GUC)J1	tW(CCA)P	SUT125	
tV(UAC)D	SNR48	SUT480	

C-limited 30°C 100mM LiCl Pool vs Batch top 50 haploinsufficient

tR(CCU)J HSX1	tD(GUC)M	CUT356	tR(UCU)B
tK(CUU)G3	tR(UCU)E	tK(CUU)G1	CUT277
SNR13	tH(GUG)E2	tN(GUU)K	SUT004
tQ(UUG)L	SNR64	tT(AGU)D	SUT084/SUT499
U2 snRNA snR20 LSR1	SNR83	SUT824	CUT461
tV(CAC)D	SUT463	U4 snRNA SNR14	tD(GUC)J1
CUT150	tG(GCC)C SUF16	SUT471	SNR47
tA(AGC)P	tL(CAA)L	SUT167	tW(CCA)M
tG(GCC)D1	SUT457	U1 snRNA SNR19	tQ(UUG)D2
tR(CCG)L TRR4	tK(UUU)O	tF(GAA)B	SUT083
SNR48	CUT523	tV(AAC)O	SUT770
tE(CUC)I	CUT005	SUT509	SUT543/115
	SUT480	SUT617	

N-limited 30°C Pool vs Batch top 50 haploinsufficient

- tR(CCU)J HSX1
- SUT233/CUT707
- tV(CAC)D
- tW(CCA)P
- SUT004
- SUT167
- tI(AAU)E2
- tR(UCU)E
- tV(AAC)G1
- tK(CUU)G3
- tV(AAC)O
- SUT211
- SNR48
- tG(GCC)F2
- tA(UGC)L
- tT(AGU)D
- CUT356
- SUT617
- tV(UAC)D
- tF(GAA)B
- U2 snRNA snR20 LSR1
- tD(GUC)J1
- SNR64
- tR(CCG)L TRR4
- CUT547
- tG(GCC)C SUF16
- SUT218/655
- SUT125
- tA(AGC)D
- CUT005
- tD(GUC)M
- SUT480
- tH(GUG)E2
- tT(AGU)H
- tA(UGC)O
- tK(CUU)C
- SUT737/313
- tG(GCC)D1
- SUT456/029
- tA(AGC)M2
- U4 snRNA SNR14
- tS(AGA)J
- tT(AGU)O2
- tT(CGU)K TRT2
- SUT119
- SNR83
- tR(ACG)L
- tL(CAA)L
- tK(CUU)G1
- tK(UUU)O

N-limited 36°C Pool vs Batch top 50 haploinsufficient

- tR(CCU)J HSX1
- tA(UGC)O
- SUT233/CUT707
- SUT456/029
- CUT005
- tR(UCU)E
- tS(AGA)M
- tA(AGC)D
- tA(UGC)L
- tV(CAC)D
- CUT356
- tV(AAC)K1
- tQ(UUG)L
- SUT340
- tA(AGC)P
- tD(GUC)M
- SUT617
- SUT119
- tW(CCA)P
- tH(GUG)E2
- tG(GCC)D1
- tE(UUC)P
- tD(GUC)N
- tV(AAC)G1
- tG(GCC)C SUF16
- SUT125
- SNR83
- SNR48
- SUT480
- SNR64
- tK(CUU)G3
- tV(AAC)M1
- CUT374
- tR(UCU)G1
- U2 snRNA snR20 LSR1
- SNR61
- tT(AGU)D
- SUT463
- tL(CAA)L
- tT(AGU)H
- tT(AGU)O2
- tR(UCU)B
- tE(CUC)I
- SUT167
- SUT218/655
- tV(AAC)O
- CUT523
- tG(GCC)J1
- U4 snRNA SNR14
- tK(CUU)G1

N-limited 30°C 100mM LiCl Pool vs Batch top 50 haploinsufficient

- tR(CCU)J HSX1
- tV(CAC)D
- tI(AAU)E2
- SUT233/CUT707
- tR(UCU)E
- tK(CUU)G3
- SUT004
- SUT167
- tW(CCA)P
- CUT356
- tG(GCC)F2
- SUT617
- SUT211
- tA(UGC)L
- tV(AAC)O
- tV(AAC)G1
- U2 snRNA snR20 LSR1
- SUT218/655
- tF(GAA)B
- SNR48
- tV(UAC)D
- tT(AGU)D
- tA(AGC)D
- tT(AGU)H
- tD(GUC)J1
- tG(GCC)C SUF16
- SNR64
- tK(CUU)C
- SUT456/029
- tR(CCG)L TRR4
- SUT125
- CUT005
- tH(GUG)E2
- SUT480
- tA(AGC)M2
- U4 snRNA SNR14
- SUT737/313
- tD(GUC)M
- tT(AGU)O2
- SNR53
- tG(GCC)D1
- SNR83
- CUT547
- CUT523
- tS(AGA)A
- tE(UUC)P
- tK(CUU)G1
- tS(AGA)J
- SUT509
- tL(CAA)L
