# Supplementary material for: Large-scale profiling of noncoding RNA function in yeast
Source: PLoS Genet. 2018 Mar 12;14(3):e1007253. doi: 10.1371/journal.pgen.1007253 (PMC5864082; doi:10.1371/journal.pgen.1007253)
Supplement: S11 Table — (PDF) [file pgen.1007253.s011.pdf]

S11 TABLE

HETEROZYGOTE COLLECTION - Genes listed in order of most haploproficient or haploinsufficient from top left down each column to bottom right.

C-limited 30°C ESS vs LSS top 50 haploproficient

CUT332	SNR53	SNR58	tE(UUC)E3
SUT471	tR(UCU)D	tS(AGA)D3	CUT296
SUT089	SUT643	SUT229/661	SUT170
CUT827	tQ(UUG)E2	tX(XXX)D	CUT645
tA(AGC)F	tV(AAC)O	tD(GUC)N	SNR62
CUT002	CUT356	tG(GCC)J2	ncRNA NME1
tN(GUU)C	CUT248	tD(GUC)M	SUT347
tL(UAA)L	SUT285	U4 snRNA SNR14	tY(GUA)J2 SUP4
SNR60	SUT824	tW(CCA)M	tP(UGG)H SUF8
CUT170	SUT602/176	SUT437	U6 snRNA SNR6
SNR77	CUT008	tI(AAU)I2	SUT496
tK(UUU)K	tT(AGU)J	SNR50	
tV(AAC)G2	tN(GUU)N1	SUT274/698	

C-limited 36°C ESS vs LSS top 50 haploproficient

SUT471	tK(UUU)G2	SNR17A	SNR43
tN(GUU)G	tL(UAA)J SUP51	U6 snRNA SNR6	CUT572
tA(AGC)G	SUT019	tC(GCA)P1	tH(GUG)G2
SUT354	SUT167	SNR45	CUT356
SNR53	tV(AAC)M3	tX(XXX)D	SNR52
tW(CCA)J	SUT173	SNR57	tC(GCA)G
SUT170	tQ(UUG)D2	SNR66	tV(UAC)B
tV(CAC)H	tV(UAC)D	SNR48	tN(GUU)P
SUT456/029	tT(CGU)K TRT2	SUT737/313	tA(AGC)M2
SUT056	SUT089	tS(AGA)D3	tE(UUC)K
tV(AAC)O	tP(UGG)A TRN1	SUT086/CUT102	tI(UAU)L
SUT035	SUT492	CUT213	
SUT518	tL(UAA)L	SUT284/712	

C-limited 30°C 100mM LiCl ESS vs LSS top 50 haploproficient

tG(GCC)B	tK(CUU)E2	tQ(UUG)B	SNR51/70/41
tH(GUG)E2	SNR72	tT(AGU)I1	SNR48
tK(UUU)P	SUT119	tA(AGC)K2	tT(UGU)G1
SUT493	SUT617	tN(GUU)F	SNR13
CUT356	SNR80	tA(UGC)E	CUT084/SUT068
tR(ACG)D	SUT355	tA(UGC)G	tR(CCU)J HSX1
tG(GCC)M	SUT123	tG(GCC)F2	CUT103
tG(GCC)J1	tC(GCA)B	tK(CUU)I	SNR53
SUT218/655	tI(AAU)L2	SUT142	SNR8
SNR3	CUT374	tS(AGA)B	SNR47
CUT008	tV(CAC)D	tT(AGU)N1	tI(AAU)E1
tL(UAA)L	tV(AAC)J	tT(UGU)H	
tE(UUC)E3	tI(AAU)N1	SNR50	

N-limited 30°C ESS vs LSS top 50 haploproficient

CUT083	tD(GUC)B	tR(UCU)G1	SNR48
ty(GUA)M1 SUP5	SUT014	SUT518	SUT119
SUT056	tV(AAC)K1	SNR61	tS(AGA)B
SUT834	SNR32	SNR64	tS(AGA)M
SUT089	tP(UGG)M SUF7	SUT285	SUT616
tI(AAU)P2	tV(CAC)D	SNR30	tR(CCU)J HSX1
tP(UGG)L	tT(AGU)I1	SUT339	tT(AGU)D
CUT474	CUT103	tT(AGU)H	tV(AAC)H
tK(CUU)F	SNR67	SUT480	SNR45
tP(UGG)O3	CUT001/437	SUT004	tG(GCC)O1
SUT311	SNR128	SUT170	tW(CCA)G1
tT(UGU)G1	SUT347	SUT433	
SUT042	SUT643	SNR83	

N-limited 36°C ESS vs LSS top 50 haploproficient

tG(GCC)B	tY(GUA)J2 SUP4	SNR84	tK(UUU)K
SUT467	CUT298/771	tS(GCU)F	SNR53
tA(UGC)E	tI(AAU)L1	tD(GUC)N	SUT493
tG(GCC)M	tT(CGU)K TRT2	tE(UUC)G3	tG(GCC)F2
tH(GUG)E2	CUT572	tD(GUC)M	SUT218/655
tG(GCC)J1	tT(AGU)O2	SNR76	SUT170
SNR46	tS(AGA)A	tT(AGU)H	SUT056
tQ(UUG)D2	tS(AGA)G	CUT441	SUT501
tS(AGA)D1	tR(ACG)D	tV(UAC)B	U6 snRNA SNR6
tT(AGU)J	tK(UUU)P	SUT471	SNR78
tW(CCA)J	SUT346	SUT125	SNR72
tR(UCU)E	tE(UUC)E3	SNR128	
tW(CCA)G2	CUT356	SUT089	

N-limited 30°C 100mM LiCl ESS vs LSS top 50 haploproficient

SUT471	tR(UCU)D	tP(UGG)N1	SUT239/679
SUT098	SUT267/697	SUT125	tG(GCC)O1
tT(AGU)J	SUT842/425	tT(CGU)K TRT2	tE(UUC)K SOE1
tE(UUC)G3	tI(AAU)L1	SUT056	U6 snRNA SNR6
SNR128	tD(GUC)D	CUT873	tA(AGC)K2
SNR61	SNR41	SUT271	SNR57
CUT458	tW(CCA)G1	SUT493	SUT456/029
SNR48	SUT107	tW(CCA)J	tT(UGU)G1
tP(UGG)A TRN1	SUT123	CUT213	tI(UAU)D
SNR53	SUT019	tN(GUU)F	SUT014
tV(UAC)D	tY(GUA)J2 SUP4	tF(GAA)B	tK(UUU)G2
SUT170	SNR51/70/41	SNR39B	
SUT087	SNR58	SUT171	

C-limited 30°C ESS vs LSS top 50 haploinsufficient

SUT107	tP(UGG)A TRN1	SNR39B	tV(AAC)M2
tQ(UUG)L	SNR85	SUT414	SNR9
SNR34	tE(UUC)J	tG(GCC)O1	SUT571
tA(AGC)P	SUT085	SUT067	tR(CCG)L TRR4
SUT591	SUT500	CUT873	tI(AAU)P2
tK(CUU)G1	SUT593	tL(UAG)L1	tG(GCC)G2
tD(GUC)K	SUT348	SUT014	SUT441
SNR13	SNR190	SUT267/697	tL(CAA)N
SUT361	tD(GUC)J2	SUT019	SNR70
SUT083	tL(UAA)K	tY(GUA)F2 SUP6	tH(GUG)K
tK(CUU)K	CUT123	tL(CAA)G2 SUP54	tV(AAC)J
SUT098	SUT142	tI(AAU)G	
SUT157/579	tE(UUC)E1	SUT042	

C-limited 36°C ESS vs LSS top 50 haploinsufficient

SUT107	tN(GUU)C	CUT436	SNR85
SUT496	CUT007	tA(AGC)L	U1 snRNA SNR19
SUT457	SUT348	SUT539	tG(UCC)G SUF4
CUT150	tL(UAA)K	SUT071/483	SUT085
SNR13	tG(GCC)D1	tR(CCG)L TRR4	tE(UUC)E1
SNR190	SUT500	SUT463	tV(AAC)G2
tV(AAC)M2	CUT523	CUT008	tD(GUC)I1
tQ(UUG)L	tA(AGC)P	tA(AGC)F	tH(GUG)K
tD(GUC)M	tW(CCA)M	SUT264	tL(UAG)L2
tK(CUU)K	SNR72	tY(GUA)J1 SUP7	CUT123
tK(UUU)O	tH(GUG)E2	tV(AAC)J	SNR41
SUT602/176	SUT067	tD(GUC)J1	
tG(GCC)G2	tL(UAG)L1	tI(AAU)I1	

C-limited 30°C 100mM LiCl ESS vs LSS top 50 haploinsufficient

tV(AAC)G3	CUT873	tA(AGC)G	SNR45
tV(UAC)D	SUT285	tS(AGA)E	tE(UUC)I
SNR76	tR(UCU)M1	SNR78	tQ(CUG)M CDC65
SUT581	tQ(UUG)L	SUT414	tS(AGA)D1
tD(GUC)K	tG(GCC)C SUF16	SUT469	tD(GUC)O
SUT193	SNR17A	SUT830	CUT248
tQ(UUG)D2	SNR32	tF(GAA)B	CUT217
SNR87	tN(GUU)O2	tG(CCC)D SUF3	tE(UUC)E1
tP(UGG)A TRN1	SNR42	tQ(UUG)E2	SNR73
SUT211	tE(UUC)K	SNR9	SNR161
tE(UUC)C	tE(CUC)D	SUT010	SUT289/717
tG(GCC)P1	SUT085	CUT244	
SUT348	tG(GCC)G2	SNR64	

N-limited 30°C ESS vs LSS top 50 haploinsufficient

tG(GCC)D2	tG(GCC)G1	SUT099	SUT725
tE(UUC)M	tS(AGA)E	tK(CUU)P	tT(AGU)B
tW(CCA)G2	CUT465	CUT315	SUT501
tL(UAA)D	SUT451	tH(GUG)G2	tI(AAU)P1
SUT157/579	ncRNA NME1	tE(UUC)J	SNR9
tI(AAU)N1	tV(AAC)K2	SNR17B	SUT129
SNR34	tD(GUC)I2	tL(UAA)N	tK(UUU)D
tE(CUC)D	SUT058	CUT468	tL(UAA)K
SUT289/717	SUT827	tF(GAA)H1	CUT775/SUT307
SUT532	tE(UUC)B	tL(CAA)K	tK(CUU)G1
tS(AGA)H	tH(GUG)M	SUT571	tR(ACG)E
SNR69	tG(GCC)P1	tY(GUA)O SUP3	
tV(AAC)E1	SUT430	ncRNA TLC1	

N-limited 36°C ESS vs LSS top 50 haploinsufficient

CUT827	CUT666	tL(CAA)K	SNR9
SNR45	SUT532	SNR56	SUT477
SUT414	tV(UAC)D	SUT067	tN(GUU)O1
SNR17A	tN(GUU)C	tI(AAU)P1	SUT413
U1 snRNA SNR19	SUT058	CUT379	tK(UUU)O
SUT420/CUT425	SNR4	tI(AAU)B	CUT775/SUT307
tA(AGC)L	tL(UAG)L1	SUT722	SNR55
SUT764	SUT107	tL(UAA)D	tY(GUA)M1 SUP5
SNR30	tY(GUA)D SUP2	tR(ACG)K	tP(UGG)A TRN1
tD(GUC)J1	SUT431	tK(UUU)D	SUT725
SUT007	SUT285	SNR190	SNR17B
SUT167	CUT764	tF(GAA)N	
tK(CUU)D1	SUT398	SNR36	

N-limited 30°C 100mM LiCl ESS vs LSS top 50 haploinsufficient

SUT602/176	SNR69	tK(UUU)L	tK(CUU)D2
tD(GUC)K	tF(GAA)H2	CUT244	SNR72/73/74/75/76/77/78
tI(AAU)N1	SUT593	SUT540	CUT468
tH(GUG)G1	SNR9	tL(UAA)D	tE(UUC)J
SUT289/717	ncRNA TLC1	SUT558	SUT532
tS(AGA)D3	ncRNA RPR1	tK(CUU)D1	SNR63
SUT764	SUT441	tT(UGU)H	tG(GCC)F1 SUF20
SNR85	SNR4	tD(GUC)M	tY(GUA)J1 SUP7
SUT157/579	tQ(UUG)C	SUT385	SUT099
tH(GUG)H	tD(GUC)G2	SNR71	SNR31
SUT827	tR(ACG)E	CUT084/SUT068	tF(GAA)N
SUT355	tH(GUG)M	tC(GCA)B	
SUT543/115	tR(ACG)J	CUT001/437	
