# Supplementary material for: Large-scale profiling of noncoding RNA function in yeast
Source: PLoS Genet. 2018 Mar 12;14(3):e1007253. doi: 10.1371/journal.pgen.1007253 (PMC5864082; doi:10.1371/journal.pgen.1007253)
Supplement: S13 Table — (PDF) [file pgen.1007253.s013.pdf]

S13 TABLE

HETEROZYGOTE COLLECTION - Genes listed according to co-fitness groupings

Group 1 (149)

CUT005
CUT008
CUT103
CUT123
CUT296/SUT301
CUT320
CUT374
CUT436
CUT441
CUT523
CUT672
CUT775/SUT307
ncRNA RUF21
ncRNA TLC1
SNR17A
SNR17B
SNR31
SNR34
SNR35
SNR42
SNR43
SNR48
SNR5
SNR53
SNR53/67
SNR55
SNR57
SNR61
SNR69
SNR70
SNR72/73/74/75/76/77/78
SNR79
SNR8
SNR81
SNR83
SNR85
SNR9
SUT001
SUT010
SUT055
SUT087
SUT089
SUT123
SUT125
SUT126
SUT157/579
SUT170
SUT171
SUT211
SUT218/655
SUT233/CUT707
SUT259/691
SUT289/717
SUT305
SUT339
SUT346
SUT347
SUT375
SUT385
SUT418
SUT457
SUT463
SUT465
SUT469
SUT480
SUT493
SUT515
SUT518
SUT532
SUT553
SUT570
SUT643
SUT729/CUT773
SUT737/313
SUT798/387
SUT827
SUT830
tA(AGC)G, tA(AGC)J
tA(AGC)K1
tA(AGC)K2
tA(AGC)M1
tA(AGC)M2
tA(UGC)G
tA(UGC)L
tA(UGC)O
tD(GUC)D
tD(GUC)G2
tD(GUC)J4
tD(GUC)N
tE(UUC)G1 (SOE1)
tE(UUC)J
tE(UUC)M
tF(GAA)P2
tG(GCC)B
tG(GCC)E
tG(GCC)F1 SUF20
tG(GCC)F2
tG(GCC)G1
tG(GCC)J1
tG(GCC)O2 (SUF17)
tI(AAU)B
tI(AAU)I1
tI(AAU)I2
tI(UAU)D
tK(CUU)C
tK(CUU)D1
tK(CUU)G1
tK(CUU)I
tK(CUU)M
tK(CUU)P
tK(UUU)K
tL(CAA)C SUP53
tL(GAG)G
tL(UAG)L1
tM(CAU)C
tN(GUU)C
tN(GUU)P
tP(UGG)M SUF7
tP(UGG)O3
tQ(UUG)D2
tR(ACG)K
tR(ACG)L
tR(CCU)J (HSX1)
tR(UCU)B
tR(UCU)D
tS(AGA)D1
tS(AGA)D3
tS(AGA)G
tS(AGA)J
tS(GCU)F
tT(AGU)B
tT(AGU)H
tT(AGU)I2
tT(AGU)N1
tT(AGU)N2
tV(AAC)G1
tV(AAC)G3
tV(AAC)K1
tV(AAC)L
tV(AAC)M2
tV(UAC)D
tW(CCA)G1
tW(CCA)G2
tW(CCA)P
tY(GUA)J1 SUP7
tY(GUA)M2 SUP8
tY(GUA)O SUP3
U2 snRNA (LSR1)

Cluster 2 (65)

CUT002
CUT083
CUT150
CUT332
CUT356
CUT442
CUT461
CUT547
CUT645
CUT827
SNR10
SNR128
SNR128/SNR190
SNR37
SNR4
SNR47
SNR51
SNR58
SNR64
SNR87
SUT004
SUT098
SUT167
SUT219/656
SUT274/698
SUT284/712
SUT367
SUT388
SUT471
SUT509
SUT543/115
SUT725
SUT834
tA(AGC)D
tD(GUC)B
tD(GUC)I2
tD(GUC)J1
tD(GUC)M
tE(CUC)I
tE(UUC)G2
tE(UUC)P
tF(GAA)B
tG(GCC)C (SUF16)
tG(GCC)D1
tG(GCC)D2
tH(GUG)E1
tH(GUG)E2
tH(GUG)M
tK(CUU)G3
tK(UUU)G1
tK(UUU)O
tL(CAA)K
tL(CAA)L
tL(UAA)L
tN(GUU)N1
tQ(UUG)L
tR(UCU)K
tS(AGA)L
tT(AGU)D
tV(AAC)H
tV(AAC)O
tV(CAC)D
tV(UAC)B
tW(CCA)M
tY(GUA)M1 (SUP5)

Cluster 3 (8)

SUT239/679
SUT431
SUT492
SUT718
tP(UGG)F (SUF9)
tT(AGU)O2
tX(XXX)D
tY(GUA)F1 (SUP11)

Cluster 4 (4)

CUT541/542/SUT503
SUT437
tL(CAA)N
tP(UGG)O2 (SUF11)
