# Supplementary material for: Large-scale profiling of noncoding RNA function in yeast
Source: PLoS Genet. 2018 Mar 12;14(3):e1007253. doi: 10.1371/journal.pgen.1007253 (PMC5864082; doi:10.1371/journal.pgen.1007253)
Supplement: S15 Table — (PDF) [file pgen.1007253.s015.pdf]

S15 TABLE

HAPLOID COLLECTION Genes listed in order of most haploproficient or haploinsufficient from top left down each column to bottom right.

Top 50 haploproficient on YPD plates

tD(GUC)J3	tE(UUC)K	tR(ACG)J	SUT304/730
tE(UUC)B	tR(UCU)J2	tL(UAA)N	tI(AAU)B
SNR75	tD(GUC)M	SNR47	tD(GUC)G2
tA(UGC)G	tF(GAA)P1	tV(AAC)M2	CUT468
SUT492	SUT479	SUT465	SNR81
SNR82	SUT798/387	SUT055	CUT474
SUT558	tL(GAG)G	tS(AGA)D2	SNR83
tF(GAA)N	SUT471	tR(ACG)D	SUT842/425
tG(UCC)N (SUF6)	tT(AGU)N1	tE(UUC)J	tK(CUU)E2
SUT129	SUT388	tD(GUC)L1	tH(GUG)G1
tD(GUC)J4	tA(UGC)O	SNR51/70/41	tI(UAU)D
tE(UUC)I	tL(UAG)L2	SUT722	
tV(AAC)J	tN(GUU)N2	SNR36	

Top 50 haploinsufficient on YPD plates

SUT233/CUT707	SUT457	tP(UGG)L	SNR17A
tL(CAA)A (SUP56)	tA(AGC)F	CUT103	SUT581
SUT339	CUT150	tF(GAA)M	CUT546
CUT816	SUT098	CUT213/690	SNR32
tT(AGU)N2	SUT493	CUT356	SUT014
CUT002	SUT361	tD(GUC)G1	SNR3
tI(AAU)L1	SUT347	tD(GUC)J1	SUT042
tV(AAC)M3	SUT086/CUT102	SNR61	tT(AGU)C
tV(AAC)G3	SUT725	tV(AAC)O	SUT035
SUT173	SNR10	SUT174	CUT296
tA(AGC)J	tG(GCC)G2	CUT436	CUT782
CUT102	SNR33	CUT424	
tD(GUC)L2	CUT298/771	SNR31	
