# Supplementary material for: Large-scale profiling of noncoding RNA function in yeast
Source: PLoS Genet. 2018 Mar 12;14(3):e1007253. doi: 10.1371/journal.pgen.1007253 (PMC5864082; doi:10.1371/journal.pgen.1007253)
Supplement: S16 Table — (PDF) [file pgen.1007253.s016.pdf]

S16 Table

Heterozygous tRNA deletions this collection	Homozygous tRNA deletions this collection	Mat a tRNA deletions this collection	Mat α tRNA deletions this collection	Mat α tRNA deletions Bloom-Ackermann et al
				tA(AGC)D
tA(AGC)F	tA(AGC)F	tA(AGC)F	tA(AGC)F	tA(AGC)F
tA(AGC)G	tA(AGC)G	tA(AGC)G	tA(AGC)G	tA(AGC)G
tA(AGC)H	tA(AGC)H	tA(AGC)H	tA(AGC)H	
tA(AGC)J	tA(AGC)J		tA(AGC)J	
				tA(AGC)K1
tA(AGC)K2		tA(AGC)K2		tA(AGC)K2
tA(AGC)L			tA(AGC)L	tA(AGC)L
tA(AGC)M1	tA(AGC)M1		tA(AGC)M1	tA(AGC)M1
tA(AGC)M2	tA(AGC)M2	tA(AGC)M2	tA(AGC)M2	tA(AGC)M2
tA(AGC)P				
	tA(UGC)A	tA(UGC)A	tA(UGC)A	tA(UGC)A
tA(UGC)E		tA(UGC)E		tA(UGC)E
tA(UGC)G	tA(UGC)G	tA(UGC)G	tA(UGC)G	tA(UGC)G
tA(UGC)L	tA(UGC)L	tA(UGC)L	tA(UGC)L	tA(UGC)L
tA(UGC)O	tA(UGC)O	tA(UGC)O	tA(UGC)O	
tC(GCA)B		tC(GCA)B		tC(GCA)B
tC(GCA)G		tC(GCA)G		
tC(GCA)P1	tC(GCA)P1	tC(GCA)P1	tC(GCA)P1	tC(GCA)P1
tC(GCA)P2		tC(GCA)P2		tC(GCA)P2
tD(GUC)B			tD(GUC)B	
tD(GUC)D				
	tD(GUC)G1	tD(GUC)G1	tD(GUC)G1	tD(GUC)G1
tD(GUC)G2	tD(GUC)G2	tD(GUC)G2	tD(GUC)G2	tD(GUC)G2
tD(GUC)I1	tD(GUC)I1	tD(GUC)I1	tD(GUC)I1	
tD(GUC)I2	tD(GUC)I2	tD(GUC)I2	tD(GUC)I2	
tD(GUC)J1	tD(GUC)J1	tD(GUC)J1	tD(GUC)J1	
tD(GUC)J2		tD(GUC)J2	tD(GUC)J2	
tD(GUC)J3	tD(GUC)J3	tD(GUC)J3	tD(GUC)J3	
tD(GUC)J4	tD(GUC)J4	tD(GUC)J4	tD(GUC)J4	tD(GUC)J4
tD(GUC)K	tD(GUC)K			tD(GUC)K
tD(GUC)L1	tD(GUC)L1	tD(GUC)L1	tD(GUC)L1	tD(GUC)L1
tD(GUC)L2	tD(GUC)L2	tD(GUC)L2	tD(GUC)L2	
tD(GUC)M	tD(GUC)M	tD(GUC)M	tD(GUC)M	tD(GUC)M
		tD(GUC)N		
tD(GUC)O	tD(GUC)O	tD(GUC)O	tD(GUC)O	tD(GUC)O
tE(CUC)D	tE(CUC)D	tE(CUC)D	tE(CUC)D	tE(CUC)D
tE(CUC)I	tE(CUC)I	tE(CUC)I	tE(CUC)I	tE(CUC)I
tE(UUC)B	tE(UUC)B	tE(UUC)B	tE(UUC)B	tE(UUC)B
tE(UUC)C	tE(UUC)C	tE(UUC)C		tE(UUC)C

tE(UUC)E1		tE(UUC)E1	tE(UUC)E1	tE(UUC)E1
tE(UUC)E2	tE(UUC)E2	tE(UUC)E2	tE(UUC)E2	
tE(UUC)E3			tE(UUC)E3	tE(UUC)E3
tE(UUC)G1 SOE1	tE(UUC)G1 SOE1	tE(UUC)G1 SOE1	tE(UUC)G1 SOE1	tE(UUC)G1
tE(UUC)G2		tE(UUC)G2	tE(UUC)G2	
tE(UUC)G3	tE(UUC)G3	tE(UUC)G3	tE(UUC)G3	
tE(UUC)I	tE(UUC)I	tE(UUC)I	tE(UUC)I	tE(UUC)I
tE(UUC)J	tE(UUC)J	tE(UUC)J	tE(UUC)J	tE(UUC)J
tE(UUC)K	tE(UUC)K	tE(UUC)K	tE(UUC)K	tE(UUC)K
tE(UUC)L	tE(UUC)L	tE(UUC)L	tE(UUC)L	tE(UUC)L
tE(UUC)M	tE(UUC)M	tE(UUC)M	tE(UUC)M	tE(UUC)M
tE(UUC)P	tE(UUC)P	tE(UUC)P	tE(UUC)P	tE(UUC)P
tF(GAA)B				
tF(GAA)D	tF(GAA)D	tF(GAA)D	tF(GAA)D	tF(GAA)D
tF(GAA)F	tF(GAA)F	tF(GAA)F	tF(GAA)F	tF(GAA)F
tF(GAA)G	tF(GAA)G	tF(GAA)G	tF(GAA)G	tF(GAA)G
tF(GAA)H1	tF(GAA)H1	tF(GAA)H1	tF(GAA)H1	tF(GAA)H1
tF(GAA)H2	tF(GAA)H2	tF(GAA)H2	tF(GAA)H2	tF(GAA)H2
tF(GAA)M	tF(GAA)M	tF(GAA)M	tF(GAA)M	tF(GAA)M
tF(GAA)N	tF(GAA)N	tF(GAA)N	tF(GAA)N	tF(GAA)N
tF(GAA)P1	tF(GAA)P1	tF(GAA)P1	tF(GAA)P1	tF(GAA)P1
tF(GAA)P2	tF(GAA)P2	tF(GAA)P2	tF(GAA)P2	tF(GAA)P2
tG(CCC)D SUF3	tG(CCC)D SUF3	tG(CCC)D SUF3	tG(CCC)D SUF3	tG(CCC)D
tG(CCC)O SUF5	tG(CCC)O SUF5	tG(CCC)O SUF5	tG(CCC)O SUF5	tG(CCC)O
tG(GCC)B				
tG(GCC)C SUF16	tG(GCC)C SUF16	tG(GCC)C SUF16	tG(GCC)C SUF16	tG(GCC)C
tG(GCC)D1	tG(GCC)D1	tG(GCC)D1	tG(GCC)D1	tG(GCC)D1
tG(GCC)D2	tG(GCC)D2	tG(GCC)D2	tG(GCC)D2	
tG(GCC)E	tG(GCC)E	tG(GCC)E	tG(GCC)E	tG(GCC)E
tG(GCC)F1 SUF20	tG(GCC)F1 SUF20	tG(GCC)F1 SUF20	tG(GCC)F1 SUF20	tG(GCC)F1
				tG(GCC)F2
tG(GCC)G1	tG(GCC)G1	tG(GCC)G1	tG(GCC)G1	tG(GCC)G1
		tG(GCC)G2	tG(GCC)G2	tG(GCC)G2
tG(GCC)J1	tG(GCC)J1	tG(GCC)J1	tG(GCC)J1	tG(GCC)J1
tG(GCC)J2		tG(GCC)J2	tG(GCC)J2	tG(GCC)J2
	tG(GCC)M	tG(GCC)M	tG(GCC)M	
tG(GCC)O1		tG(GCC)O1		tG(GCC)O1
tG(GCC)O2 SUF17	tG(GCC)O2 SUF17	tG(GCC)O2 SUF17		tG(GCC)O2
tG(GCC)P1	tG(GCC)P1	tG(GCC)P1	tG(GCC)P1	tG(GCC)P1
tG(GCC)P2	tG(GCC)P2	tG(GCC)P2	tG(GCC)P2	tG(GCC)P2
tG(UCC)G SUF4	tG(UCC)G SUF4	tG(UCC)G SUF4	tG(UCC)G SUF4	tG(UCC)G

tG(UCC)N SUF6	tG(UCC)N SUF6	tG(UCC)N SUF6	tG(UCC)N SUF6	tG(UCC)N
tG(UCC)O SUF1			tG(UCC)O SUF1	tG(UCC)O
tH(GUG)E1	tH(GUG)E1	tH(GUG)E1	tH(GUG)E1	
tH(GUG)E2			tH(GUG)E2	tH(GUG)E2
tH(GUG)G1	tH(GUG)G1	tH(GUG)G1	tH(GUG)G1	tH(GUG)G1
tH(GUG)G2	tH(GUG)G2	tH(GUG)G2	tH(GUG)G2	tH(GUG)G2
tH(GUG)H	tH(GUG)H	tH(GUG)H	tH(GUG)H	tH(GUG)H
tH(GUG)K	tH(GUG)K	tH(GUG)K	tH(GUG)K	
tH(GUG)M	tH(GUG)M	tH(GUG)M	tH(GUG)M	
tI(AAU)B	tI(AAU)B	tI(AAU)B	tI(AAU)B	
tI(AAU)D		tI(AAU)D	tI(AAU)D	tI(AAU)D
tI(AAU)E1	tI(AAU)E1	tI(AAU)E1	tI(AAU)E1	tI(AAU)E1
		tI(AAU)E2		tI(AAU)E2
tI(AAU)G	tI(AAU)G	tI(AAU)G	tI(AAU)G	tI(AAU)G
tI(AAU)I2	tI(AAU)I2	tI(AAU)I2	tI(AAU)I2	
tI(AAU)L1	tI(AAU)L1	tI(AAU)L1	tI(AAU)L1	tI(AAU)L1
tI(AAU)L2	tI(AAU)L2	tI(AAU)L2	tI(AAU)L2	tI(AAU)L2
tI(AAU)N1		tI(AAU)N1		tI(AAU)N1
tI(AAU)N2			tI(AAU)N2	
tI(AAU)P1	tI(AAU)P1	tI(AAU)P1	tI(AAU)P1	
tI(AAU)P2	tI(AAU)P2	tI(AAU)P2	tI(AAU)P2	
tI(UAU)D	tI(UAU)D	tI(UAU)D	tI(UAU)D	
tI(UAU)L				
tK(CUU)D1	tK(CUU)D1	tK(CUU)D1	tK(CUU)D1	tK(CUU)D1
tK(CUU)D2	tK(CUU)D2	tK(CUU)D2	tK(CUU)D2	
tK(CUU)E1	tK(CUU)E1	tK(CUU)E1	tK(CUU)E1	tK(CUU)E1
tK(CUU)E2	tK(CUU)E2	tK(CUU)E2	tK(CUU)E2	tK(CUU)E2
tK(CUU)F	tK(CUU)F	tK(CUU)F	tK(CUU)F	tK(CUU)F
tK(CUU)G1	tK(CUU)G1	tK(CUU)G1	tK(CUU)G1	tK(CUU)G1
tK(CUU)G2	tK(CUU)G2	tK(CUU)G2	tK(CUU)G2	tK(CUU)G2
tK(CUU)G3				tK(CUU)G3
	tK(CUU)I	tK(CUU)I	tK(CUU)I	
tK(CUU)J	tK(CUU)J	tK(CUU)J	tK(CUU)J	
tK(CUU)K				tK(CUU)K
tK(CUU)M	tK(CUU)M	tK(CUU)M	tK(CUU)M	tK(CUU)M
tK(CUU)P		tK(CUU)P	tK(CUU)P	tK(CUU)P
tK(UUU)D	tK(UUU)D	tK(UUU)D	tK(UUU)D	tK(UUU)D
				tK(UUU)G1
tK(UUU)G2	tK(UUU)G2	tK(UUU)G2	tK(UUU)G2	tK(UUU)G2
				tK(UUU)K
tK(UUU)L	tK(UUU)L	tK(UUU)L	tK(UUU)L	tK(UUU)L
tK(UUU)O			tK(UUU)O	tK(UUU)O
tK(UUU)P	tK(UUU)P	tK(UUU)P	tK(UUU)P	tK(UUU)P
tL(CAA)A SUP56	tL(CAA)A SUP56	tL(CAA)A SUP56	tL(CAA)A SUP56	tL(CAA)A

	tL(CAA)C SUP53	tL(CAA)C SUP53	tL(CAA)C SUP53	
tL(CAA)D	tL(CAA)D	tL(CAA)D	tL(CAA)D	
tL(CAA)G1	tL(CAA)G1	tL(CAA)G1	tL(CAA)G1	tL(CAA)G1
tL(CAA)G2 SUP54	tL(CAA)G2 SUP54	tL(CAA)G2 SUP54	tL(CAA)G2 SUP54	tL(CAA)G2
tL(CAA)G3	tL(CAA)G3	tL(CAA)G3	tL(CAA)G3	tL(CAA)G3
tL(CAA)K	tL(CAA)K	tL(CAA)K	tL(CAA)K	tL(CAA)K
tL(CAA)L	tL(CAA)L	tL(CAA)L	tL(CAA)L	tL(CAA)L
tL(CAA)M	tL(CAA)M	tL(CAA)M	tL(CAA)M	tL(CAA)M
tL(CAA)N				tL(CAA)N
tL(GAG)G	tL(GAG)G	tL(GAG)G	tL(GAG)G	tL(GAG)G
tL(UAA)B1		tL(UAA)B1		tL(UAA)B1
tL(UAA)B2	tL(UAA)B2	tL(UAA)B2	tL(UAA)B2	tL(UAA)B2
tL(UAA)D	tL(UAA)D	tL(UAA)D	tL(UAA)D	
tL(UAA)J SUP51	tL(UAA)J SUP51	tL(UAA)J SUP51	tL(UAA)J SUP51	tL(UAA)J
tL(UAA)K	tL(UAA)K	tL(UAA)K	tL(UAA)K	tL(UAA)K
	tL(UAA)L	tL(UAA)L	tL(UAA)L	tL(UAA)L
tL(UAA)N	tL(UAA)N	tL(UAA)N	tL(UAA)N	tL(UAA)N
tL(UAG)J		tL(UAG)J		tL(UAG)J
tL(UAG)L1			tL(UAG)L1	tL(UAG)L1
tL(UAG)L2	tL(UAG)L2	tL(UAG)L2	tL(UAG)L2	tL(UAG)L2
tN(GUU)C	tM(CAU)C	tM(CAU)C	tM(CAU)C	tM(CAU)C
				tM(CAU)D
				tM(CAU)E
				tM(CAU)J1
				tM(CAU)J3
				tM(CAU)M
				tM(CAU)O2
				tN(GUU)C
tN(GUU)F	tN(GUU)F	tN(GUU)F	tN(GUU)F	tN(GUU)F
tN(GUU)G	tN(GUU)G	tN(GUU)G	tN(GUU)G	tN(GUU)G
tN(GUU)K	tN(GUU)K	tN(GUU)K	tN(GUU)K	tN(GUU)K
tN(GUU)L	tN(GUU)L	tN(GUU)L	tN(GUU)L	
tN(GUU)N1	tN(GUU)N1	tN(GUU)N1	tN(GUU)N1	
tN(GUU)N2	tN(GUU)N2	tN(GUU)N2	tN(GUU)N2	tN(GUU)N2
tN(GUU)O1	tN(GUU)O1	tN(GUU)O1	tN(GUU)O1	tN(GUU)O1
tN(GUU)O2			tN(GUU)O2	tN(GUU)O2
				tN(GUU)P
tP(AGG)C SUF2		tP(AGG)C SUF2		tP(AGG)C
tP(AGG)N SUF10	tP(AGG)N SUF10	tP(AGG)N SUF10	tP(AGG)N SUF10	tP(AGG)N
tP(UGG)A TRN1				tP(UGG)A
tP(UGG)F SUF9	tP(UGG)F SUF9	tP(UGG)F SUF9	tP(UGG)F SUF9	tP(UGG)F

tP(UGG)H SUF8	tP(UGG)H SUF8	tP(UGG)H SUF8	tP(UGG)H SUF8	tP(UGG)H
tP(UGG)L	tP(UGG)L	tP(UGG)L	tP(UGG)L	tP(UGG)L
tP(UGG)M SUF7	tP(UGG)M SUF7	tP(UGG)M SUF7	tP(UGG)M SUF7	tP(UGG)M
				tP(UGG)N1
tP(UGG)N2	tP(UGG)N2	tP(UGG)N2	tP(UGG)N2	tP(UGG)N2
tP(UGG)O1	tP(UGG)O1	tP(UGG)O1	tP(UGG)O1	
tP(UGG)O2 SUF11	tP(UGG)O2 SUF11	tP(UGG)O2 SUF11	tP(UGG)O2 SUF11	tP(UGG)O2
tP(UGG)O3				tP(UGG)O3
tQ(CUG)M CDC65		tQ(CUG)M CDC65		tQ(CUG)M
tQ(UUG)B	tQ(UUG)B	tQ(UUG)B	tQ(UUG)B	tQ(UUG)B
tQ(UUG)C		tQ(UUG)C		tQ(UUG)C
tQ(UUG)D1	tQ(UUG)D1	tQ(UUG)D1	tQ(UUG)D1	tQ(UUG)D1
tQ(UUG)D2				
tQ(UUG)D3	tQ(UUG)D3	tQ(UUG)D3	tQ(UUG)D3	tQ(UUG)D3
tQ(UUG)E1	tQ(UUG)E1	tQ(UUG)E1	tQ(UUG)E1	
tQ(UUG)E2	tQ(UUG)E2	tQ(UUG)E2	tQ(UUG)E2	tQ(UUG)E2
tQ(UUG)H	tQ(UUG)H	tQ(UUG)H	tQ(UUG)H	tQ(UUG)H
tQ(UUG)L	tQ(UUG)L	tQ(UUG)L	tQ(UUG)L	tQ(UUG)L
tR(ACG)D	tR(ACG)D	tR(ACG)D	tR(ACG)D	tR(ACG)D
tR(ACG)E	tR(ACG)E	tR(ACG)E	tR(ACG)E	
tR(ACG)J	tR(ACG)J	tR(ACG)J	tR(ACG)J	tR(ACG)J
tR(ACG)K	tR(ACG)K		tR(ACG)K	tR(ACG)K
tR(ACG)L		tR(ACG)L		tR(ACG)L
tR(ACG)O	tR(ACG)O	tR(ACG)O	tR(ACG)O	tR(ACG)O
tR(CCG)L TRR4				tR(CCG)L
tR(CCU)J HSX1				tR(CCU)J
tR(UCU)B				
tR(UCU)D				
tR(UCU)E				tR(UCU)E
tR(UCU)G1				tR(UCU)G1
tR(UCU)G2	tR(UCU)G2	tR(UCU)G2	tR(UCU)G2	
tR(UCU)G3	tR(UCU)G3	tR(UCU)G3	tR(UCU)G3	
tR(UCU)J1	tR(UCU)J1	tR(UCU)J1	tR(UCU)J1	
tR(UCU)J2	tR(UCU)J2	tR(UCU)J2	tR(UCU)J2	
tR(UCU)K	tR(UCU)K	tR(UCU)K	tR(UCU)K	tR(UCU)K
tR(UCU)M1	tR(UCU)M1	tR(UCU)M1	tR(UCU)M1	tR(UCU)M1
tR(UCU)M2	tR(UCU)M2	tR(UCU)M2	tR(UCU)M2	tR(UCU)M2
		tS(AGA)A		tS(AGA)A
tS(AGA)B	tS(AGA)B	tS(AGA)B	tS(AGA)B	tS(AGA)B
tS(AGA)D1	tS(AGA)D1	tS(AGA)D1	tS(AGA)D1	tS(AGA)D1
tS(AGA)D2		tS(AGA)D2	tS(AGA)D2	tS(AGA)D2
tS(AGA)D3	tS(AGA)D3	tS(AGA)D3	tS(AGA)D3	tS(AGA)D3

tS(AGA)E				
tS(AGA)G	tS(AGA)G	tS(AGA)G	tS(AGA)G	tS(AGA)G
tS(AGA)H	tS(AGA)H	tS(AGA)H	tS(AGA)H	tS(AGA)H
tS(AGA)J	tS(AGA)J	tS(AGA)J	tS(AGA)J	tS(AGA)J
tS(AGA)L				
tS(AGA)M	tS(AGA)M	tS(AGA)M	tS(AGA)M	tS(AGA)M
tS(CGA)C SUP61				tS(CGA)C
tS(GCU)F				tS(GCU)F
tS(GCU)L	tS(GCU)L	tS(GCU)L	tS(GCU)L	tS(GCU)L
tS(GCU)O	tS(GCU)O	tS(GCU)O	tS(GCU)O	tS(GCU)O
				tS(UGA)E
tS(UGA)I SUP17	tS(UGA)I SUP17	tS(UGA)I SUP17	tS(UGA)I SUP17	tS(UGA)I
tS(UGA)P SUP16	tS(UGA)P SUP16	tS(UGA)P SUP16	tS(UGA)P SUP16	tS(UGA)P
tT(AGU)B	tT(AGU)B	tT(AGU)B	tT(AGU)B	tT(AGU)B
tT(AGU)C	tT(AGU)C	tT(AGU)C	tT(AGU)C	tT(AGU)C
tT(AGU)D				tT(AGU)D
tT(AGU)H	tT(AGU)H	tT(AGU)H	tT(AGU)H	tT(AGU)H
tT(AGU)I1	tT(AGU)I1	tT(AGU)I1	tT(AGU)I1	
tT(AGU)I2	tT(AGU)I2	tT(AGU)I2	tT(AGU)I2	tT(AGU)I2
tT(AGU)J				tT(AGU)J
tT(AGU)N1	tT(AGU)N1	tT(AGU)N1	tT(AGU)N1	tT(AGU)N1
tT(AGU)N2	tT(AGU)N2	tT(AGU)N2	tT(AGU)N2	tT(AGU)N2
tT(AGU)O1	tT(AGU)O1	tT(AGU)O1	tT(AGU)O1	
	tT(AGU)O2	tT(AGU)O2	tT(AGU)O2	tT(AGU)O2
tT(CGU)K TRT2	tT(CGU)K TRN1	tT(CGU)K TRN1	tT(CGU)K TRN1	tT(CGU)K
tT(UGU)G1	tT(UGU)G1	tT(UGU)G1	tT(UGU)G1	
tT(UGU)G2	tT(UGU)G2	tT(UGU)G2	tT(UGU)G2	tT(UGU)G2
tT(UGU)H	tT(UGU)H		tT(UGU)H	
tT(UGU)P	tT(UGU)P	tT(UGU)P	tT(UGU)P	
tV(AAC)E1	tV(AAC)E1	tV(AAC)E1	tV(AAC)E1	tV(AAC)E1
tV(AAC)E2	tV(AAC)E2	tV(AAC)E2	tV(AAC)E2	tV(AAC)E2
				tV(AAC)G1
tV(AAC)G2	tV(AAC)G2	tV(AAC)G2	tV(AAC)G2	tV(AAC)G2
tV(AAC)G3	tV(AAC)G3	tV(AAC)G3	tV(AAC)G3	
tV(AAC)H				
tV(AAC)J	tV(AAC)J	tV(AAC)J	tV(AAC)J	tV(AAC)J
tV(AAC)K1				tV(AAC)K1
tV(AAC)K2	tV(AAC)K2	tV(AAC)K2	tV(AAC)K2	tV(AAC)K2
tV(AAC)L		tV(AAC)L		tV(AAC)L
tV(AAC)M1		tV(AAC)M1	tV(AAC)M1	tV(AAC)M1
tV(AAC)M2	tV(AAC)M2	tV(AAC)M2	tV(AAC)M2	tV(AAC)M2
tV(AAC)M3		tV(AAC)M3	tV(AAC)M3	tV(AAC)M3
tV(AAC)O	tV(AAC)O	tV(AAC)O	tV(AAC)O	tV(AAC)O
tV(CAC)D	tV(CAC)D	tV(CAC)D	tV(CAC)D	

	tV(UAC)B	tV(UAC)B	tV(UAC)B	tV(UAC)B
tV(UAC)D				tV(UAC)D
tW(CCA)G1	tW(CCA)G1	tW(CCA)G1	tW(CCA)G1	tW(CCA)G1
tW(CCA)G2	tW(CCA)G2	tW(CCA)G2	tW(CCA)G2	tW(CCA)G2
tW(CCA)J		tW(CCA)J		tW(CCA)J
tW(CCA)K	tW(CCA)K	tW(CCA)K	tW(CCA)K	tW(CCA)K
tW(CCA)M	tW(CCA)M	tW(CCA)M	tW(CCA)M	tW(CCA)M
tX(XXX)D	tX(XXX)D	tX(XXX)D	tX(XXX)D	
tY(GUA)D SUP2		tY(GUA)D SUP2	tY(GUA)D SUP2	
			tY(GUA)F1 SUP11	
tY(GUA)F2 SUP6				
tY(GUA)J1 SUP7	tY(GUA)J1 SUP7	tY(GUA)J1 SUP7	tY(GUA)J1 SUP7	tY(GUA)J1
tY(GUA)J2 SUP4				tY(GUA)J2
tY(GUA)M1 SUP5	tY(GUA)M1 SUP5	tY(GUA)M1 SUP5	tY(GUA)M1 SUP5	tY(GUA)M1
tY(GUA)M2 SUP8	tY(GUA)M2 SUP8	tY(GUA)M2 SUP8	tY(GUA)M2 SUP8	tY(GUA)M2
tY(GUA)O SUP3	tY(GUA)O SUP3	tY(GUA)O SUP3	tY(GUA)O SUP3	
