# Supplementary material for: Large-scale profiling of noncoding RNA function in yeast
Source: PLoS Genet. 2018 Mar 12;14(3):e1007253. doi: 10.1371/journal.pgen.1007253 (PMC5864082; doi:10.1371/journal.pgen.1007253)
Supplement: S17 Table — (PDF) [file pgen.1007253.s017.pdf]

**S17 Table. Strains**

| Source | Strain | Genotype | Use |
|---|---|---|---|
| ATCC 4040002 | BY4741 | *MATa; his3Δ1; leu2Δ0; met15Δ0; ura3Δ0* | Background strain for ncRNA deletions (Brachmann et al. 1998) |
| ATCC 4040004 | BY4742 | *MATα; his3Δ1; leu2Δ0; lys2Δ0; ura3Δ0* | Background strain for ncRNA deletions (Brachmann et al. 1998) |
| ATCC 4040005 | BY4743 | *MATa/MATα; his3Δ1/his3Δ1; leu2Δ0 /leu2Δ0; met15Δ0/MET15; LYS2/lys2Δ0; ura3Δ0/ura3Δ0* | Background strain for ncRNA deletions (Brachmann et al. 1998) |
| C. Stirling | TR2 | *MATa*; trp1-289; his3-600; ura3-52; lys2-801a; ade2-101 | Mating type testing (Parker et al. 1988) |
| C. Stirling | TR3 | *MATα;* trp1-289; his3-600; ura3-52; lys2-801a; ade2-101 | Mating type testing (Parker et al. 1988) |
| EUROSCARF | CML476 | *MATa;* ura3-52; leu2Δ1; his3Δ200; GAL2; CMVp(tetR'-SSN6)::LEU2; trp1::tTA | Regulated expression (Yen et al. 2003) |

**References**


Brachmann CB, Davies A, Cost GJ, Caputo E, Li J, Hieter P, Boeke JD. 1998. Designer deletion strains derived from Saccharomyces cerevisiae S288C: a useful set of strains and plasmids for PCR-mediated gene disruption and other applications. *Yeast* **14**: 115-132.

Parker R, Simmons T, Shuster EO, Siliciano PG, Guthrie C. 1988. Genetic analysis of small nuclear RNAs in Saccharomyces cerevisiae: viable sextuple mutant. *Mol Cell Biol* **8**: 3150-3159.

Yen K, Gitsham P, Wishart J, Oliver SG, Zhang N. 2003. An improved tetO promoter replacement system for regulating the expression of yeast genes. *Yeast* **20**: 1255-1262.
