# Supplementary material for: Large-scale profiling of noncoding RNA function in yeast
Source: PLoS Genet. 2018 Mar 12;14(3):e1007253. doi: 10.1371/journal.pgen.1007253 (PMC5864082; doi:10.1371/journal.pgen.1007253)
Supplement: S18 Table — (PDF) [file pgen.1007253.s018.pdf]

S18 Table. Primer list

Primer Name	Sequence (5' to 3')
SEC4F-Bam	CGGGATCCTAGACATATATGTACATCTAAAC
SEC4B-Eco	AATAAAAGTGCTGAATATTAAAAG
RUF20F-Bam	CGGGATCCTAGACTTTTTCAAGTTCCTTATCC
RUF20B-Xba	GCTCTAGACGCTGGTCAAGAACGTTTCCGGAC
SEC4F-T7	GCTAATACGACTCACTATAGGGTCAACAGCAATTTGATTT
SEC4B-prob	ATGTCAGGCTTGAGAACTGTTTCTG
RUF20F-T7	GCTAATACGACTCACTATAGGGCATACTTCTACTGTTGTTCTCTGC
RUF20B-prob	ACGTGGGTTTTTTTTTCGAATTGAGTGATTATG
RUF20_P1	CTTCCTTCAGGAATTTTTTCAAGCCCTCATAATTAATATAAGAACCGTACGCTGCAGGTCGACGG
RUF20_P2	CCTGTATGGTTGCATAATCACTCAATTCGAAAAAAAAACCCACGTATAGGCCACTAGTGGATCTG
SEC4F	GCTTCATCCGGTAATGGAAAGAGC
SEC4B1	GAACCTCGTTTTCCTGTTGCAC
SEC4B2	GCCTGATGAAAATACCTTCCAGAGC
SEC4B3	GCTGAAAAAGTTAGTTAGTGTGAGAAGTTTG
SEC2B4	GTGACATCTAATATGACTAAT
SEC4B5	ATAACCTGCGAATATTGAG
SEC4B6	CCAGTTCACGATTAATTCTC
SEC4B7	CCGCTCCCACTATTGATGC
SEC4B8	CGTTCTTAGCACTGGACTCGA
TUB2F	CATATCTCGACAGGTCAGTGTG
TUB2B1	CCCTGATCTGCGTAATATTGC
TUB2B2	GCTCCAAGTGCTTCAATCCTAG
TUB2B3	CATTGTATGGTTCGACAACGG
RUF20FQ	GCGCAAGTAGTTGAATAGTGG
RUF20BQ	CCCTGGGAATTCTTATCCAAG
SEC4FQ	GTTCTCTCGTCTGTCACATC
SEC4BQ	GGTAAAGCTGCAACTTTGGG
HAP4F	ATATTCCATTGGCGCCTGTA
HAP4R	TTGGTGTTGTTGGCAGGTAG
KTI12F	TATTGGCGGGGTAAGTAACG
KTI12R	AAGCGGACCGATCCTATCTT
DPS1F	TGGTTCCAGTGTGTTTGAGG
DPS1R	TCTTCGAAAGCCATTTCCAT
PRP3F	GCCTCGTTGATCTTTTCAGG
PRP3R	GCCGAAGAAAAGCTAGCAGA
EMP46F	GCTGAAGCAAGGAAATGAGG
EMP46R	CGCTCTCTCCCAACTTATCG
GAL2F	GTAGAAGACGCCAAGCGTTC
GAL2R	CCAGCCAGTTTTTCAGCTTC
RPL3F	GACTGGGCTCGTGAACATTT
RPL3R	AACACCTTCGAAACCGTGAC
SUT690F	CGACAGTAAAAGCAGCAAAACA
SUT690R	AGTTCCCCTGGTCCGTAAAC
ACT1F	CGGTGATGGTGTTACTCACG
ACT1B	GGCCAAATCGATTCTCAAAA

Gal1.for	GGCCCCCCCTCGAGGCGGATTAGAAGCCGCCGAG
Gal1.rev	ATATCAAGCTTATCGATACCGCTCCTTGACGTTAAAGTATAGAGGTATATTAACAATTTTTTG
Cyc1.for	GCCCGGGGCAAATTAAAGCCTTCGAGCGT
Cyc1.rev	ACTAGTGGATCCTCATGTAATTAGTTATGTCACGCTTACATTCACG
SUT527.for	AAGGAGCGGTATCGATAAAAAGTTGGCAAGAAGAAAGAAATGACTCT
SUT527.rev	CTGCAGGAATTCGATATCATTTTTCTCAATATTCGCAGGTTATTTTTCCTTTATATATATACATTGT
SUT075.for	AAGGAGCGGTATCGATAAACAATTCACAGTGCCTGTCCAG
SUT075.rev	CTGCAGGAATTCGATATCAGTCATCACAGGTGGTGCTCC
SUT367.for	AAGGAGCGGTATCGATACGCTCGTATCAGCCACTTACG
SUT367.rev	CTGCAGGAATTCGATATCATTTTCATGGACGATGATCACTGAAAAAAAAAAAAAAAAAAT
SUT259.for	AAGGAGCGGTATCGATAAATGGGTCACGTGATCTATATTCGAAAGG
SUT259.rev	CTGCAGGAATTCGATATCATGGGAAGGGCTTAACTAATCTCGG
SUT691.for	AAGGAGCGGTATCGATAAAGGGCTTAACTAATCTCGGTTCG
SUT691.rev	CTGCAGGAATTCGATATCAGCAATTTCTTTTTCTATTAGTAGCTAAAAATGGGTCAC
SUT527 antisense.for	AAGGAGCGGTATCGATATCTTTAGCAGGTTTGTCCACTAATCTACG
SUT527 antisense.rev	CTGCAGGAATTCGATATCAAATAACAGGCAGACGTGAGAGAAGAA
SUT075 antisense.for	AAGGAGCGGTATCGATAGTCATCACAGGTGGTGCTCC
SUT075 antisense.rev	CTGCAGGAATTCGATATCAAACAATTCACAGTGCCTGTCCAG
SUT367 antisense.for	AAGGAGCGGTATCGATATTTTCATGGACGATGATCACTGAAAAAAAAAAAAAAAAAAT
SUT367 antisense.rev	CTGCAGGAATTCGATATCACGCTCGTATCAGCCACTTACG
SUT259 antisense.for	AAGGAGCGGTATCGATATGGGAAGGGCTTAACTAATCTCGG
SUT259 antisense.rev	CTGCAGGAATTCGATATCAAATGGGTCACGTGATCTATATTCGAAAGG
SUT691 antisense.for	AAGGAGCGGTATCGATAGCAATTTCTTTTTCTATTAGTAGCTAAAAATGGGTCAC
SUT691 antisense.rev	CTGCAGGAATTCGATATCAAAGGGCTTAACTAATCTCGGTTCG
