# Supplementary figures and images for: Large-scale profiling of noncoding RNA function in yeast
Source: PLoS Genet. 2018 Mar 12;14(3):e1007253. doi: 10.1371/journal.pgen.1007253 (PMC5864082; doi:10.1371/journal.pgen.1007253)

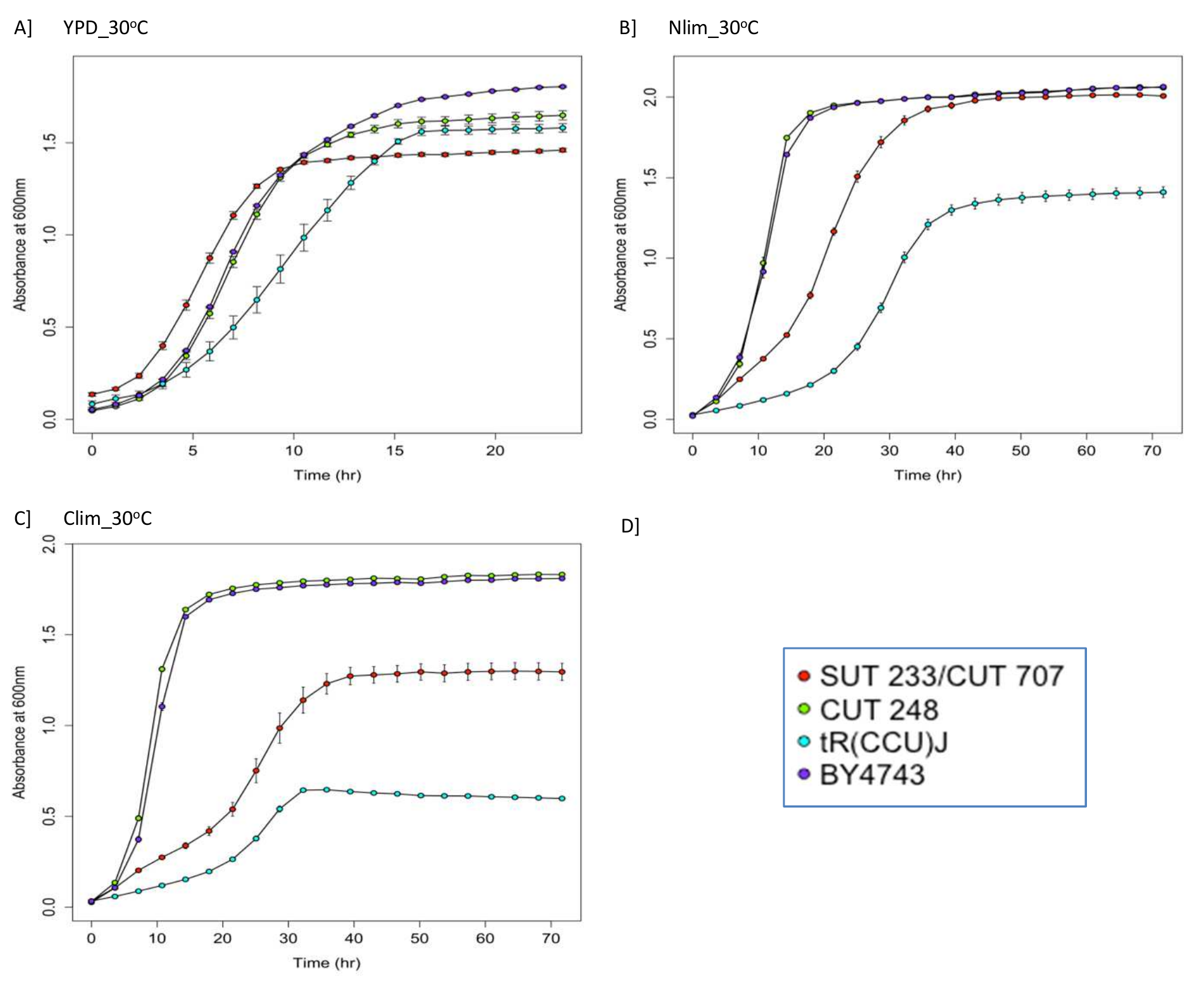

Supplement: S1 Fig — ncRNA deletion strains were tested individually for their fitness in YPD (A), nitrogen-limited (B) and carbon-limited (C) chemically defined F1 media at 30°C. Growth curves shown are expressed as the mean growth from three replicates of three independent biological strains for each deletion and from six replicate cultures for the wild type (BY4743) strain. Limitations in nitrogen or carbon sources are indicated as Nlim (B) and Clim (C), respectively. Error bars are present in all points and are indicated as the standard deviations from the replicates. All strains included in the growth assays are represented by different colors as described in panel D. (TIF) [file pgen.1007253.s019.tif]

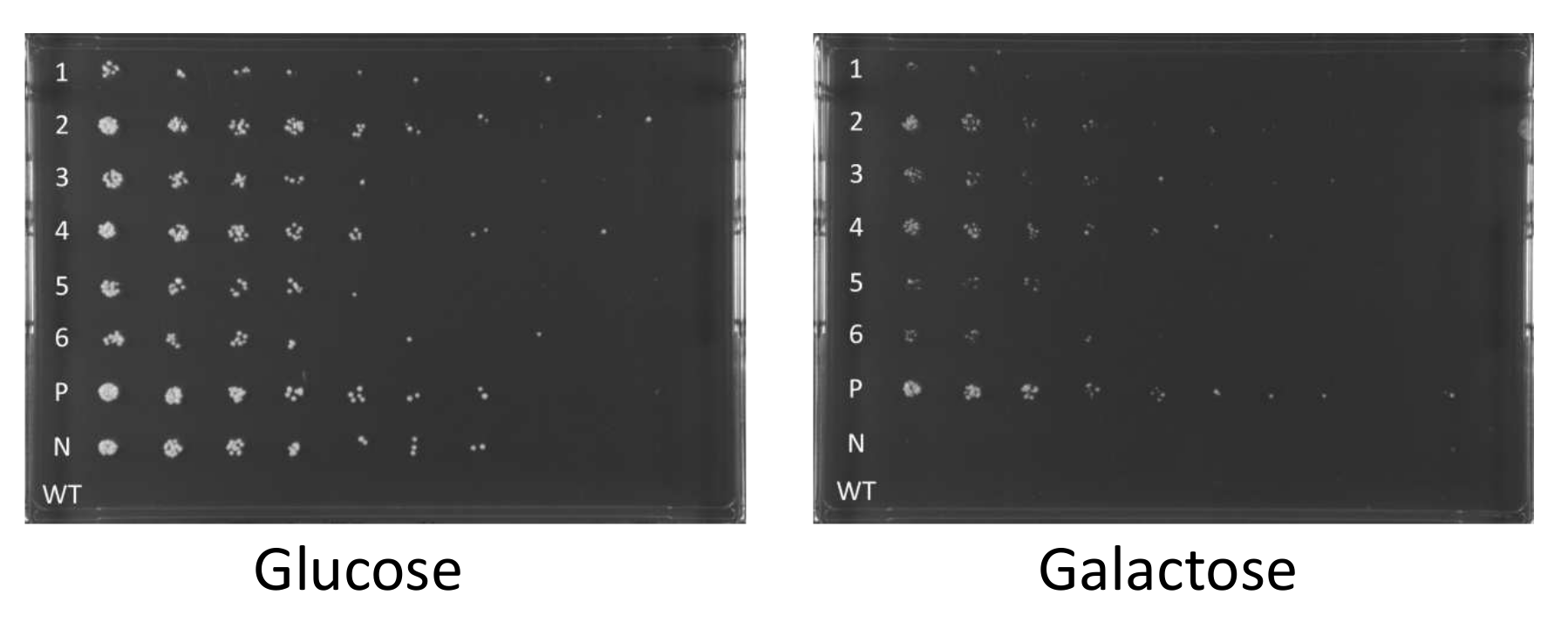

Supplement: S2 Fig — The RNA sequence for CUT248 was cloned into a yeast expression vector under control of the yeast GAL1 promoter. The vector was transformed into the wild-type BY4741 haploid strain and six independent single colonies (1–6) were spotted by serial dilution on SD-Ura plates with either glucose or galactose containing plates. Plates were incubated at 30°C for 48hrs. The expression vector alone (P), the vector expressing the gene WWM1 known to cause lethality when overexpressed (N) and the BY4741 strain alone were also spotted by serial dilution on the same plates. (TIF) [file pgen.1007253.s020.tif]

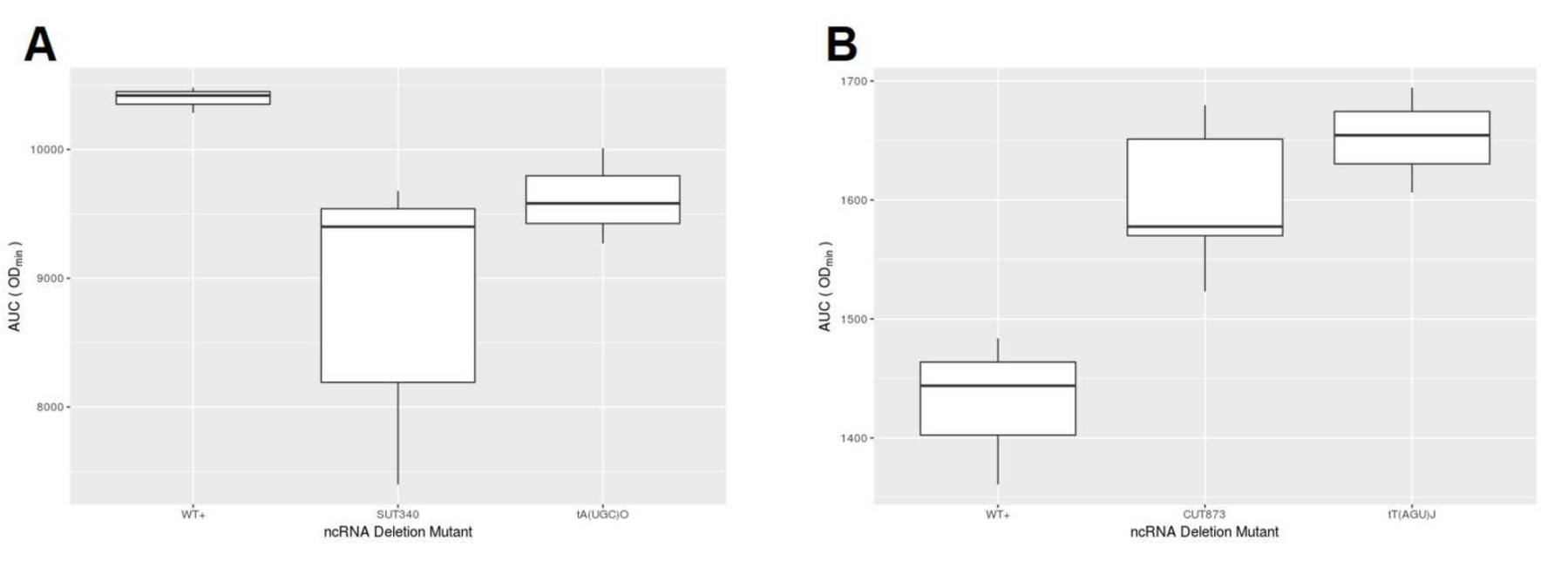

Supplement: S3 Fig — Monoculture validation, in a microplate reader, of four strains identified as being haplo-insufficient or haplo-proficient during competition experiments in continuous culture. Box plots are constructed using the area under curve (AUC) as parameter. (A) SUT340 and tA(UGC)O heterozygote diploid deletion strains grown in C-limited F1 media at 36°C. (B) CUT873 and tT(AGU)J heterozygote diploid deletion strains grown in C-limited F1 media at 30°C. (TIF) [file pgen.1007253.s021.tif]

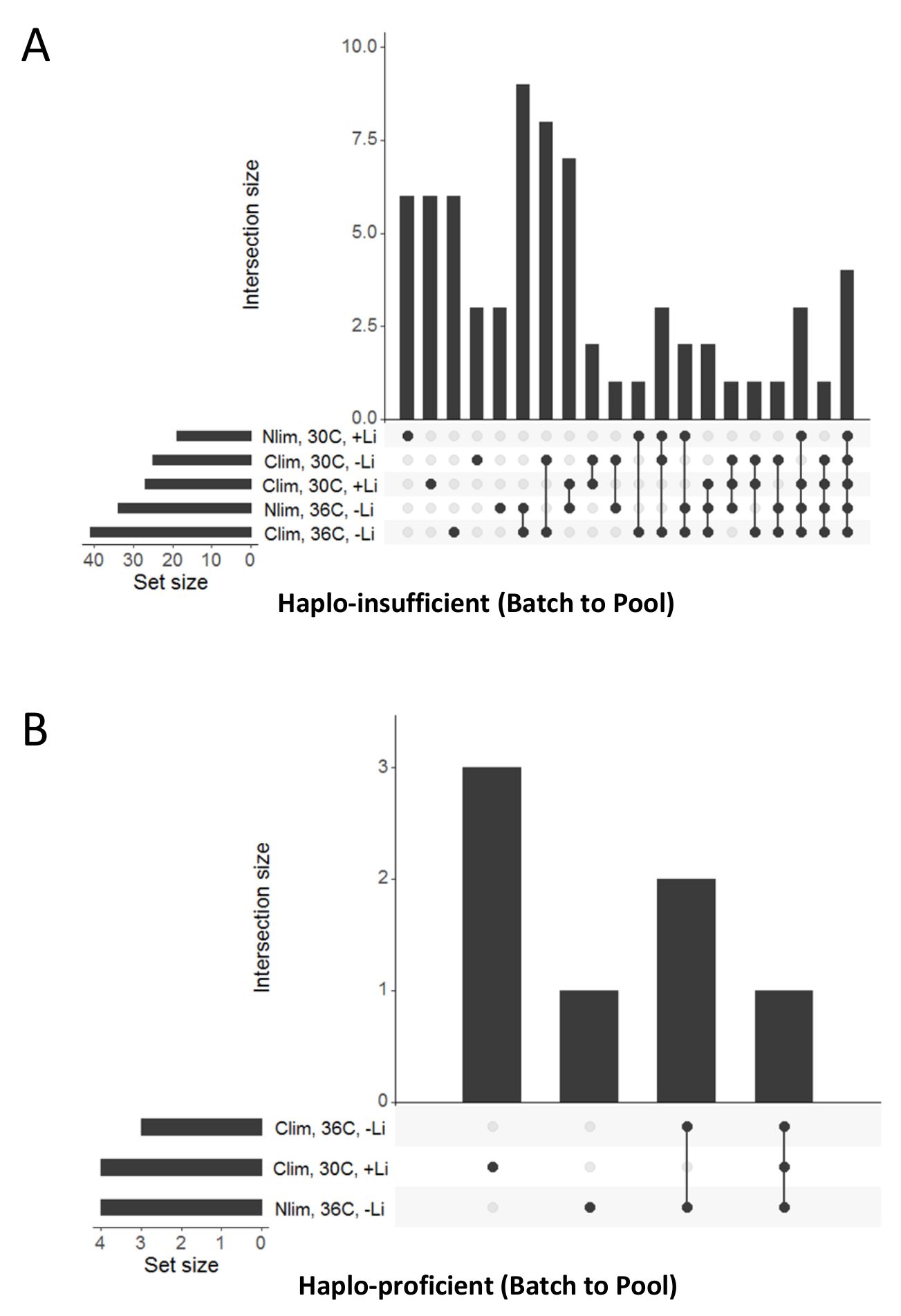

Supplement: S4 Fig — Upset plots to visualize common haplo-insufficient (A) and haplo-proficient (B) fitness profiles between different conditions in Batch to Pool experiments. Horizontal bars for each condition shows the total number of strains with significant fitness differences at Log2 fold change greater than 1.50 and p-value less than 0.05. Connected black circles indicate common profiles across different conditions with vertical bars showing the number of intersections. (TIF) [file pgen.1007253.s022.tif]

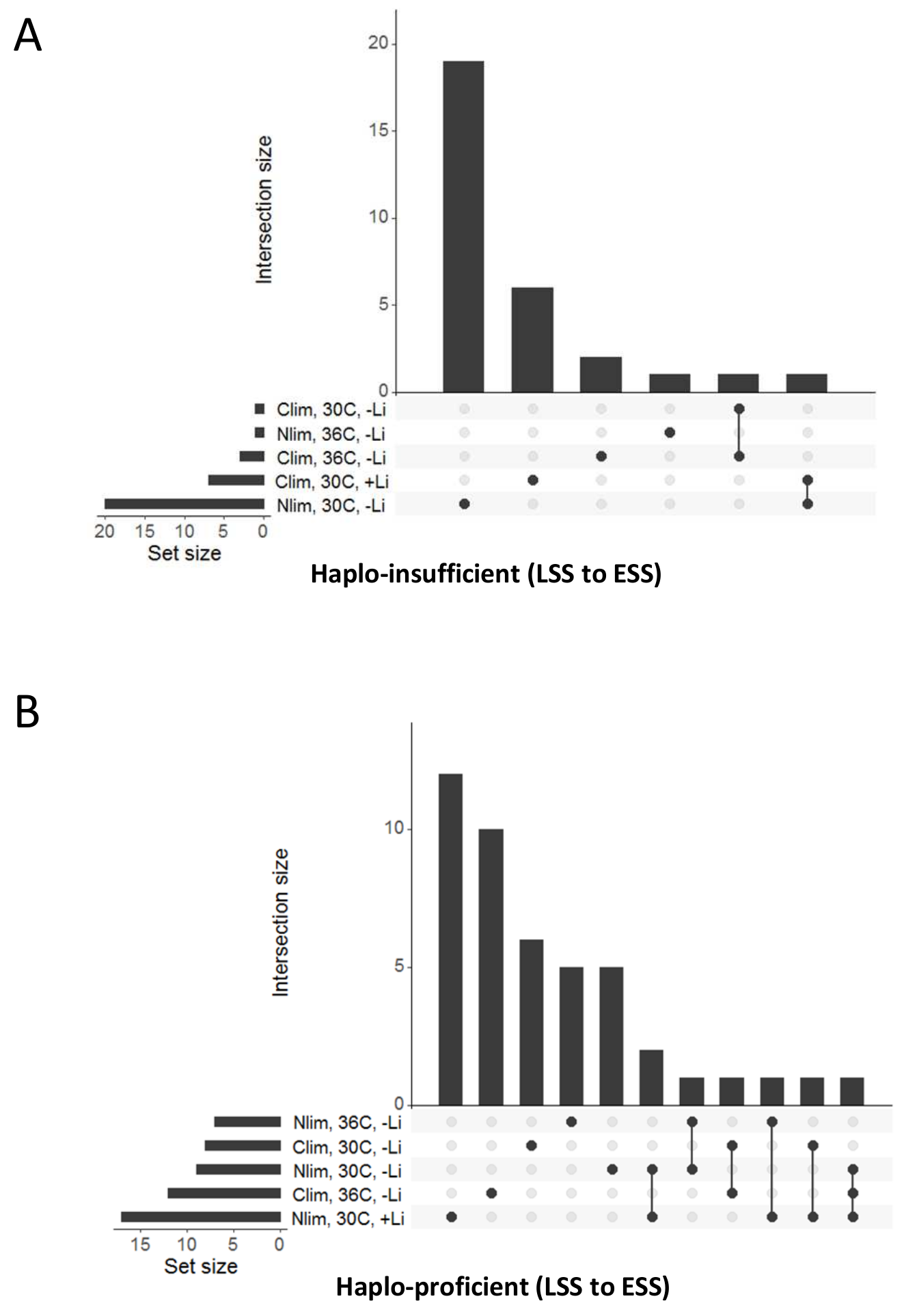

Supplement: S5 Fig — Upset plots to visualize common haplo-insufficient (A) and haplo-proficient (B) fitness profiles between different conditions in LSS to ESS experiments. Horizontal bars for each condition shows the total number of strains with significant fitness differences at Log2 fold change greater than 1.50 and p-value less than 0.05. Connected black circles indicate common profiles across different conditions with vertical bars showing the number of intersections. (TIF) [file pgen.1007253.s023.tif]

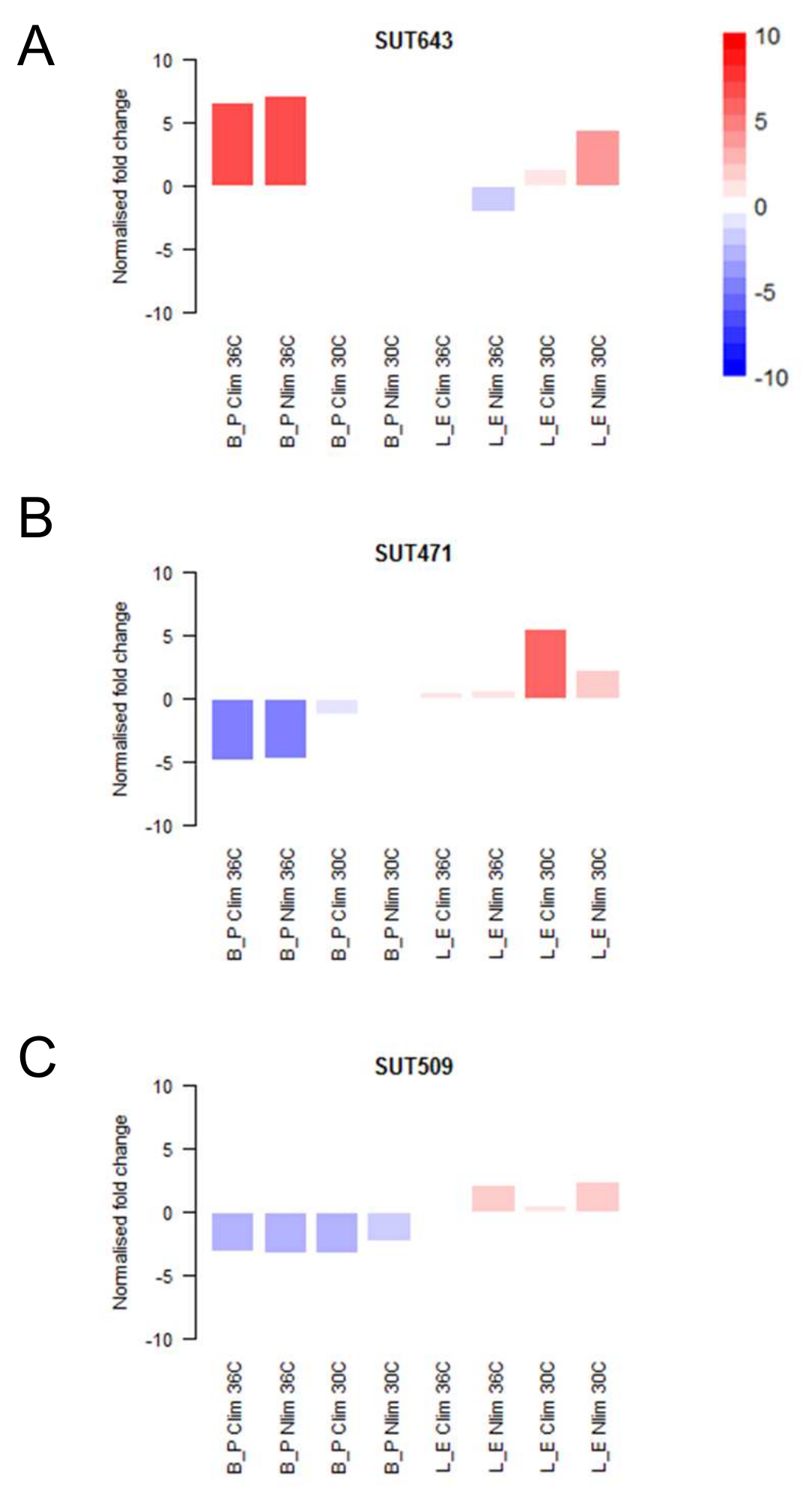

Supplement: S6 Fig — Fitness profiles of selected ncRNA deletion strains (A) SUT643; (B) SUT471; (C) SUT509. Heights represent Log2 fold change between batch and pool or late and early steady state across the eight growth conditions (B_P:comparison between batch and pool; L_E: comparison between late and early steady state; Clim: carbon-limited medium; Nlim: nitrogen-limited medium). Colours represent direction of fitness changes. Haplo-insufficiency is shown in blue, and haplo-proficiency is shown in bright red. (TIF) [file pgen.1007253.s024.tif]

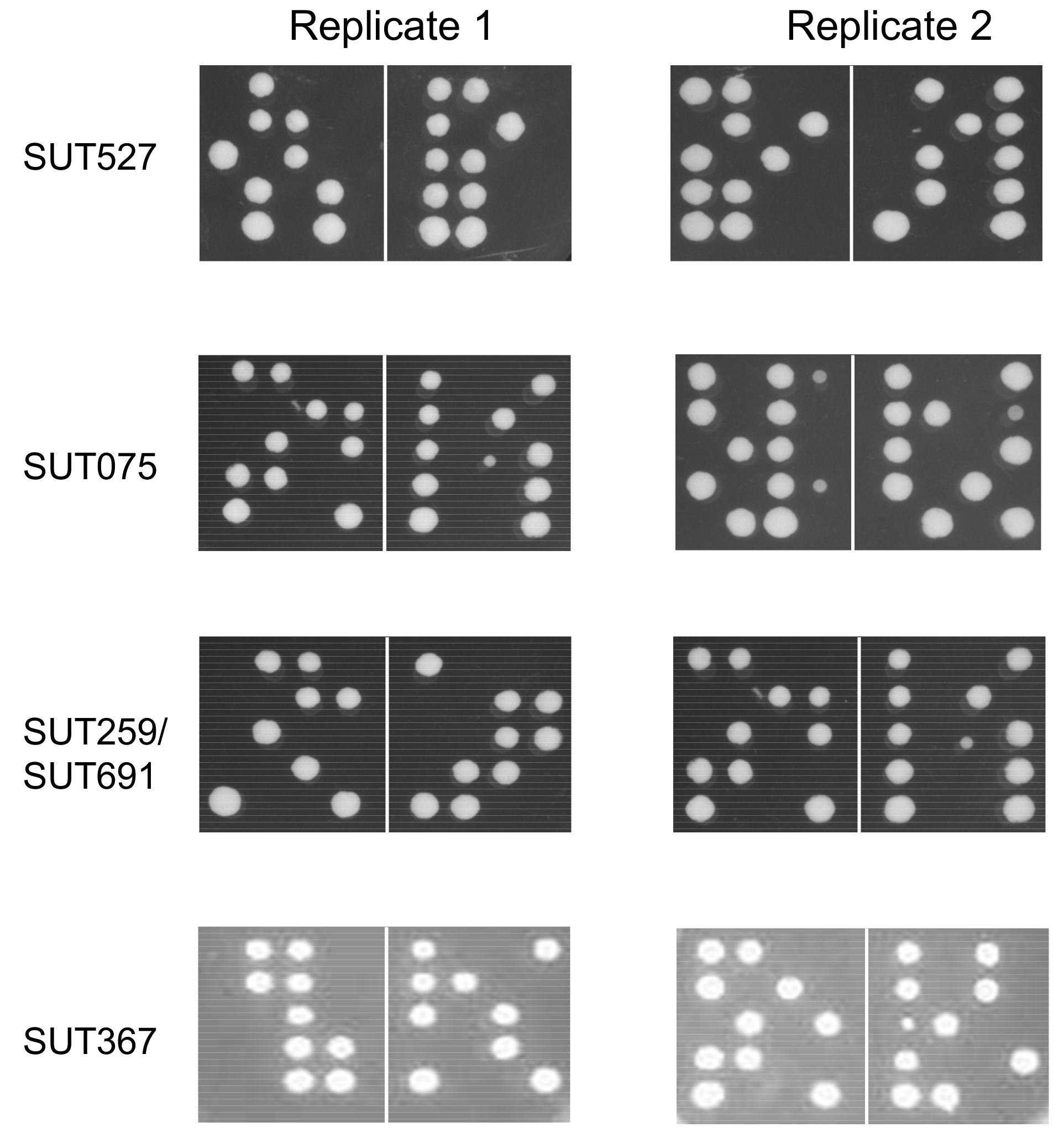

Supplement: S7 Fig — Two additional biological replicates of the diploid knockout strains found to be essential with the first replicate were sporulated and tetrads dissected to determine essentiality. All replicates displayed a pattern (2 viable, 2 lethal) consistent with all the ncRNA deletions being essential. (TIF) [file pgen.1007253.s025.tif]

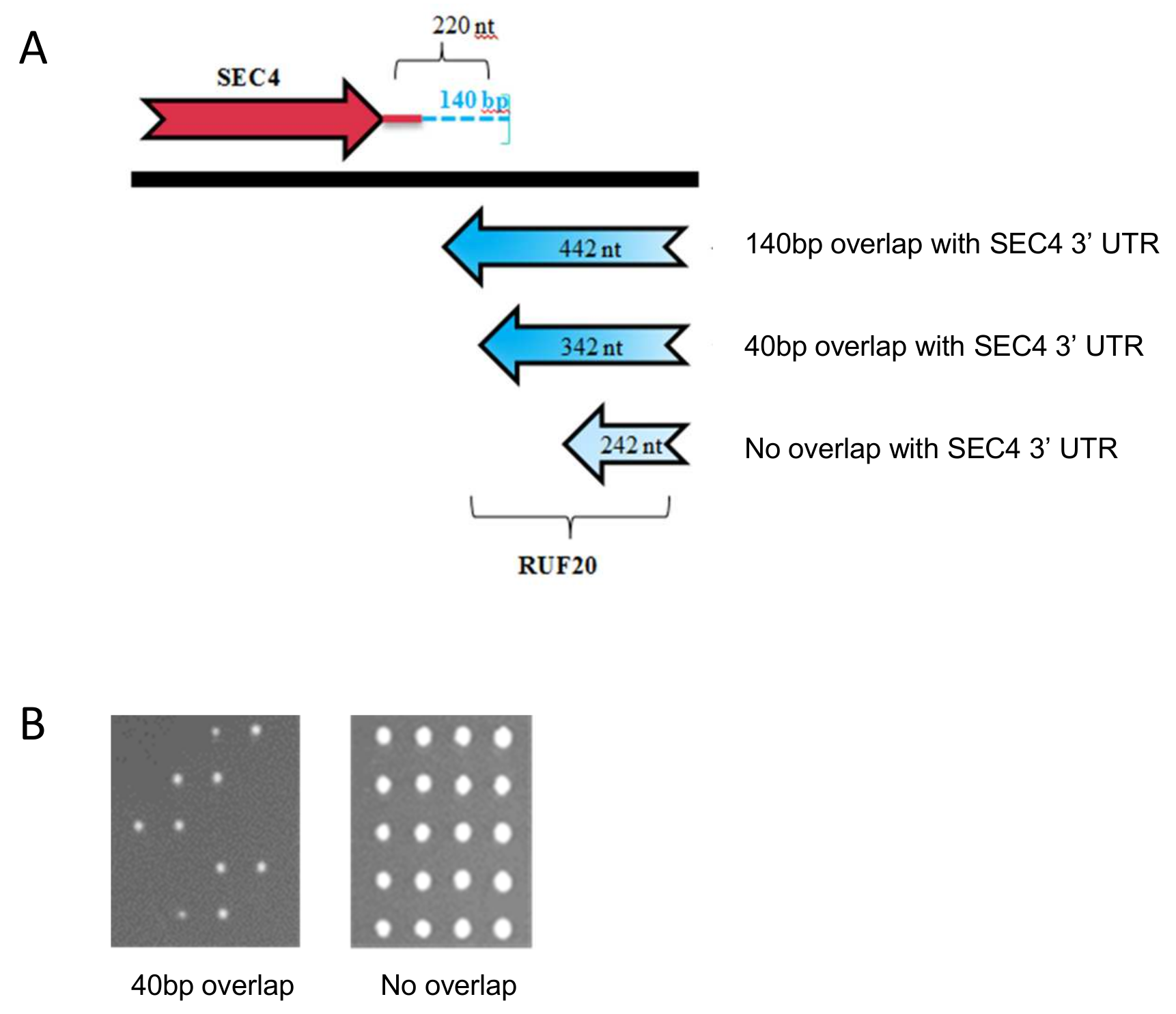

Supplement: S8 Fig — (A) The different lengths of SUT527/RUF20 deletions are represented as blue arrows containing the number of nucleotides deleted. Arrows with a blue gradient indicate that the deletion has disrupted the 3’ UTR of SEC4. (B) Only two viable spores grew after diploid sporulation and dissection for the 342nt deletion of SUT527/RUF20, indicating that this region of SUT527/RUF20 is still essential (left panel). Four viable spores grew after diploid sporulation and tetrad dissection for the 242nt deletion of SUT527/RUF20, indicating that it is not essential (right panel). (TIF) [file pgen.1007253.s026.tif]

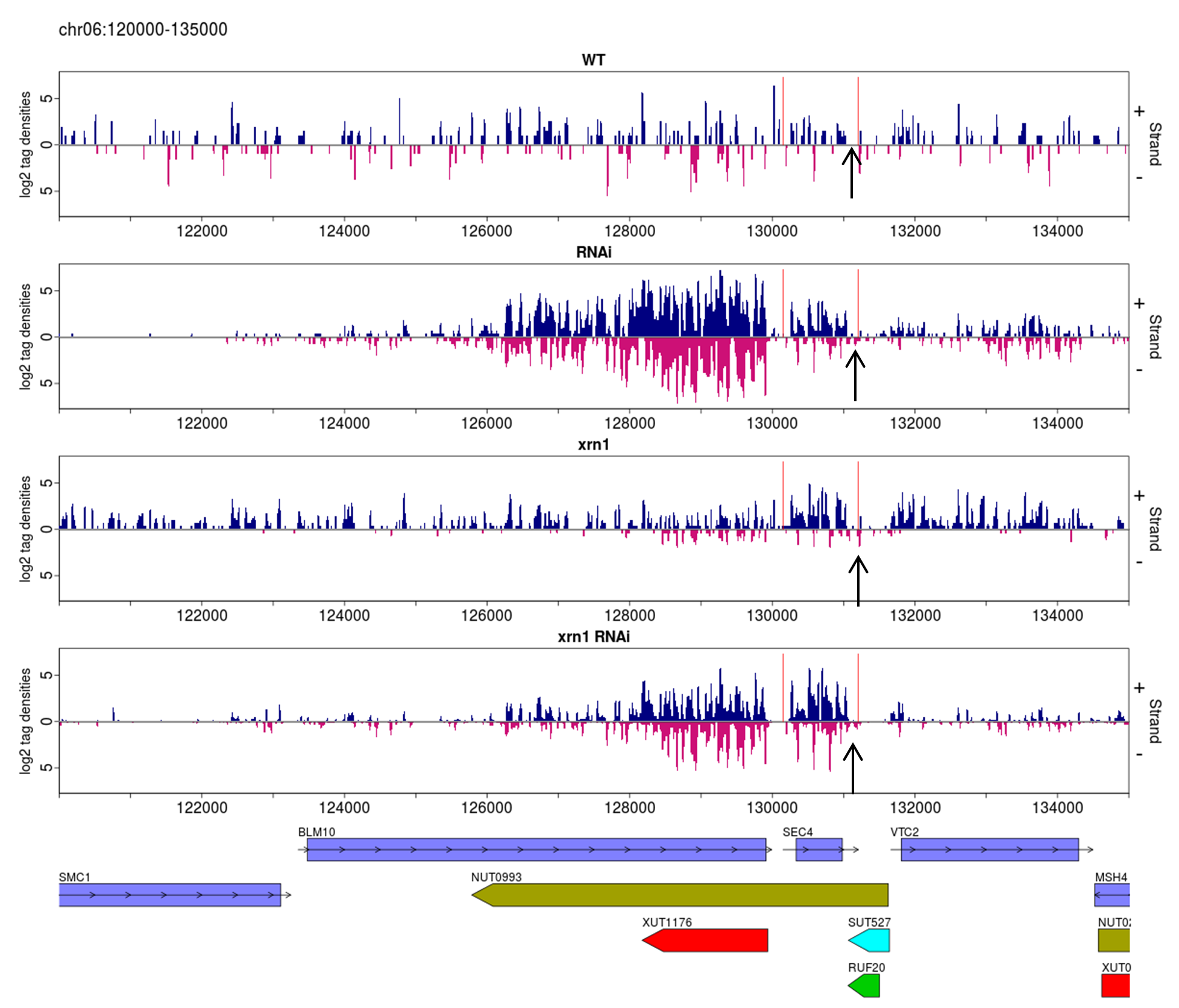

Supplement: S9 Fig — Screen shot from genome browser for visualization of processed small RNA-seq data for Genome-wide mapping of dsRNA from Wery et al. 2016, Molecular Cell 61:3790–392 (http://vm-gb.curie.fr/mw2). Red lines define the limits of the SEC4 transcript. Black arrows point to the region of overlap between SEC4 3’ UTR and SUT527. dsRNA (red peaks) is detected upon RNAi reconstitution. (TIF) [file pgen.1007253.s027.tif]

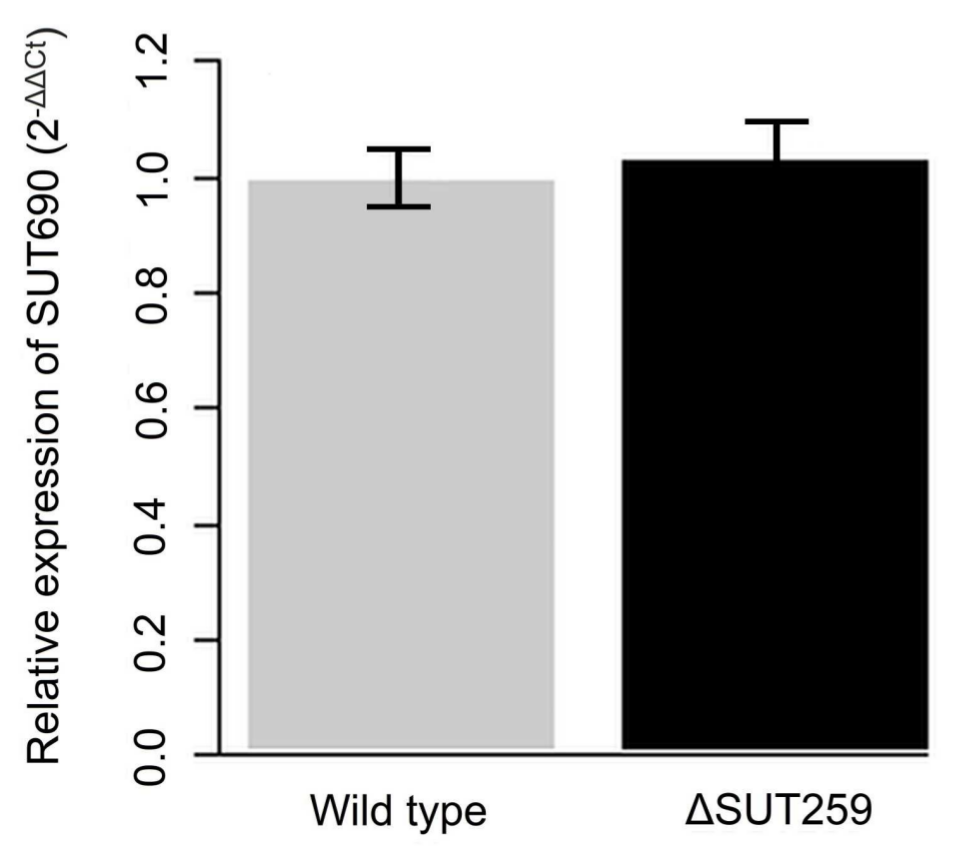

Supplement: S10 Fig — Real time PCR results to measure expression levels of SUT690 in the ΔSUT259/691 heterozygote diploid deletion strain. The relative expression of SUT690 in the wild-type background is represented by a shaded grey bar and as a shaded black bar in ΔSUT259/691 strain background. Using the ΔΔCт method and ACT1 as a reference gene, the fold change (2^) in expression, relative to the wild-type was calculated. Error bars are calculated using each of the three independent biological samples. P values calculated using the Welch two sample t-test. There is no significant difference (p = 0.72) in SUT690 expression between the wild type and ΔSUT259/691 strains. (TIF) [file pgen.1007253.s028.tif]
